# Evolution of multivalent supramolecular assemblies of aptamers with target-defined spatial organization

**DOI:** 10.1038/s41565-025-01939-8

**Published:** 2025-06-06

**Authors:** Artem Kononenko, Vincenzo Caroprese, Yoan Duhoo, Cem Tekin, Maartje M. C. Bastings

**Affiliations:** 1 Programmable Biomaterials Laboratory, Institute of Materials, Interfaculty Bioengineering Institute, School of Engineering, Ecole Polytechnique Fédérale de Lausanne, Lausanne, Switzerland; 2 Protein Production and Structure Core Facility, School of Life Sciences, Ecole Polytechnique Fédérale de Lausanne, Lausanne, Switzerland

**Keywords:** Organizing materials with DNA, Nanofabrication and nanopatterning, Molecular self-assembly

## Abstract

Rapid identification of neutralizing molecules against new and mutating viruses is key to efficiently combating biorisk. Current binder identification techniques use a monovalent library of potential binders. Interestingly, proteins on pathogens are often homo-oligomeric—for example, the SARS-CoV-2 spike protein is a homotrimer. Here we describe a simple strategy, MEDUSA (multivalent evolved DNA-based supramolecular assembly), to evolve multivalent assemblies of aptamers with precise interligand spacing and three-fold symmetry, mirroring the geometric structure of many viral capsid proteins. MEDUSA allowed the selection of potent SARS-CoV-2 spike binders structurally distinct from any known aptamers. Decoupling the geometric and structural rigidity contributions toward selectivity made it possible to connect form to function, as demonstrated by the design of tunable fluorescent sensors. This approach offers a blueprint for targeting geometrically defined pathogen structures and developing rapid-response tools for emerging pathogens.

## Abstract (web summary)

Expanding aptamer binder space through tunable directed coevolution of supramolecular scaffolds helps discover nucleic-acid-based multivalent target binders capable of synergistic engagement that cannot be obtained through monovalent selection.

## Main

An ever-growing demand for strong and selective binders for diagnostic and therapeutic applications is incentivizing the development of new classes of binding molecules and efficient high-throughput-screening methods for their discovery^1^. High-affinity binders represent an effective class of molecules for interrogating protein–protein interactions (PPIs)^1,2^, including those of therapeutic and diagnostic importance. Antibodies are natural examples of high-affinity, specific binders^3^, although they require specialized production and come with inherent biological side activity that triggers immune signalling^4^. Several synthetic methods can identify new high-affinity binders to a target of interest, making it possible to circumvent many of the drawbacks of antibodies and reduce animal use. These methods include display technologies^5–7^, directed evolution^8^ and systematic evolution of ligands by exponential enrichment (SELEX)^9,10^ for nucleic acid aptamers. Their common strategy is to select the most potent binder(s) from highly diverse libraries based on binding affinity. However, strong monovalent binding does not always translate into functional activity, as is the case when binders interact away from the active interface^11^.

Many clinically important viruses display large trimeric glycoprotein complexes on their surfaces, which are crucial for cell-entry processes^12^. Notably, the trimeric configuration of all class I fusion proteins is conserved across many domains of animal viruses^13^, and therefore offers a useful guideline for the rational design of targeting and sensing agents. Arranging ligands into a target-specific spatial organization makes it possible to create potent and functionally active binding compounds by increasing the effective concentration of ligands at their cognate binding sites^14,15^. Although challenging on classical nanomaterial formulations such as liposomes, dendrimers or gold nanoparticles, DNA nanotechnology offers a convenient platform for programmable ligand presentation with excellent control over geometry, spacing and valency^16^. Various designer multivalent nanostructures have been successfully applied for displaying two-dimensional arrays of manifold ligands: dengue envelope protein domain III-targeting aptamers^17^, insulin^18^, CpG^19^, CD95L^20^, PD1^21^, etc. However, all designs rely on existing binders that have well-documented structural evidence of binding epitopes. Unfortunately, many viral proteins lack this advantage.

Although new ligands can be generated de novo through directed evolution processes^22,23^, their subsequent multimerization is not trivial and often requires extensive optimization of both the linker and its attachment site^24^. In biological systems, molecules coevolve within interconnected assemblies of diverse molecular species, resulting in synergistic interactions and the emergence of new functional capabilities^25–27^. In contrast, directed evolution has largely been performed within a monovalent framework, in which library members cannot collectively enhance each other’s properties as an integrated assembly. An alternative strategy is to evolve binders in the multivalent setting and thereby promote selection of ligands capable of cooperatively engaging the target within the context of a user-defined ligand arrangement. Recent efforts have indeed focused on integrating multivalency-driven selection into directed evolution platforms, aiming to select bivalent constructs against monomeric protein targets^28–30^. Notably, classical phage display represents an example of directed evolution of higher-valency binding systems^31^, yet it offers only limited control over geometry and spacing between ligands.

In this paper, we expand the directed evolution toolbox by introducing MEDUSA (multivalent evolved DNA-based supramolecular assembly), a hybrid molecular modality consisting of an ad hoc multimerization scaffold with three binding units mirroring the trimeric configuration of viral fusion proteins. This molecular framework can be adopted for the directed evolution of large combinatorial libraries of binding units to bias the selection regime towards the enrichment of binders capable of synergistic multivalent engagement with the target. The identified hits displayed remarkable structural diversity, distant from those selected in classic monovalent fashion, and only showed functional and selective target binding when presented in a multivalent context. Analysis of the structural contributions of the central multimerization scaffold made it possible to understand the balance of geometry versus flexibility and develop a proof-of-principle multivalent sensor. Together, we present a modular technology to identify new classes of aptamers for oligomeric targets through selection of geometric target-tailored multivalent assemblies.

## Results

### Principles of multivalency-biased affinity selection

**Fig. 1:**
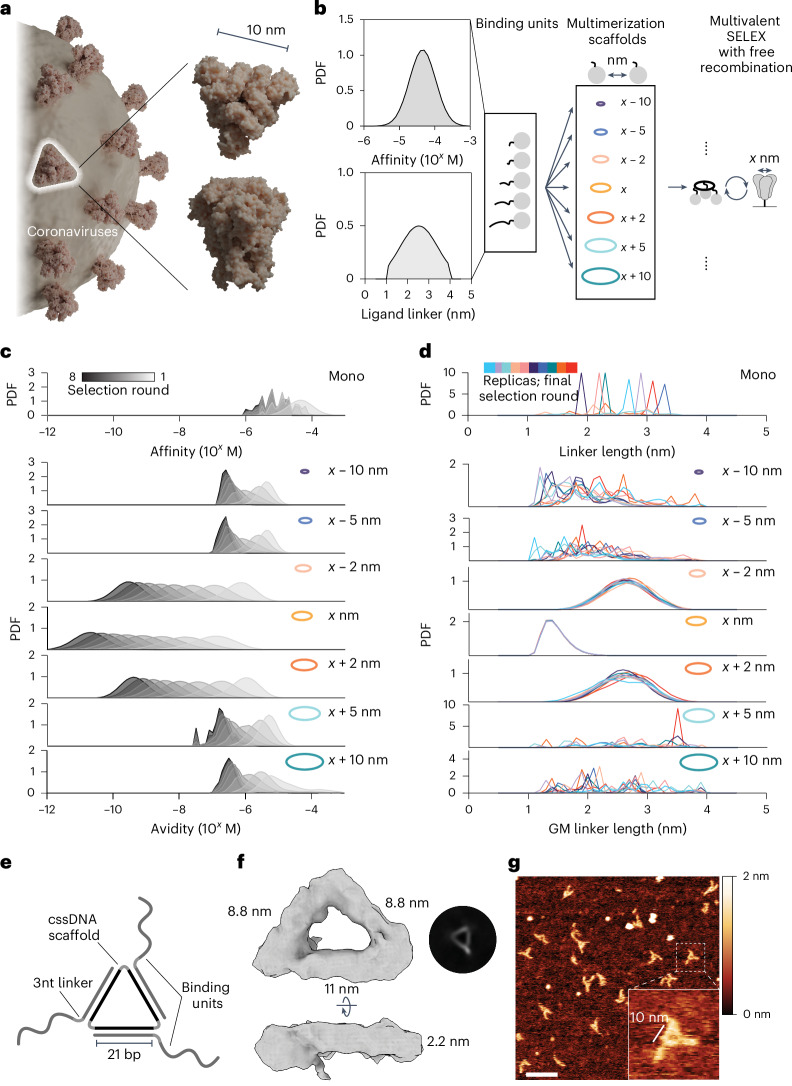
Exploring the consequences of geometric target (mis)matching. **a**, Large trimeric glycoprotein complexes are characteristic of many human pathogens as exemplified by SARS-CoV-2 spike protein. Figure created in Blender (https://www.blender.org), using PDB 3JCL as a spike model. **b**, Schematic for Gillespie simulation of multivalent selection against a homotrimeric target. A random library of binding units with varying spatial tolerances for simultaneous target engagement and a set of multimerization scaffolds with increasing interligand spacing were tested. The initial probability density function (PDF) of affinities (top) and linkers (bottom) for the binding unit library is given. **c**, Simulation results of multivalent selection for libraries prepared with different multivalent scaffolds show that matching scaffold geometry facilitates the selection of assemblies with the highest avidity. All Gillespie simulations were performed in 10 replicates (*n* = 10); results are shown as average PDFs. **d**, The PDF of linkers in the binding unit library in the final round of multivalent selection is determined by the scaffold geometry. GM, geometric mean. **e**, Schematic representation of MEDUSA’s molecular architecture. **f**, Cryo-EM 3D class-average electron density of a designed MEDUSA. The respective Fourier shell correlation analysis is provided in Supplementary Fig. 4. **g**, Representative atomic force microscopy image of MEDUSA. Scale bar, 20 nm. Source data

In nature, molecules often coevolve within interconnected, multicomponent assemblies, resulting in the collective development of interfaces. As an analogy, we envisioned the use of a target-tailored multimerization scaffold to evolve synthetic polymers against an oligomeric target, yielding multivalent binders with cooperative binding behaviour within a designed multivalent assembly. Many clinically relevant target proteins exist as multimeric complexes, supporting the application of target-tailored evolution to identify new binders. The evolutionary-conserved trimeric structure of viral capsid glycoprotein complexes is emblematic of many human pathogens such as retroviruses^32^, coronaviruses^33^ and orthomyxoviruses^34^. As a target for the development of our multivalent selection methodology, we opted to use the trimeric SARS-CoV-2 spike protein, arguably one of the most representative members of viral class I fusion proteins (Fig. 1a).

The performance of multivalent binders relies heavily on the scaffold joining the ligands because this determines their orientation and overall structural flexibility^24^. Previous modelling results suggest that maximal binding affinity can be achieved when the binder’s core is rigid and matches the target’s size, and the linkers have an average end-to-end distance slightly longer than the distance between the binder’s core and cognate binding epitopes^15^. Building on these results, we developed a multiscale computational framework for simulating multivalent SELEX (Supplementary Fig. 1) through modelling stochastic selection dynamics^35^. Simulations used a fixed triangular target with dimensions derived from the spike protein (marked as *x*) to examine selections conducted with various scaffold geometries (Fig. 1b) and explored how binding affinities and linker lengths evolved under different spatial constraints.

In the multivalent SELEX process, ligand and linker distributions adapt over successive rounds of selection, yielding the best avidities when the scaffold geometry closely aligns with the target (Fig. 1c). Minor deviations in scaffold size (for example, <*x* − 2 nm or >*x* + 2 nm) quickly limit avidities to the micromolar range, demonstrating the critical role of scaffold geometry in enabling effective multivalent binding. Scaffold geometry directly influences linker length selection (Fig. 1d): when the scaffold geometry matches the target geometry, libraries converge toward minimal linker lengths that facilitate multivalent engagement by precise alignment with binding epitopes. In contrast, monovalent libraries exhibit a random distribution of linker lengths after selection because they are not subjected to spatial constraints. Scaffolds deviating slightly from the optimal geometry still support multivalent binding by selecting longer linkers. However, scaffolds outside the spatial tolerance of the ligand library fail to favour multivalent interactions. In these cases, selection reverts to a monovalent-like regime, without selective pressure on linker length. These findings underscore the significance of scaffold geometry in determining spatial constraints, providing predictive design principles for optimizing ligand selection in multivalent systems.

Based on simulation results, we established several design principles for the scaffold and binding unit library. First, the scaffold geometry should closely match the target’s size and shape to maximize the contribution of avidity. Second, for matching scaffolds, linkers should be of minimal length to not disrupt the advantages of a geometric fit. We explored several potential scaffold structures that would satisfy these criteria and allow for the display of binding units at ~10 nm pairwise distances within a trivalent framework, reflecting the overall dimensions and rotational symmetry of SARS-CoV-2 spike protein (Supplementary Fig. 2a,b). We settled on a cyclic single-stranded DNA (cssDNA) scaffold due to its nuclease stability, size and ease of preparation (Supplementary Figs. 2c and 3). The architecture of MEDUSA emerges from the three 21-nucleotide binding unit-hybridization regions, separated by 2 T hinges within cssDNA (Fig. 1e–g). This configuration allows the binding units to be positioned at every second turn of the DNA, displaying them on the same face of the scaffold. Following the second design principle, we opted to keep the linkers short, placing 3 nucleotides (~1.6 nm (ref. ^36^)) between the scaffold-hybridization region and the binding region.

### Multivalent assemblies of functionalized nucleic acids

Besides structural programmability, a major advantage of DNA as scaffold is its compatibility with numerous evolvable polymers, such as DNA, RNA^9,10^, slow off-rate modified aptamers^37^, highly side-chain-functionalized nucleic acid polymers^38^, PNA^39^ and acyclic l-threoninol nucleic acid^40^. This compatibility makes it possible to construct hybrid nanostructures that merge the versatile design potential of DNA with the chemical diversity of proteins and beyond^38,41^. We combine the cssDNA scaffold with functionalized nucleic acid polymer (FNAP) binding units, which together create the MEDUSA for affinity evolution of hybrid multivalent targeting nanomaterials. FNAP is a base-modified nucleic acid polymer, produced via DNA-ligase-mediated polymerization of trinucleotide building blocks on the DNA template. In the FNAP library design, we partially sacrifice the density of side-chain functionalization in favour of sequence diversity, allowing the library to sample a broader range of secondary structures of the polymer. Structure–activity relationship studies provide the rationale for this choice because often only few side chains are essential for target binding^38^. A convenient property of MEDUSA is its excellent nuclease resistance, so critical for applications in biological fluids, ensured by the cyclic scaffold and enzyme-inhibiting nucleotide modifications at the 5′ position^42,43^.

**Fig. 2:**
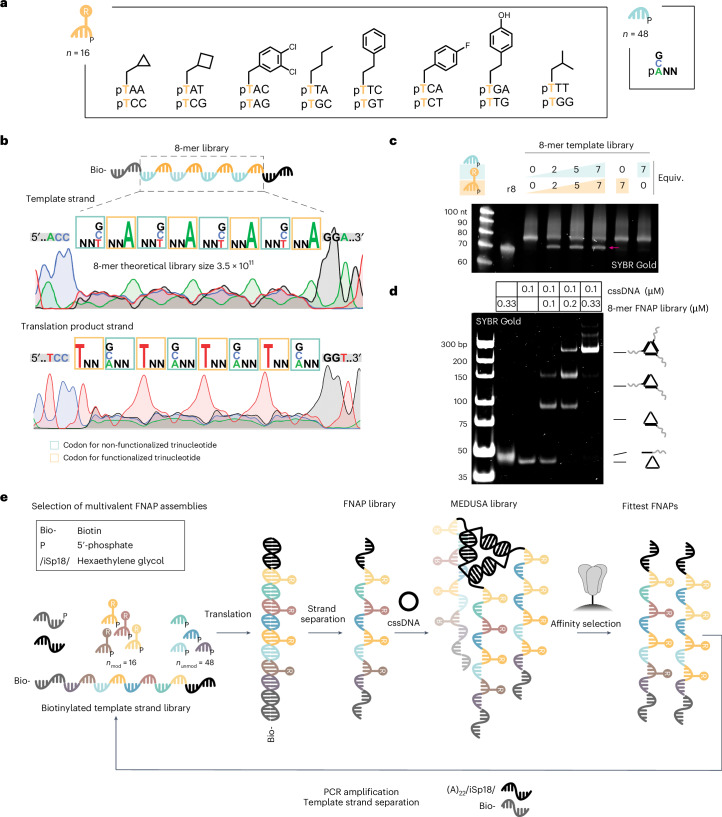
Design and synthesis of the FNAP library and MEDUSA assembly. **a**, Structures of functionalized and non-functionalized trinucleotide building blocks for the production of the FNAP library. **b**, The structure of the 8-mer template library with the corresponding Sanger sequencing electrophoretic traces for direct and reverse sequencing runs. **c**, Translation reaction of the biotinylated 8-mer template library into the corresponding FNAP library performed at different trinucleotide library concentrations. Non-biotinylated 8-mer library (r8) was loaded as a reference. The FNAP translation product is arrowed. **d**, Native PAGE analysis of the assembly of MEDUSA using a synthesized FNAP library. **e**, Scheme of the multivalent selection cycle for trimeric FNAP assemblies. Source data

We constructed trinucleotide libraries of 16 side-chain-functionalized trinucleotides encoding 8 different side chains and 48 non-functionalized trinucleotides. Based on previous reports^44,45^, we limited side-chain diversity to hydrophobic residues, including those that mimic side chains of proteinogenic amino acids and those not found in natural proteins (Fig. 2a, Supplementary Figs. 5 and 6, and Supplementary Table 1). The pertinent template library features an architecture that intersperses low-sequence diversity and side-chain-functionalized trinucleotide regions with high-diversity, non-functionalized trinucleotide regions. The former expands the chemical diversity, while the latter enhances the polymer’s conformational diversity (Fig. 2b). To minimize interference between binding units within MEDUSA, the library length was kept shorter than typical SELEX libraries (40 nt) and the average length of reported spike-binding aptamers (Supplementary Fig. 7a). To estimate the translation efficiency, we prepared corresponding biotinylated template libraries of 8-mer and 12-mer lengths (Supplementary Fig. 7b) and observed a plateau already at 2 equiv. of trinucleotide libraries relative to the template. The maximum translation yield was reached at ~5 equiv., resulting in ~70% for the 8-mer library (Fig. 2c) and ~30% for the 12-mer library, as confirmed by denaturing polyacrylamide gel electrophoresis (PAGE) (Supplementary Fig. 7c,d). Both synthetic FNAP libraries anneal efficiently with the cssDNA scaffold, yielding the trivalent combinatorial MEDUSA library (Fig. 2d, and Supplementary Fig. 7e,f). Due to its higher translation yields and to minimize cross-hybridization between longer binding units, we opted to use the 8-mer library for affinity selections.

Each round of selection commences with ligase-mediated translation of the biotinylated template DNA library into a FNAP library using 5′-phosphorylated trinucleotide building blocks. During this process, the ligase catalyses the templated synthesis of the FNAP region between the initiation and termination primers. Following strand separation and purification, the FNAP library is trimerized using the target-tailored cssDNA scaffold and subjected to affinity selection against the target protein, which is immobilized on magnetic beads. For the next selection round, the library is heat eluted and FNAPs are reverse translated back into the DNA template library (Fig. 2e). Due to the target-tailored geometric organization, the selection pressure is directed towards the enrichment of synergistic multivalent binders. Sequences capable of cooperative binding to the target should expand faster due to their slower dissociation rates. With every round, affinity selection based on multivalent interactions becomes more dominant.

### Multivalent affinity selections yield a high degree of sequence diversity

**Fig. 3:**
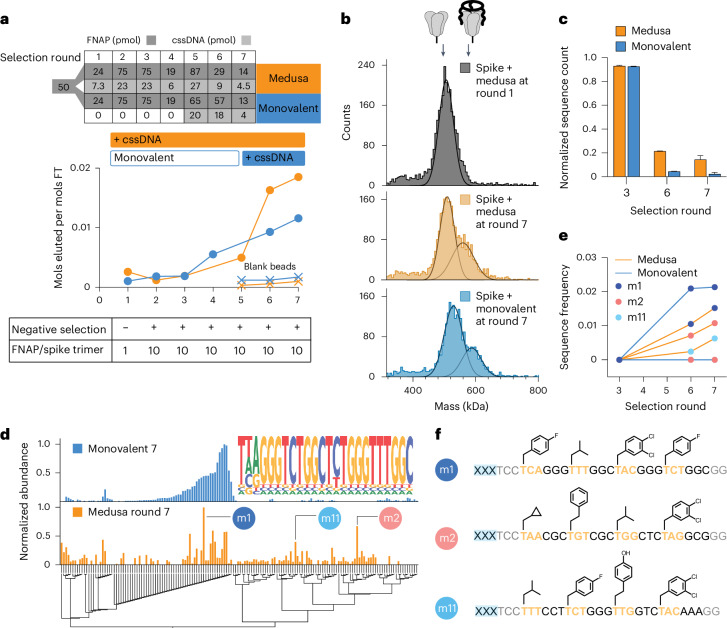
Selection of spike-protein-binding FNAPs via multivalent SELEX. **a**, Progress in spike-binding selection for multivalent (medusa) and monovalently prefocused (monovalent) selection strategies. Bulk affinity of trivalent and monovalent FNAP libraries to trimeric spike protein was assessed by quantifying the amount of FNAPs in the flow-through (FT) versus elution fractions. **b**, Increase in bulk affinity of trimeric FNAP libraries to spike protein by comparing the MEDUSA assemblies prepared with the FNAP library from selection round 1 versus selection round 7 for both selection strategies using mass photometry. **c**, Progression of selection process indicated by the decrease in FNAP library complexity using NGS. Data are presented as mean ± s.d. (*n* = 2, independent sequencing runs). **d**, Multiple sequence alignment of the top sequences from NGS data for two tested selection strategies with corresponding sequence abundances. The common motif of sequences retrieved from the monovalent selection process is displayed as a nested graph. **e**, Enrichment of three selected hits over the rounds of selection for two tested selection strategies using NGS. **f**, Sequences and side-chain structures of selected FNAPs. xxx, scaffold-hybridization region. Source data

As in SELEX, the frequency of high-affinity sequences in the naïve FNAP library is low, with some binders present in only a single copy. Moreover, the distribution of binding affinities is unknown. To address the high complexity of the naïve library, statistically unfavourable for the stochastic assembly of MEDUSAs during early rounds of selection, two selection strategies were tested: (1) entirely multivalent and (2) a monovalent pre-enrichment during initial rounds, followed by multivalent final selections. We conducted two parallel selection campaigns against the SARS-CoV-2 spike protein (Supplementary Fig. 8). The PAGE-purified starting FNAP library was split and one half (termed ‘monovalent’) first underwent four rounds of monovalent affinity selections before multimerization. The other half was directly subjected to multivalent selections from round 1 (termed ‘medusa’, Fig. 3a, top). The ratio between the FNAP library and target was maintained constant for both strategies throughout the selection campaign (Fig. 3a, bottom). After seven rounds of affinity selection, the binding capacity of both the monovalent and MEDUSA libraries began to plateau, prompting us to conclude the selection campaign (Fig. 3a). The presence of binding sequences in both libraries after selection round 7 was confirmed by mass photometry (Fig. 3b).

Sequencing data at rounds 3, 6 and 7 revealed a steady decrease in the number of unique reads in the sequence pools as selection progressed, indicating the expansion of binding sequences for both selection strategies. The diversity of the final MEDUSA library was around 4.6 times higher than the monovalent library, as estimated from the number of unique sequences in the pool (Fig. 3c and Supplementary Fig. 9a,b). We reason two potential processes could contribute to the difference in the complexities of the libraries: enrichment of synergistically binding sequences that otherwise cannot expand in the monovalent selection regime, and ‘parasitic’ carry-over of low-affinity sequences into the late rounds of selection due to their stochastic incorporation into the MEDUSAs composed of high-affinity sequences. As ‘parasitic’ low-affinity sequences effectively render respective MEDUSAs less active than the assemblies with higher numbers of cooperatively binding sequences, we reason that the selection is directed towards the decrease in the frequencies of low-affinity sequences.

Next-generation sequencing (NGS) data after the final round of selection revealed that the sequence pool for monovalent selection strategy was eventually dominated by one family of FNAPs featuring a common consensus sequence (Fig. 3d, top). Notably, the discovered motif can also be found in several reported DNA aptamers, thereby further validating the specific enrichment of binding sequences^46^ (Supplementary Fig. 10). In contrast, the sequencing data obtained from the medusa library display a more diverse range of sequences in addition to the aforementioned sequence family (Fig. 3d, bottom). The differences in sequence compositions between the two libraries are also evident in the changes in side-chain frequencies observed across different rounds of selection (Supplementary Fig. 11). To validate the enriched FNAPs, we selected the three most abundant and sequence-unrelated sequences based on the Levenshtein distances matrix (Supplementary Fig. 12). The sequence m1 represents the family of sequences enriched through monovalent selection, while m2 and m11 are the sequences that emerged uniquely through the multivalent, geometry-constrained selection process (Fig. 3e,f).

### Evolution with MEDUSA yields unique target selectivity

**Fig. 4:**
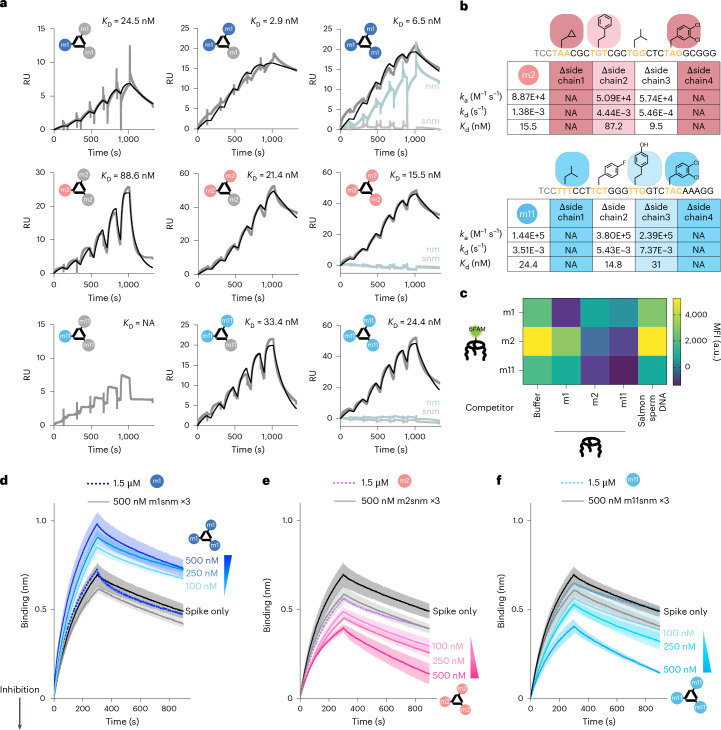
Affinity characterization of MEDUSAs prepared with selected FNAPs. **a**, SPR sensorgrams characterizing the binding kinetics between trimeric SARS-CoV-2 spike protein immobilized on the CM3 chip and mono-, di- and trivalent assemblies of selected FNAPs. RU, response units. As controls, assemblies prepared with non-modified (nm) and scrambled non-modified (snm) versions of the corresponding binding units were used. The concentrations of injected assemblies were 9, 18, 37, 75, 150 and 300 nM. The black curves represent the binding kinetics fit. **b**, SPR kinetic parameters (*k*_a_, association rate constant; *k*_d_, dissociation rate constant; *K*_d_, dissociation constant) for trivalent supramolecular assemblies prepared using side-chain-deficient variants of m2 and m11 sequences. **c**, Competition ELISA assay indicates distinct binding specificity between FNAPs selected via multivalent and monovalent selection strategies. The assay was performed in duplicate (*n* = 2, technical replicates), and the mean values are plotted. **d**–**f**, Competition BLI sensorgrams depicting spike protein binding to dimeric ACE2-Fc, immobilized on the Protein A BLI probes. Assemblies of selected FNAPs (**d**, m1 MEDUSA; **e**, m2 MEDUSA, **f**, m11 MEDUSA) were mixed with trimeric SARS-CoV-2 spike protein at three increasing assembly concentrations. The decrease in mass transfer to the BLI probe indicates that the compound interferes with the ACE2–spike protein interaction. Assemblies of scrambled non-modified variants of the selected sequences were used as negative controls. The gradient triangle indicates the increasing concentration of FNAP assembly. All measurements were performed in duplicate (*n* = 2, technical replicates), and average signals were plotted with the s.d. range highlighted. NA, not applicable. Source data

To confirm binding affinity for spike protein, the monovalent binding of the full-length and primer-minimized versions of selected FNAPs (Supplementary Fig. 13a) was tested via surface plasmon resonance (SPR) analysis. The m1 sequence displayed low-nanomolar monovalent affinity for the spike protein (Supplementary Fig. 13b), and m2 showed ~200-nM affinity (Supplementary Fig. 13c). No SPR signal was observed for monovalent m11, even at the highest analyte concentration tested (600 nM). We found that the primer region did not significantly impact binding performance (Supplementary Fig. 13d,e), allowing the use of primer-minimized variants for further experiments due to higher yields (Supplementary Fig. 14). We prepared mono-, di- and trivalent MEDUSAs using the cssDNA multimerization scaffold featuring three orthogonal FNAP-binding sites (cssDNAort), and we also prepared scrambled and scrambled non-modified control variants. SPR sensorgrams revealed that multimerization of m1 sequence did not cause substantial avidity increase in comparison to monovalent m1, suggesting a poor binding cooperativity. In contrast, the increase in the number of m2 and m11 binding units led to an ∼10-fold increase in binding strength for m2, and an increase from undetectable to 24 nM for m11 (comparing monovalent and trivalent structures) (Fig. 4a). This difference in response upon multimerizations suggests that m2 and m11 operate with different binding dynamics, showing behaviour similar to chelation for m2 and cooperative binding for m11 (ref. ^47^). Using cssDNAort enabled assessment of all seven possible hetero-FNAP multivalent MEDUSAs (Supplementary Fig. 15a), all showing comparable nanomolar avidities, with no clearly optimal combination (Supplementary Fig. 15b,c). Notably, trivalent assemblies demonstrated relatively slow association and dissociation rates, which may suggest conformational changes are required before binding (Supplementary Table 7), a phenomenon often reported for aptamer–protein interactions^48^.

Monovalently derived m1 and multivalently derived m2 and m11 sequences displayed dramatically different reliance on side-chain modifications for binding to the spike protein. Stripping all modifications from the m1 sequence while preserving its DNA sequence (Fig. 4a, ‘nm’) did not result in any substantial alteration of the binding affinity. In contrast, removal of modifications from the multivalently selected m2 and m11 severely disrupted binding. Individual side-chain contributions in m2 and m11 were measured by preparing a systematic set of side-chain-deficient variants and measuring their affinities (Fig. 4b, and Supplementary Fig. 16). Three out of four side chains were critical for target binding, a significantly larger portion compared to reported binders with higher functionalization density^38^. Additionally, NUPACK simulations predict secondary structures, highlighting the consequences of high-diversity regions in the selection of binding sequences (Supplementary Figs. 17–19).

Following the evidence that multivalent selection yields cooperative binders, we were curious to investigate target selectivity in the context of mutations. Repeating the SPR assays with m1, m2 and m11 MEDUSAs to Omicron BA.4, XBB1.16.1 and Delta B1.167.2 mutants showed orthogonal selectivity for m1 versus m2 and m11 (Supplementary Fig. 20). The m1 MEDUSAs were able to bind all mutants, hinting toward a lack of true target selectivity but a general binding interface. However, the m2 and m11 MEDUSAs exclusively bound to the wild-type spike, the target they were selected for, indicating that these binders not only benefit from multivalent cooperativity but also show remarkable target selectivity. We deduce that m1 binds to a different physical section of the spike protein compared to m2 and m11, and assessed the binding specificities of the MEDUSAs via competition ELISA assays. A significant decrease in the amount of bound assemblies was observed for MEDUSAs prepared with identical FNAP-binding units. However, no competition was observed between m1 MEDUSA and MEDUSAs featuring m2 and m11, indicating that geometry-constrained selection indeed reveals sequences targeting an orthogonal epitope compared to monovalent SELEX. Furthermore, binding epitopes of multivalently evolved sequences m2 and m11 were found to overlap, as evidenced by reduced FAM fluorescence in the m2–m11 MEDUSA pair (Fig. 4c, and Supplementary Fig. 21). No significant competition was observed between the selected MEDUSAs and a subset of reported aptamers with RBD and NTD binding specificities^11^, suggesting distinct epitopes (Supplementary Fig. 22).

To determine whether the observed binding specificities correlated with functional performance differences, we tested their ability to interrogate PPIs between spike and dimeric ACE2 in a competitive biolayer interferometry (BLI) assay. MEDUSAs displaying m1, m2 and m11 FNAPs were prepared and preincubated with the spike protein. If MEDUSAs interfere with the spike protein–ACE2 interaction, immersing ACE2-functionalized probes into solutions of spike protein would result in a decrease in mass transfer to the probe. However, the opposite was observed with m1 MEDUSA, which showed a concentration-dependent increase in mass transfer to the ACE2-coated BLI probe. This suggests that m1 MEDUSA binding does not create steric hindrance for ACE2 but instead allows the entire MEDUSA–spike protein complex to bind the probe, increasing mass transfer relative to the spike protein alone. In contrast, for m2 and m11 MEDUSAs, a marked drop in the BLI signal was observed, indicating inhibition of ACE2 binding. Notably, this repressive effect was observed only with trivalent assemblies and not with the binding units alone, underscoring the importance of multivalency for the functional performance of multivalently selected sequences (Fig. 4d–f).

### Scaffold geometry and rigidity interplay for functional activity

**Fig. 5:**
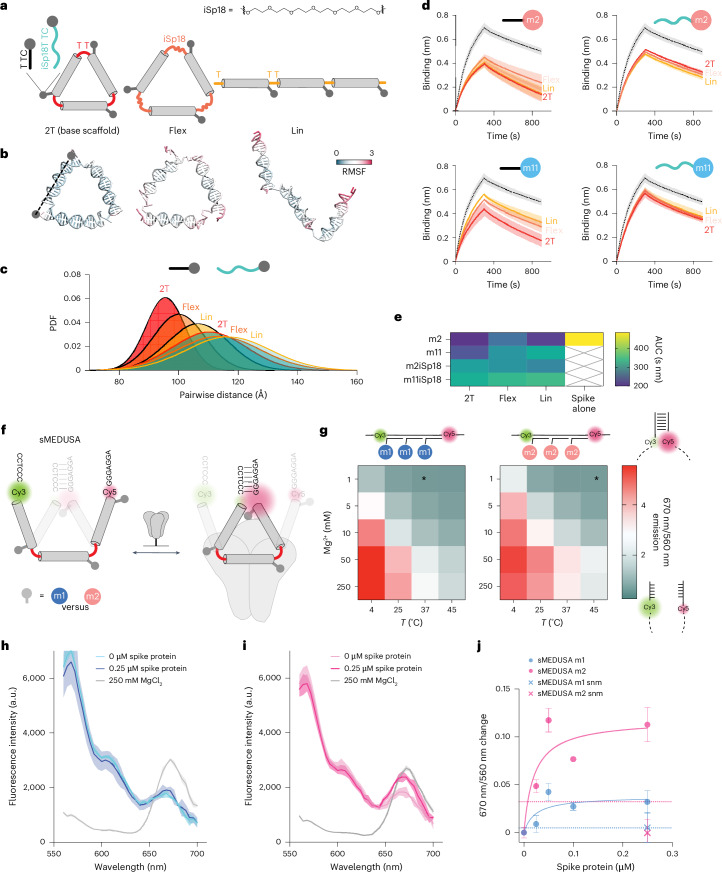
Assessment of the effect of the scaffold configuration on the performance of the FNAP assemblies. **a**, A diagram depicting the base multimerization scaffold (2T), its linear (Lin) variant and a variant with more flexible vertex regions achieved by substituting one of the T nucleotides with a hexaethylene glycol spacer (Flex). FNAPs with extended spacers separate the binding region from the assembly’s core. Corresponding coarse-grained models of the scaffold structures are given below. **b**, Close-to-average oxDNA models of the designed scaffolds. RMSF, root mean square fluctuation. **c**, Distribution of end-to-end distances between FNAP attachment points for various assemblies, based on coarse-grained simulations. The average distribution of all three distances is plotted for each particle. **d**, Competition BLI sensorgrams for FNAP assemblies with various scaffold and binding unit compositions; spike protein without assemblies is plotted with a dashed line. All measurements were performed in duplicate (*n* = 2, technical replicates), and average signals are plotted with s.d. ranges highlighted. **e**, A heatmap summarizing the mean areas under the curve (AUC) calculated from the BLI data for each scaffold and FNAP combination. **f**, Schematic of sMEDUSA, which features two conformational states in dynamic equilibrium, with the closed conformation being stabilized upon multivalent *cis*-interaction with the spike protein (shown in grey). **g**, Conformational state diagram for m1 and m2 devices as a function of temperature and Mg^2+^ concentration. The condition used in the spike protein binding assays is indicated by an asterisk. **h**, Fluorescence intensity spectral scans for m1 sMEDUSA. **i**, Fluorescence intensity spectral scans for m2 sMEDUSA. The respective spectral scans of sMEDUSAs in the presence of 250 mM MgCl_2_ are included as a reference for maximal FRET. **j**, Change in 670 nm/560 nm fluorescence ratio relative to sMEDUSA in buffer for m1 and m2 sMEDUSAs in the presence of increasing concentrations of spike protein, and for scrambled non-modified (snm) sMEDUSAs at 0.25 μM spike concentration. Dashed lines represent 670 nm/560 nm ratio change for m1 and m2 sMEDUSAs in the presence of 100 μg ml^−1^ BSA. All FRET assays were performed in triplicate (*n* = 3, technical replicates), with the average values plotted and s.d. ranges highlighted. Source data

We produced a set of alternative structures with increased conformational flexibility (Fig. 5a, top) to explore how MEDUSA scaffold and linker configurations impact performance. The original cssDNA scaffold (2T) was linearized (Lin) to explore geometry and we replaced one of the T nucleotides by hexaethylene glycol (iSp18) in each of the three vertices to explore flexibility (Flex) (Fig. 5a). Increased flexibility of these alternative MEDUSAs was confirmed with OxDNA simulations^49^ (Fig. 5b,c and Supplementary Fig. 23). Local binding unit flexibility was modified by placing iSP18 linkers between the m2 and m11 binding units and their scaffold-hybridization regions. The inhibitory potential of all new scaffold and linker versions (Supplementary Fig. 24) was tested in a BLI competition assay with ACE2 dimer. Our results revealed that modifying the original geometry and flexibility of ligand presentation diminishes the performance of m2 and m11 assemblies. The most notable decrease was observed for assemblies with excessive local linker flexibility (Fig. 5d,e), regardless of the scaffold variant. The increase in the assembly’s core flexibility correlated with the decrease of the assembly’s inhibitory potential, which was especially prominent for the assemblies of the m11 binding unit, which was shown to gain most from multivalent presentation. We conclude that conformationally constrained scaffolds with target-specific geometry are crucial for the inhibitory potential of MEDUSA.

In an attempt to translate this optimal geometric binding and target selectivity into biotechnology tools, we explored if multivalent target-induced sensors could be generated from MEDUSAs. The conformation that facilitates the most effective ligand positioning should be favoured upon the multivalent engagement of its binding units with a multimeric target. Crucially, this only holds true if the binding units can engage in synergistic binding in *cis*. We devised a switchable MEDUSA variant (sMEDUSA) that can adopt two discrete conformational states: a suboptimal open state for multivalent *cis*-binding, and a more optimal conformationally constrained closed state. In the open state, sMEDUSA resembles the Lin structure, while in the closed state it is akin to the original MEDUSA. The structural basis for the switchable behaviour of sMEDUSA arises from its scaffold, which contains short (6-nt) complementary regions at the 5′ and 3′ termini flanking three FNAP-binding sites (Supplementary Fig. 25). Upon the hybridization of this scaffold with three binding units, a molecular switchable system with dynamic equilibrium between open and closed states is obtained. By introducing a Förster resonance energy transfer (FRET) pair at the root of the dynamic hairpin, the proportion of closed-state molecules can be monitored in bulk by ratiometric readout of fluorescence intensities at 670 nm (indicative of the closed conformation) and 560 nm (indicative of the open conformation) (Fig. 5f). We successfully validated the system by modulating the equilibrium between open and closed states using temperature and Mg^2+^ concentration: both a decrease in temperature and an increase in Mg^2+^ concentration led to the stabilization of the hairpin and a subsequent increase in the closed-state population (Fig. 5g).

To explore the dynamic range of the system and to test whether the same shift in the equilibrium between the open and closed conformations of sMEDUSA could be achieved by cooperative multivalent binding in *cis*, we conducted a sandbox experiment with sMEDUSAs featuring short DNA sequences with different GC content in place of FNAP-binding units (Supplementary Fig. 26). We observed that the conformationally constrained 2T MEDUSA target was the most effective in mediating the FRET increase of the sMEDUSAs, and the increase in GC content of the binding units correlated with the increase in FRET (Supplementary Fig. 26e). Next, we tested whether the conformational dynamics of sMEDUSA could be altered by the spike protein (Supplementary Fig. 27). We compared FRET efficiencies of m1 and m2 sMEDUSAs, along with sMEDUSAs with scrambled non-modified control sequences. Excitingly, the addition of the protein to the m2 sMEDUSA led to a more substantial increase in FRET compared with m1 sMEDUSA (Fig. 5h–j), indicating effective multivalent *cis*-binding to the spike protein and consequently a shift of the conformational state equilibrium towards the closed conformation. These data lay the foundation for the potential of the sMEDUSA system in molecular sensing applications as well as non-FRET-based sensor technologies.

## Conclusion

In this study, we established a simple method to generate new multivalent binders that take advantage of the multimeric nature of many clinically relevant proteins. Rather than evolution of the fittest monovalent binder to an oligomeric target and postselection multimerization, we implement a priori a target-tailored multimerization step to direct selection towards the most cooperative multivalent binders. Through simulations, we rationalized the concept of multivalent selections with free recombination, showing that multimerization of a random binding unit library alters selection outcomes compared to monovalent selection with the same library. By incorporating target valency and geometry into the selection process, we generated binders with unique structures and specificities. Interestingly, the functional contribution of side-chain modifications was minimal for sequences obtained through monovalent selection, yet critical for the MEDUSA hits in the first and last codon position. We then demonstrated the utility of assemblies of multivalently selected binders for interrogation of PPIs, by comparing them to the sequence enriched through monovalent selection. Only the evolved MEDUSA binders displayed potent inhibition of spike protein/ACE2, and moreover they were highly selective for the target toward which they were evolved. We hypothesize this mutant selectivity to be strongly correlated with the presence of side-chain functionalities. Scaffold configuration strongly affects the functional performance of MEDUSAs, and conformational switching made it possible to engineer a proof-of-concept FRET sensor for spike detection. This opens avenues to develop selective diagnostics toward pathogens and observation of mutations, although the timeline for selection of binders in an active pandemic will be limited to obtaining sufficient amounts of the new target. As the current FNAP library strongly contributes to hydrophobic-mediated binding, expansion of the chemical sequence space is envisioned to cover different binding modes, a focus point for next generations of MEDUSA. Overall, MEDUSA leverages the natural paradigm of molecular coevolution within multivalent assemblies to create a simple and tunable platform for discovering multivalent nucleic-acid-based binders that cannot be obtained through monovalent SELEX and presents a comprehensive workflow toward enlarging the current aptamer binder and drug discovery space.

## Methods

### Template library preparation and characterization

The template library for ligase-mediated translation was synthesized using standard phosphoramidite synthesis on a 200-nmol scale. Two mixtures of phosphoramidites were prepared in dry acetonitrile at a 200 mM total concentration with the following molar ratios of bases:

Mixture 4 = DMT-dA(Bz):DMT-dT:DMT-dG(dmf):DMT-dC(Ac) = 1.5:1:1.15:1.25

Mixture 5 = DMT-dA(Bz):DMT-dT:DMT-dG(dmf):DMT-dC(Ac) = 0:1:1.15:1.25

The template 8-mer library sequence is as follows:

CCATAGACTAGCAACTTTCACC44544A44544A44544A44544AGGAGTGATGTAGGTGGTAGAGGAA

The template 12-mer library sequence is as follows:

CCATAGACTAGCAACTTTCACC44544A44544A44544A44544A44544A44544AGGAGTGATGTAGGTGGTAGAGGAA

The template library was synthesized DMT-off, and cleaved/deprotected with a 40% aqueous solution of methylamine at 65 °C for 1 h. The library was eluted from the column with 300 μl of 30% acetonitrile, and the crude library was precipitated by adding 1/10 vol. of 3 M sodium acetate and 3 vols. of absolute ethanol. The crude library was pelleted by incubating the solution at −20 °C for 20 min and centrifugation at 15,000*g*, 4 °C for 30 min. The pellet was then reconstituted in 300 μl of nuclease-free water, and the concentration was measured using absorbance at 260 nm. Finally, 200 μg of crude DNA was purified using 10% polyacrylamide gel with 8 M urea.

### Circular ssDNA scaffold strand production

Circular ssDNA was prepared by stepwise addition of linear 5′-phosphorylated DNA (l-DNA) and the splint strand into the T4 DNA ligase mixture at low Mg(II) concentrations^50^. The initial reactions were set up in a 40-µl volume with 2 µM l-DNA, 30 µM splint strand and 0.05× T4 ligase buffer. After adding 20 U of T4 DNA ligase (Fisher Scientific, EL0012), the reaction was incubated at 22 °C. New portions of l-DNA and splint strand were added to the reaction mixture at 30-min intervals according to the following order: addition 1: 4 μl of 20 μM I-DNA; addition 2: 4 μl of 20 μM I-DNA and 3 μl of 200 μM splint; addition 3: 6 μl of 0.5× T4 DNA ligase buffer; addition 4: 4 μl of 20 μM I-DNA and 3 μl of 200 μM splint; addition 5: 3 μl of 20 μM I-DNA, 3 μl of 200 μM splint, and 2 μl of 0.5× T4 DNA ligase buffer; addition 6: 3 μl of 20 μM I-DNA.

After the last addition, the reaction was left overnight at 22 °C. Then, 2 μl of Exonuclease I (NEB, catalogue number M0293L) was added to 75 μl of the reaction mixture, and the reaction was incubated at 37 °C for 1 h. Exonuclease I was heat-inactivated at 80 °C for 15 min. For scaled-up production, eight reactions were performed in parallel in PCR strips. These reactions were later combined into a 1.5-ml tube, and the volume was reduced to approximately 200 μl using a vacuum concentrator (SpeedVac DNA130) at 65 °C for 60 min. Next, DNA precipitation was carried out by adding 1/10 vol. of 3 M sodium acetate and 3 vols. of absolute ethanol. The mixture was then incubated at −20 °C for 30 min and centrifuged at 15,000*g*, 4 °C for 30 min. Subsequently, the pellet obtained from the precipitation was resuspended in a solution containing 30 μl of RF water, 30 μl of 8 M urea, 60% glycerol and 1× TBE loading buffer. This mixture was heated to 90 °C for 5 min before loading onto the preparative PAGE gel (Supplementary Fig. 3b).

### Biotinylated template library preparation

For production of the biotinylated ssDNA template library, the eluted FNAP library from the previous selection round was preamplified in eight 25-μl Q5 DNA polymerase (NEB, catalogue number M0491S) PCR reactions using a subsaturation number of cycles according to qPCR. The amplicons were then purified using a Monarch PCR & DNA Cleanup Kit (NEB, catalogue number T1030) following manufacturer’s protocol. The concentration of the library was measured using Nanodrop, and preparative PCR was performed on 64 25-μl Q5 DNA polymerase PCR reactions using the subsaturation number of cycles according to qPCR (typical matrix input was 0.3 pmol per reaction, which were amplified in six PCR cycles). The following primers were used: scaffold primer: AAA AAA AAA AAA AAA AAA /iSp18/TT CCT CTA CCA CCT ACA TCA C; template primer: /52-Bio//iSp18/CCATAGACTAGCAACTTTCACC. The reaction mixtures were placed in a thermocycler preheated to 98 °C and amplified using the following programme: 98 °C 1 min, six cycles of (98 °C, 10 s; 61 °C, 30 s, 72 °C, 15 s), 4 °C hold. After the thermocycle programme is complete, reactions were pooled into four 2-ml tubes (400 μl per tube) and amplicons were ethanol-precipitated as described previously. The pellets were resuspended in 10 μl of RF water and 10 μl of 2× Novex TBE-Urea Sample Buffer (Invitrogen, catalogue number LC6876) per pellet yielding a total volume of 80 μl. The amplicons were denatured at 95 ^o^C for 5 min and loaded onto 10% polyacrylamide gel with 8 M urea (10 μl per well of 1.5-mm gel). The gel was run at 190 V for 1.5 h and the bands were visualized using ultraviolet-shadowing against a thin-layer chromatography plate. The lower-molecular-weight band that corresponds to the biotinylated template was excised and extracted.

### FNAP production via T3 DNA ligase-mediated translation

The purified biotinylated library was translated into a functionalized nucleic acid polymer library using T3 DNA ligase. For each 10-μl reaction, the following components were mixed in a PCR strip: 1 μl of 10× T4 RNA ligase reaction buffer (NEB, B0216S), 0.75 μl of 20 μM initiation (1.5 equiv.) and phosphorylated termination primers (1.5 equiv.), 0.64 μl or 0.77 μl of 5 mM functionalized trinucleotide library mix (5 equiv. or 6 equiv. per occurrence of the corresponding codon for the 8-mer and 12-mer library, respectively), 1.8 μl or 2.13 μl of 5.4 mM non-functionalized trinucleotide library mix (5 equiv. or 6 equiv. per occurrence of the corresponding codon for the 8-mer and 12-mer library, respectively), and 10 pmol of biotinylated template library. The mixture was subjected to the following thermocycler programme: 95 °C for 10 s, 65 °C for 4 min, 65 °C to 4 °C at −0.1 °C per cycle (610 cycles).

After annealing, 1.2 μl of 10 mM ATP and 0.6 μl of T3 DNA ligase were added, and reactions were incubated at 4 °C for 12 h and then for 2 h at 16 °C. Alkaline strand separation was performed using Dynabeads MyOne Streptavidin C1 magnetic beads (ThermoFisher). One microlitre of 10 μg μl^−1^ stock bead suspension was used per 4 pmol of biotinylated template. The beads were washed three times with 1 vol. of 1× Binding and Washing (B&W) Buffer (5 mM Tris–HCl, pH 7.5, 0.5 mM EDTA, 1 M NaCl) and suspended in 1 vol. of 2× B&W Buffer (10 mM Tris–HCl, pH 7.5, 1 mM EDTA, 2 M NaCl). The translation reactions were then added to the bead suspension and incubated at room temperature for 1.5 h in a rotary mixer. The supernatant was removed by magnetic separation, and the beads were washed three times with 1× B&W Buffer (combined volume of translation mixture + original volume of 1% bead suspension) with a 5-min incubation in the rotary mixer followed by 1-min magnetic separation. After the last wash, the beads were resuspended in freshly prepared 20 mM NaOH (original volume of 1% bead suspension), and the FNAP library strand was eluted for 5 min in a rotary mixer. The elution was repeated a second time, the fractions were pooled, and 1/40 vol of 1 M HEPES (pH 7.3) was added to neutralize the base. The FNAP strands were purified using the Monarch PCR & DNA Cleanup Kit (NEB, catalogue number T1030) by adding 2 vols of Cleanup Binding Buffer and 6 vols of absolute ethanol. The column was then washed once with 250 μl of Wash buffer, and the FNAP strand was eluted with 10–20 μl of RF water and PAGE-purified as described previously.

### MEDUSA folding

Trivalent assemblies of selected FNAPs were prepared by annealing PAGE-purified FNAP strands with multimerization scaffolds at a 3.3:1 molar ratio in 1× DPBS,1 mM MgCl_2_, using the thermocycler programme: 95 °C for 2 min, 80 °C to 20 °C at −2 °C per cycle (30 cycles). Assembly folding was confirmed via 6% native PAGE (Fig. S24).

### SPR assays

All SPR assays were performed at 25 °C on the Biacore 8K+ system. Approximately 1,000 RU of trimeric spike protein was immobilized on the CM3 chip (Cytiva, BR-1005-36) using the EDC/NHS kit (Cytiva, BR100050). For this, a 10 µg ml^−1^ spike-protein solution in 10 mM sodium acetate buffer pH 5.5 was used with a contact time of 290 s and a flow rate of 5 µl min^−1^. Single-cycle kinetics runs were performed using 120 s for association and 300 s for dissociation phases at 15 µl min^−1^ flow rate. 1× DPBS supplemented with 1 mM MgCl_2_ was used as the running buffer. To ensure that our SPR programme allows for adequate measurement of association rates, we conducted test runs at increasing flow rates. No significant difference in *k*_on_ was observed between 15 µl min^−1^ and 50 µl min^−1^ flow rates, confirming that the binding curves are not mass-transport limited.

### Competition ELISA assay

Wells of Greiner microplates (96-well, polystyrene, half-area, white, high binding (675074); 96-well, polystyrene, half-area, black, high-binding plate (675077)) were coated with 30 µl of 20 µg ml^−1^ spike protein trimer solutions in 1× PBS at 4 °C overnight. The coating solution was discarded by decanting the plate, and the wells were washed three times with 100 µl of 1× DPBS, 1 mM MgCl_2_, 0.1 mg ml^−1^ BSA, 0.005% Tween 20. The wells were then blocked with 30 µl of 1% BSA in 1× DPBS for 2 h at 4 °C. Afterwards, the blocking solution was discarded by decanting the plate. To the blocked plate, 25 µl of 1,000-nM unlabelled assembly was added followed by 25 µl of 100-nM fluorescein-labelled assembly in 1× DPBS, 1 mM MgCl_2_. As a negative control, an equivalent amount of salmon sperm DNA was added in place of unlabelled assembly. The plate was centrifuged at 2,000 rpm for 2 min and incubated for 1 h at room temperature. The solutions were removed by decanting the plate and the wells were washed three times by putting 100 µl of 1× DPBS, 1 mM MgCl_2_, 0.1 mg ml^−1^ BSA, 0.005% Tween 20 in the well for 5 s, and removing by the multichannel. After the last wash, the plate was tapped firmly on a paper towel to remove any residual wash buffer. Then, 50 µl of RF MiQ was added, the plate was centrifuged at 2000 rpm for 2 min and placed into a preheated oven at 90 °C for 5 min. The plate then was cooled on ice for 5 min, centrifuged and imaged with extended gain and a 6.25-mm read height (BioTek Cytation 5 Plate Imager).

### Competition BLI assays

All experiments were performed at 25 ^o^C on a Gator BLI system. The running buffer was 1× DPBS supplemented with 1 mM MgCl_2_. For competition assays, dimeric ACE2-Fc was diluted to 20 µg ml^−1^ and captured on the Protein A probes (GatorBio, PL168-160001) using a loading time of 600 s. After loading and 200 s of equilibration, loaded probes were dipped into a solution of premixed spike protein at 50 nM with the MEDUSA or monomeric FNAP. The highest tested concentrations for trivalent MEDUSA and monovalent FNAP were 500 nM and 1,500 nM, respectively. The association phase duration was 300 s followed by 600 s of dissociation phase.

### Electron microscopy specimen preparation and data acquisition

#### Specimen preparation

MEDUSA complexes were prepared by annealing the scaffold strand with selected binding units at 3 μM concentration in 1× HBS (20 mM HEPES pH 7.3, 150 mM NaCl) for cryo-electron microscopy (cryo-EM) experiments. The ssDNA 2T scaffold strand (Supplementary Table 4) and binding unit m2 P1 (Supplementary Table 5) were used to prepare the assemblies shown in Fig. 4f. Grids were prepared by glow-discharging in a GloQube Plus device (Quorum Technologies) using a 15-mA current and a 90-s glow time.

A total of 3.5 µl of MEDUSA complex was applied to Quantifoil copper 400-mesh R1.2/1.3 grids (JenaBioscience) and subsequently flash-frozen in liquid ethane using a Vitrobot Mark IV (ThermoFisher Scientific). The Vitrobot chamber was maintained at 10 °C with 95% humidity. After the sample application, the grid was allowed to rest for 10 s before blotting with a force of 10 (Vitrobot arbitrary units) for 4.0 s.

#### Data acquisition

Imaging of the MEDUSA complexes was performed using a Glacios transmission electron microscope (200 keV, X-FEG; ThermoFisher Scientific) equipped with a Falcon IV direct electron detector (Dubochet Center of Imaging). Data were acquired at a nominal magnification of 150,000×, corresponding to a pixel size of 0.93 Å. A total dose of 50 electrons per Å^2^ was applied, and 1,196 micrographs were recorded in EER format. Automated data collection was done using EPU v.3.6 software (ThermoFisher Scientific), with defocus values ranging from −1.0 µm to −2.4 µm.

#### Cryo-EM image processing, model building and refinement

Subsequent image-processing steps were performed using CryoSPARC v.4.3^51^. Motion correction was applied using the patch-based motion-correction algorithm (CryoSPARC Live implementation) to correct for stage drift and anisotropic motion, followed by dose-weighting of the micrographs. Contrast transfer function parameters were estimated using the Patch contrast transfer function estimation tool. Particle picking, 2D and 3D classification, and refinement were also conducted within CryoSPARC. The final resolution was determined after performing ab initio modelling, which was sufficient for comparison with available reference structures. No further refinements were applied beyond visualization purposes. Structural representations and figures were generated using UCSF ChimeraX^52^.

### FRET assays

The sMEDUSAs were folded in 1× DPBS with 1 mM MgCl_2_ at a concentration of 0.2 µM. Upon completion of the thermocycler programme, a working solution of 0.1 µM assemblies was prepared by diluting the folding mixture twofold with RF water. Dilutions of Wuhan WT spike protein (ExcellGene) were prepared from a 1 mg ml^−1^ stock solution in 1× DPBS with 1 mM MgCl_2_. The MgCl_2_ concentration was adjusted proportionally to the volume of spike protein stock solution using a 10 mM MgCl_2_ solution. The assay was performed in a 96-well microplate (polystyrene, F-bottom with chimney well, µCLEAR, black, non-binding, Greiner Bio-One) by mixing 65 µl of 100 nM sMEDUSA with 65 µl of spike protein solutions at final concentrations of 10, 20, 40, and 100 µg ml^−1^. The mixture was incubated at room temperature for 20 min, followed by a 10-min temperature equilibration at 37 °C. Spectral scans were acquired at 37 °C using a BioTek Cytation 5 Plate Imager under the following parameters: excitation at 540/10 nm, emission range from 560/10 to 700 nm, emission step size of 4 nm, gain set to 100, optics positioned at the top, and a read height of 7 mm. The data were fitted with the ‘one-site-specific binding’ model in GraphPad Prism without imposing constraints on the *K*_d_ values.

A detailed description of other experimental protocols is provided in the Supplementary Information.

## Online content

Any methods, additional references, Nature Portfolio reporting summaries, source data, extended data, supplementary information, acknowledgements, peer review information; details of author contributions and competing interests; and statements of data and code availability are available at 10.1038/s41565-025-01939-8.

## Supplementary information

Supplementary Information Extended methods, Supplementary Figs. 1–27 and Tables 1–7.

## Source data

Source Data Figs. 1–5 Source Data Fig. 1. Numerical source data for panels b–d. Source Data Fig. 2. Full-length, unprocessed image of the gel in panels c and d. Source Data Fig. 3. Numerical source data for panels a, c–e. Source Data Fig. 4. Numerical source data for panels a–f. Source Data Fig. 5. Numerical source data for panels c–e, g–j.

## Data Availability

Extended methods and supporting figures are available in the Supplementary Information. All of the data that support the findings of this study are available via Zenodo at 10.5281/zenodo.14261819 (ref. ^53^). Cryo-EM imaging datasets for reconstruction are available from the authors upon request. Source data are provided with this paper.

## Code availability

The custom code used to analyse the data and generate figures in this study is available on GitHub. MEDUSA-NGS data-processing scripts: https://github.com/KononenkoArtem/MEDUSA-NGS-data-processing-scripts. Avidity Monte Carlo integration: https://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@github.com/VinceCar/AvidityMonteCarloIntegration.git. Multivalent selection engine: https://github.com/VinceCar/multivalent_selection_engine.
