# Supplementary material for: Evolution of multivalent supramolecular assemblies of aptamers with target-defined spatial organization
Source: Nat Nanotechnol. 2025 Jun 6;20(8):1087–97. doi: 10.1038/s41565-025-01939-8 (PMC12373506; doi:10.1038/s41565-025-01939-8)
Supplement: Supplementary file 1 — Extended methods, Supplementary Figs. 1–27 and Tables 1–7. [file 41565_2025_1939_MOESM1_ESM.pdf]

nature nanotechnology

Supplementary information https://doi.org/10.1038/s41565-025-01939-8

# Evolution of multivalent supramolecular assemblies of aptamers with target-defined spatial organization

In the format provided by the authors and unedited

# Table of Contents


EXTENDED METHODS....3

Computational Modelling of multivalent selection....3
Adaptation of the Liese and Netz model for non-identical ligands....3
Avidity grid preparation....5
*In silico* multivalent selection....5
Synthesis of tyramine-O-Pivalate....6
Synthesis, purification, and characterization of trinucleotide building blocks....6
DNA purification....7
Spike-coated magnetic beads preparation....7
Mass-Photometry measurements....8
High-throughput sequencing and data analysis....8
Production of selected functionalized nucleic acids....10
Scheme 1. Stepwise Synthesis of FNAP....10
AFM imaging....10
Coarse-Grained Simulations....10
DNA secondary structure prediction....11

SUPPLEMENTARY FIGURES....12

Figure S1. Flowchart illustrating the process for multivalent selection modelling....12
Figure S2. Comparison of DNA dendrimer and cssDNA multimerization scaffolds....13
Figure S3: cssDNA scaffold preparation and assembly of MEDUSA with 8-mer FNAP library....14
Figure S4. Fourier Shell Correlation (FSC) analysis assessing the resolution of trimeric assembly's reconstruction....15
Figure S5. MALDI-TOF mass-spectra of HPLC-purified trinucleotide building blocks....20
Figure S6. Characterization of trinucleotide building block libraries....21
Figure S7. FNAP library length considerations and MEDUSA assembly prepared with a longer (12-mer) FNAP library....22
Figure S8. Dynabeads™ MyOne™ Streptavidin C1 loading with SARS-CoV2 spike protein....24
Figure S9. Sequence composition and diversity of final library for monovalent and medusa selection strategies....25
Figure S10. Sequence alignment of m1 and reported SARS CoV-2 spike binding aptamers....25
Figure S11. Frequencies of side-chain modifications in the FNAP library at different round of selection for monovalent (left) and medusa (right) selection strategies....26

Figure S12. Levenshtein distances between the top 150 selected sequences. .......................................27
Figure S13. Primer minimization study for m1 and m2 sequences. .....................................................28
Figure S14. Representative MALDI-TOF mass-spectra of PAGE-purified m1 binding units. ............29
Figure S15. Additional characterization of selected MEDUSAs. .......................................................30
Figure S16: Structure-activity study for m2 and m11 sequences. .......................................................31
Figure S17. Prediction of the secondary structure of the selected m1 FNAP using NUPACK. ...........32
Figure S18. Prediction of the secondary structure of the selected m2 FNAP using NUPACK. ...........33
Figure S20. Selectivity of selected MEDUSAs by SPR. ......................................................................35
Figure S21. Competition ELISA between trivalent assemblies using different ELISA plates. ............36
Figure S22. Competition ELISA between MEDUSAs and a set of reported aptamers. .......................37
Figure S23. OxDNA-generated models of assembly core prepared with 2T, iSp18 and Lin scaffold strands. ..............................................................................................................................................38
Figure S24. PAGE analysis of MEDUSA variants featuring different binding units and scaffold strands. ..............................................................................................................................................39
Figure S25. sMEDUSA FRET construct production and characterization. .........................................40
Figure S26. FRET induction by cooperative multivalent binding in *cis* using guest sMEDUSA and host MEDUSAs of different scaffold configurations. ...................................................................................41
Figure S27. m2 sMEDUSA binding to the spike protein at 37 ºC. .....................................................42

SUPPLEMENTARY TABLES ..............................................................................................................43

Table S1. Modified trinucleotide building blocks. ...............................................................................43
Table S2. Primer sequences ...................................................................................................................44
Table S4. Scaffold strand sequences .....................................................................................................44
Table S5. Binding unit sequences ..........................................................................................................45
Table S6. Aptamers ...............................................................................................................................46
Table S7. SPR kinetic parameters .........................................................................................................47

REFERENCES .......................................................................................................................................49

# Extended methods

### Computational Modelling of multivalent selection

We developed a two-scale computational framework to model the binding dynamics of multivalent particles and receptors (Figure S1).

Scale 1 focuses on a single pair of a multivalent particle and receptor to estimate the overall binding affinity for a specific combination of parameters characterizing the particle. This scale is based on the model proposed by Liese and Nentz[1], which we extended to account for non-identical linker lengths and binding affinities of the ligands presented by the multivalent particle (detailed in "Adaptation of the Liese and Netz model for non-identical ligands"). Using a slightly modified version of the script, available on Github through token, we performed Monte Carlo (MC) integration to sample the various positions and orientations of the multivalent particle relative to the receptor. This enabled us to compute ensemble binding constants (avidities) across a multidimensional parameter space defined by three linker lengths, three binding affinities, and various core sizes, while holding all other parameters constant (see "Avidity grid preparation"). The result was a multidimensional grid of avidities.

Scale 2 expands the analysis to a pool of multivalent particles binding to a pool of receptors. The particles were generated by randomly assembling ligands with linker lengths and binding affinities sampled from prescribed distributions (see "*in silico* multivalent selection"). Using the avidity grid from Scale 1 as a lookup table, and assuming that crosslinking of receptors mediated by multivalent particle to be negligible, we approximated each multivalent particle as a monovalent binder with an equivalent binding affinity derived from its computed avidity. Simulations were conducted using a Gillespie algorithm[2] implemented in MATLAB.

By iteratively repeating simulations at Scale 2, we mimicked a selection procedure. For each cycle, the distribution of particles bound in the previous step was used as input for the next iteration. The code is available on Github through token. Analysis of the selected population from each simulation was performed in MATLAB (https://www.mathworks.com), and the results were plotted using GraphPad (https://www.graphpad.com) for visualization.

### Adaptation of the Liese and Netz model for non-identical ligands.

To address ligand-specific heterogeneity in multivalent binding systems, we extended the original model by Liese and Netz[1] for the dissociation constant ($K_n$) of a n-valent particle binding to an n-valent receptor. The original expression indicates that the specific value of $K_n$ depends on all possible binding modes that a n-valent particle can bind to an n-valent receptor, assumes uniform ligand properties and it is given as:

$$K_n = \left[ \sum_{i=1}^{n} \int d\boldsymbol{r}_0 \int d\boldsymbol{\omega}_0 \frac{1}{8\pi^2} \sum_{\{i,n\}} \left( \prod_{j \in \{i\}} \frac{\Omega_{bp}}{\Omega_{LU}} V_{bp} e^{-\beta \Delta G} P_{str}^{\mathrm{bound}}(r_j) m^i \cdot \prod_{j \notin \{i\}} \int dr_j P_{str}^{\mathrm{im}}(r_j) \right) \right]^{-1} \quad \text{Eq. S1}$$

In particular, term *i* represents the number of ligand units bound to receptor pockets, and the summation over *{i,n}* considers all possible dispositions of these bound units. Each mode is influenced by:

1) The angular steric restriction factor represented by the fraction of $\Omega_{bp}$ over $\Omega_{LU}$, which compares angular space available to the ligand while in the binding pocket ($\Omega_{bp}$) to the angular space available when in bulk ($\Omega_{LU}$).

2) The binding pocket volume $V_{bp}$ and the binding free energy $\Delta G$, which drive the strength of individual binding events.

3) The stretching probabilities $P_{str}^{\text{im}}$ and $P_{str}^{\text{bound}}$, which describe respectively the stretching probability of bound and unbound polymeric linkers depending on the distance $r_j$ of the $j^{th}$ linker from its attaching point in the core of the particle.

The integration over the vectors $\boldsymbol{r_0}$ and $\boldsymbol{\omega_0}$ accounts for spatial and angular fluctuations of the ligand core relative to the receptor.

In our approach, we simply introduce subscripts to distinguish properties of individual ligand units. the revised equation is expressed as:

$$K_n = \left[ \sum_{i=1}^{n} \int d\boldsymbol{r_0} \int d\boldsymbol{\omega_0} \frac{1}{8\pi^2} \sum_{\{i,n\}} \left( \prod_{j\in\{i\}} \left( \frac{\Omega_{bp_j}}{\Omega_{LU_j}} V_{bp_j} e^{-\beta\Delta G_j} P_{j_{str}}^{\text{bound}}(r_j) \right) \prod_{j\notin\{i\}} \int dr_j P_{j_{str}}^{\text{im}}(r_j) \right) \right]^{-1} \quad \text{Eq. S2}$$

Keeping all other definitions identical to the ones in Liese and Nentz[1]. Similarly, to the homogenous case, if we consider the angular space for a free rotating body is $8\pi^2$, we can formulate the monovalent dissociation constant ($K_{1j}$) as:

$$K_{1j} = \frac{8\pi^2}{\Omega_{bp_j}} \frac{e^{\beta\Delta G_j}}{V_{bp_j}} \quad \text{Eq. S3}$$

And by normalizing by the greatest monovalent dissociation constant, we can rewrite Eq. S2 as:

$$K_n = \left[ \sum_{i=1}^{n} \frac{\int d\boldsymbol{r_0} \int d\boldsymbol{\omega_0} \frac{1}{8\pi^2} \sum_{\{i,n\}} \left( \prod_{j\in\{i\}} \left( \frac{P_{j_{str}}^{\text{bound}}(r_j)}{\omega_{LU_j}\alpha_j} \right) \prod_{j\notin\{i\}} \int dr_j P_{j_{str}}^{\text{im}}(r_j) \right)}{K_{1M}^{i}} \right]^{-1} \quad \text{Eq. S4}$$

Where $\alpha_j$ is the ratio between the $K_{1j}$ and the maximum of all the monovalent dissociation constants of the ligands of the particle, $K_{1M}$, and $\omega_{LUj}$ is the angular space in bulk of the $j^{th}$ ligand unit attached to the core when compared to the one of a free unit.

**Avidity grid preparation.**

Following the model just described, we prepared several grids representing the avidities of trivalent particles binding to trivalent receptors. To focus our exploratory study on the effects of core size and ligand properties, we decided to reduce the dimensionality of the whole model by fixing the multiplicity of each ligand $m_j$ as 1 and the $\omega_{LUj}$ to the representative value of 0.03, a result reported by Liese and Nentz[1] while fitting experimental data of homo-multivalent particles. The receptor core size was fixed at 12 nm, while the particle core sizes tested were: 2, 7, 10, 12, 14, 17, and 22 nm. Linker lengths were expressed in PEG units ranging from 6 to 49 in increments of 6 (estimated to correspond to lengths of 1–4 nm[1]).

the weighting factors $\alpha_1$, $\alpha_2$, and $\alpha_3$ were used during integration to normalize contributions of different binding configurations. These factors satisfy the hierarchy $\alpha_1 \geq \alpha_2 \geq \alpha_3$ , with $\alpha_1$ fixed at 1, while $\alpha_2$ and $\alpha_3$ were systematically generated to explore the parameter space:

- $\alpha_2$, sampled from an array of exponentially spaced values :

$$\alpha_2 = e^{linspace(-8,0,16)}$$

- $\alpha_3$, sampled as :

$$\alpha_3 = \alpha_2 \tan(\varphi\pi)\,, with\ \varphi = e^{linspace(-9.35,\log(0.25),22)}$$

with the condition that $\alpha_3 \leq \alpha_2$.

The specific parameter limits were empirically chosen to ensure that the particles simulated at Scale 2 fell within the bounds of the grid.

***In silico* multivalent selection**

The modeling of multivalent selection was conducted by randomly assembling particles from a distribution of ligands characterized by a lognormal distribution of dissociation constants $k_D$s centered at 10-4.5 M, with a standard deviation of 0.85 in log units. Linker lengths were modeled using a truncated Gaussian distribution ranging between 1 and 4.5 nm, starting from a Gaussian distribution of mean 2.53 nm and standard deviation of 0.882 nm. A total of 106 particles and 3.33*105 receptors were simulated to mimic the experimental conditions used during multivalent selection. In the absence of prior information on rate constants for our system, we set the dissociation rate constant koff for each particle equal in magnitude to its  KD through time rescaling. The association rate kon was then fixed to be the same for each particle and chosen so that, after time rescaling and the conversion from receptor concentration to absolute receptor number, it was equal in magnitude to the ratio of the desired particle concentration (in moles) to the number of particles used. This approach, within a first-order kinetic binding model, preserved equilibrium effects across different particle concentrations but prevented a direct correspondence with physical time units.

The system was allowed to evolve for 100 sweeps. We then introduced the stringency, by conserving only the bound particles and letting the simulation run for other 10 sweep. The particles that remained bound during this phase were designated for amplification, ensuring that the final distribution from this round matched the initial distribution of the subsequent round, enabling the iterative assembly of new molecules.

To investigate the influence of structural parameters, simulations were conducted for different core sizes, identical to those described in the "Avidity Grid Preparation" section.

**Synthesis of tyramine-O-Pivalate**

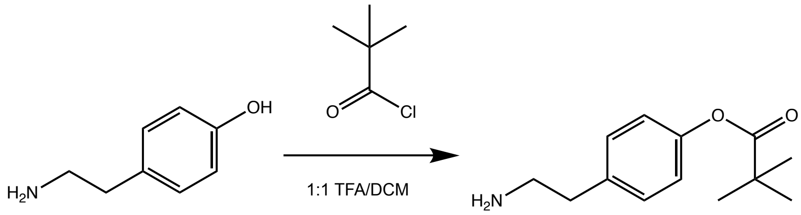


Tyramine (3 g, 21 mmol, 1 equiv) was dissolved in 40 mL of DCM:TFA = 1:1 at 25°C. Pivaloyl chloride (2.69 mL, 21 mmol, 1 equiv) was added dropwise while stirring. The resulting brown mixture was stirred for 12 hours. The reaction mixture was concentrated under reduced pressure and purified by flash column chromatography (Silica, DCM:MeOH = 9:1 to 1:1) to yield the product. **$^1$H NMR** (400 MHz, CDCl3) d 7.21 (d, J=8.53 Hz, 2 H), 6.95 (d, J=8.53 Hz, 2 H), 3.18 (t, J=7.28 Hz, 2 H), 2.90 - 2.99 (m, 2 H), 1.34 (s, 9 H). **ESI-MS**: predicted m/z = 222.15, observed m/z = 222.12 $[m+H]^+$, 443.14 $[2m+H]^+$.

**Synthesis, purification, and characterization of trinucleotide building blocks**

The synthesis of trinucleotide building blocks has been performed using the automated DNA/RNA synthesizer (Oligomaker ApS, Denmark) on a 200 nmol scale (CPG resin, 100 pcs, TAG Copenhagen AS, OM-11-96-01). The essential materials for custom DNA synthesis were dC(ac) amidite (TAG Copenhagen AS, C113080), dG(dmf) amidite (TAG Copenhagen AS, G115080), dA(bz) amidite (TAG Copenhagen AS, A111080), T amidite (TAG Copenhagen AS, T111080), NHS-Carboxy-dT amidite (Glen Research, 10-1535-XX). For incorporation of 5' phosphate into trinucleotide sequences, Chemical Phosphorylation Reagent II (Glen Research, 10-1901-XX) has been used. Side-chain modifications were introduced into trinucleotides by coupling C5-carbxy-NHS-modified dT base at the 5' end of trinucleotide followed by on-resin amidation with the library of primary amines. Two consecutive 6 min couplings were used for all non-standard phosphoramidites. For amidation, 300 equivalents of primary amine in 200 uL of DMSO:Acetonitrile = 10:90 were added onto the column after the coupling and detritylation of Chemical Phosphorylation Reagent II. All trinucleotides were synthesized DMT-off, cleaved and deprotected with 200 uL of 30% aqueous Ammonium Hydroxide overnight at RT. After the cleavage, the column was washed with additional 200 uL of 30% aqueous

Ammonium Hydroxide solution. Ammonium Hydroxide was evaporated using vacuum concentrator (SpeedVac™ DNA130) at 65 ºC for 1h. The remaining solution was centrifuged at 15,000 g for 10 min to remove the silica from CPG solid support that is typically carried over into the solution during ammonium hydroxide cleavage and deprotection. Trinucleotides were then purified by RP-HPLC. The gradient for functionalized trinucleotides: 0-20% of acetonitrile in 100 mM TEAA, pH 7.0 over 22 min, followed by 20-40% of acetonitrile in 100 mM TEAA, pH 7.0 over 8 min. For purification of non-functionalized trinucleotides, the following gradient has been used: 5-20% of acetonitrile in 100 mM TEAA, pH 7.0 over 10 min, followed by 20-40% of acetonitrile in 100 mM TEAA, pH 7.0 over 2 min. The fractions of interest were collected and lyophilized. Purified trinucleotide building blocks were analyzed by MALDI-TOF mass spectrometry using 50 mg/mL 3-HPA, 10 mg/mL DAHC matrix. MS-spectra were acquired with a MALDI-TOF/TOF AutoFlex Speed (Bruker) mass spectrometer. The device was operated in the negative ion mode using reflective TOF (Figure S5).

**DNA purification**

To purify the circular ssDNA, FNAPs and template library, a 10% PAGE with 8M urea was performed. 13 mL of the solution (10% acrylamide:bisacrylamide = 19:1, 8M urea, 1x TBE (89 mM Tris-HCl, 89 mM boric acid, 2 mM EDTA, pH 8.0), 12 µL TEMED, and 80 µL of 10% APS were used to cast one 1.5 mm gel (HasLab). Five microliters of the sample were loaded per well (1 – 15 µg of crude DNA), and the gel was run at 180 V for 1.5 hours (power supply - Labgene scientific, cell - Biometra). The bands were visualized by briefly exposing the gel to 260 nm UV light against the TLC plate. The band of interest was then excised, the gel slice was crushed, and the oligo was extracted using 2 mL of 200 mM NaCl, 40 mM TE buffer, pH 7.5. The first extraction step was carried out at 37°C overnight on a shaker. After the initial extraction, the gel slurry was filtered out using Freeze 'n Squeeze (BioRad, #7326165) spin filters, and the gel was subjected to a second extraction with 2 mL of RF water for 2 hours at 37°C on a shaker. The extracts were collected into a 15 mL tube. Two volumes of dry n-butanol were added to the extract (creating an organic layer on the top), vortexed, and removed three times. After the volume of the extract was reduced to 300 µL, purified oligos were precipitated using 3 M sodium acetate and EtOH. After centrifugation, the pellets were washed with cold 70% EtOH and air-dried. The pellets were then reconstituted in 75-100 µL of restriction enzyme-free water, and the concentration was measured using NanoDrop based on the absorbance at 260 nm.

**Spike-coated magnetic beads preparation**

Dynabeads™ MyOne™ Streptavidin C1 magnetic beads (ThermoFisher) were used for affinity selections. Prior to use, the beads were washed three times by adding one volume of 1x PBS followed by magnetic separation for 1 minute in a magnetic stand. Then, the beads were loaded with Twin-StrepTag-tagged stabilized Spike trimer (2P mutation) at 20 µg of protein per 1 mg of resin at a 100 µg/mL protein concentration (two volumes to the original 1% bead stock solution). The suspension was

incubated on a rotary mixer at 4°C for 1.5 hours. The supernatant was then discarded, and the beads were washed ten times with two volumes of SW Buffer (1xDPBS, 1 mM MgCl2, 0.1 mg/mL BSA, 0.005% Tween 20). This procedure yielded an immobilization level of 10 µg of Spike-trimer per 1 mg of beads (~50% of maximal load capacity, Figure S8). Finally, the beads were washed 5 times for 2 minutes with one volume of SW Buffer.

**Affinity selections**

A library of trivalent assemblies of FNAP was prepared by annealing the purified FNAP library with the cssDNA scaffold strand in 3.3:1 molar ratio (330 nM FNAP and 100 nM cssDNA) in 1xDPBS, 1mM $MgCl_2$ using the following thermocycler program: 95°C for 2 minutes, 80°C to 20°C at -2°C/cycle (30 cycles). The monovalent FNAP library was prepared at a final concentration of 330 nM in the same buffer and subjected to the same thermocycle program. After folding, a small sample of 0.33 pmol of trimeric and 1 pmol of monovalent FNAP libraries were taken as a backup. Next, both monovalent and multivalent libraries were subjected to negative selection against blank Dynabeads™ MyOne™ Streptavidin C1 magnetic beads by incubating the library with an equal amount of beads as used for the target protein immobilization for 1 hour at room temperature on the rotary mixer. Then, the libraries were incubated for 1 hour with target-coated beads. After incubation, the beads were washed three times with 1xDPBS, 1 mM MgCl2, 0.1 mg/mL BSA, 0.005% Tween20 for 5 minutes per washing step on the rotary mixer. After the last wash, binders were eluted by heating the beads suspended in 50 µL of RF water for 10 minutes at 95 °C. The negative selection blank beads were processed the same way to estimate the nonspecific binding of the library during the final three selection rounds. The progress of affinity selections was monitored by qPCR, comparing the amount of FNAP in the elution fraction to that in the flow-through fraction.

**Mass-Photometry measurements**

To evaluate the binding capacity of the libraries of trivalent assemblies at different selection rounds, Refeyn Two MP mass photometer (Refeyn Ltd.) has been used. Firstly, a calibration curve was obtained using BSA solution. For this, 5 µL of 10 µg/mL of BSA in 1xPBS were introduced into 15 µL of 1xPBS previously deposited into one of the wells of a silicon gasket. For library measurements, 1.5 µL of 200 nM library of assemblies in 1xPBS, 1 mM MgCl2 were incubated with 3 uL of 62 ug/mL Spike protein in 1xPBS for 1 h. Then, 4.5 µL of the mixture were introduced into 15 µL of 1xPBS that were previously deposited into one of the wells of the silicon gasket. All single-molecule light scattering events were recorded for 180 sec.

**High-throughput sequencing and data analysis**

For sequencing, a sample of 30 fmol of FNAP library was taken from the elution fractions of selection rounds 3, 6, and 7 for both monovalent and multivalent selection strategies. Two consecutive PCR amplifications by Q5 DNA polymerase were performed to install Illumina adaptors (pcr1) and index

sequences (pcr2). The first PCR was performed in 2 reactions of 25 μL each using Q5 DNA polymerase. After 10 PCR cycles, the reactions were pooled, and the amplicons were purified using the Monarch PCR & DNA Cleanup Kit. Subsequently, the amplicons were purified using 8% native TBE PAGE. The second indexing PCR was set up in a 50 μL reaction using Q5 DNA polymerase according to the following indexing scheme:

monovalent selections elution fraction from round 3 – S502+N704, monovalent selections elution fraction from round 6 – S503+N705, monovalent selections elution fraction from round 7 – S504+N706, medusa selections elution fraction from round 3 – S502+N701, medusa selections elution fraction from round 6 – S503+N702, medusa selections elution fraction from round 7 – S504+N703. The indexed libraries were then purified using 6% native TBE PAGE followed by Monarch PCR & DNA Cleanup Kit. The concentrations were measured using the Qubit (Invitrogen™ Qubit™ Flex), and the indexed libraries were submitted for sequencing using the NovoSeq (multiplexed monovalent selections elution fraction from round 3, monovalent selections elution fraction from round 6, monovalent selections elution fraction from round 7, medusa selections elution fraction from round 3, medusa selections elution fraction from round 6, and medusa selections elution fraction from round 7; 20 million reads per library) and MiSeq (15 million reads for multiplexed monovalent selections elution fraction from round 7 + medusa selections elution fraction from round 7) systems.

Primary filtering of the raw NGS data has been done in awk using the following commands:

For MiSeq data:

awk'/CCTCTACCACCTACATCACTCCT[A,T,G,C][A,T,G,C][A,G,C][A,T,G,C][A,T,G,C]T[A,T,G,C][A,T,G,C][A,G,C][A,T,G,C][A,T,G,C]T[A,T,G,C][A,T,G,C][A,G,C][A,T,G,C][A,T,G,C]T[A,T,G,C][A,T,G,C][A,G,C][A,T,G,C][A,T,G,C]GGTGAAAG/ {print; getline; print; getline; print}' <run name>.fastq > <run name>_filtered.fastq

For NovaSeq data:

awk'/CCTCTACCACCTACATCACTCCT[A,T,G,C][A,T,G,C][A,G,C][A,T,G,C][A,T,G,C]T[A,T,G,C][A,T,G,C][A,G,C][A,T,G,C][A,T,G,C]T[A,T,G,C][A,T,G,C][A,G,C][A,T,G,C][A,T,G,C]T[A,T,G,C][A,T,G,C][A,G,C][A,T,G,C][A,T,G,C]GG/ {print; getline; print; getline; print}' <run name>.fq > <run name>_filtered.fastq

Then, the homemade python script was used to calculate the frequencies of the sequences. The code is available on Github through token. The top 100 sequences from the resulting file, along with their sequence counts, were used for multiple sequence alignment (MSA) using ClustalOmega (https://www.ebi.ac.uk/jdispatcher/msa/clustalo?stype=dna), and the guide tree was plotted using FigTree v1.4.4 software (http://tree.bio.ed.ac.uk/software/Figtree/). The file containing the sequence-count-weighted top 100 sequences from the elution fraction of round 7 of the monovalent selection strategy was used to produce the consensus sequence for the reads detected in this library (Figure 3d, nested graph). For this, the Python logomaker library was used. All Python scripts for downstream data

analysis (UMAP analysis, Figure S9; side-chain frequency calculation, Figure S11; Levenshtein distance matrix generation, Figure S12) are available on GitHub through a token.

**Production of selected functionalized nucleic acids**

All selected hit sequences were synthesized on a 200 nmol or 40 nmol scale in 5 (for 8-mer sequences) or 7 (for 12-mer sequences) consecutive runs on the automated DNA/RNA synthesizer (Oligomaker ApS, Denmark). Each run ended with the addition of NHS-Carboxy-dT amidite (Glen Research, 10-1535-XX), followed by on-resin amidation with the corresponding primary amine. The stepwise synthetic strategy is depicted in the scheme:

**Scheme 1. Stepwise Synthesis of FNAP**

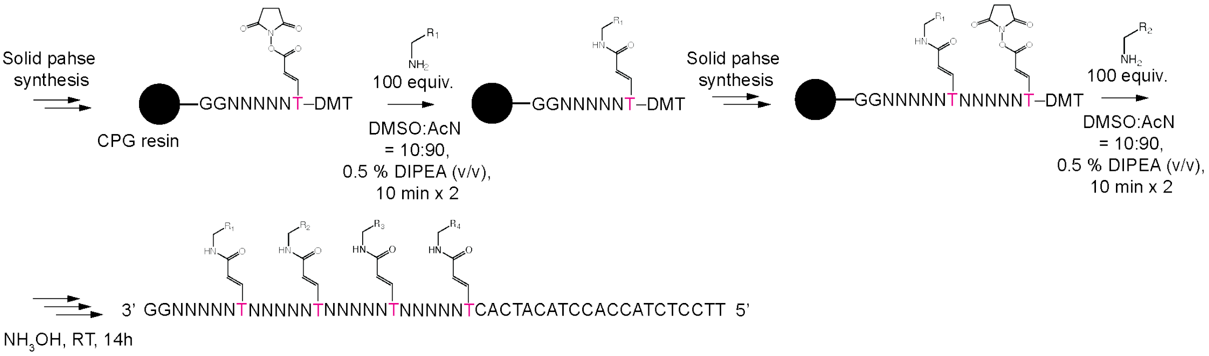


Importantly, this synthetic strategy is widely accessible and can be easily automated to perform iterative amidations of 5' terminal NHS-Carboxy-dT bases on the growing strand, resulting in the desired FNAP sequence. Our typical synthetic yields for an 8-mer FNAP binding unit (50 nt, 4 modifications) at a 200 nmol scale were approximately 1 mg (~70 nmol, ~40%). After overnight cleavage & deprotection with 40% aqueous ammonium hydroxide at room temperature, the crude FNAP was precipitated using 3 M sodium acetate and EtOH and PAGE-purified as described in "DNA purification" section.

**AFM imaging**

Mica (grade V1, Ted Pella) was pre-treated with 200 μL of 100 mM $NiCl_2$. After a brief incubation period, the mica was thoroughly washed with 20 mL of Milli-Q water to remove excess $NiCl_2$. Following this, 5-20 μL of the sample solution was deposited onto the treated mica surface. To prevent bubble formation while approaching the surface, 20 μL of 10 mM $MgCl_2$ was added to the AFM cantilever. The DNA structures adsorbed on the surface were imaged in tapping mode in liquid using a Cypher VRS AFM (Asylum Research Inc.), with a BioLever mini cantilever (BL-AC40TS-C2, Olympus). The image resolution for AFM imaging was maintained at a pixel size of 3 nm or smaller.

**Coarse-Grained Simulations**

All the structures were simulated using molecular dynamics (MD) implementing a modified version of the oxDNA2[3] model. While the core was modelled completely, the functionalized arms were reproduced up to the first three nucleotides (5'-TCC-3') for convenience. To incorporate PEG monomers, steric hindrance from the aromatic base and electrostatic repulsion in the "Dummy" class of

monomers were removed. Initial configurations and topology files were generated using the oxView webserver[4], with manual adjustments made to accommodate the dummy monomers.

All simulations were conducted in the NVT ensemble with an ionic concentration of 0.154 M to replicate experimental conditions. The protocol began with a steepest descent relaxation step, followed by a gradual temperature ramp-up through 1, 5, 10, 15, 20, 25, 30, and 37 °C. During the warming phase, base-pair constraints were applied using oxView's automated tools. In the final step of warming at 37 °C, the base-pair forces were first halved and then fully removed before initiating the production run.

A timestep of $10^{-3}$ was employed, with configurations saved every $5\times10^6$ steps. For each construct, 9 replicas were executed and at least 10,000 configurations from each replica were sampled to ensure statistical significance. Unless stated otherwise, all additional parameters and units adhered to the default settings of the oxDNA platform.

Post-simulation analyses were performed following established protocols described elsewhere[5,6], using as reference for the end-to-end distances the last C on the functionalized arms (Figure S22).

**DNA secondary structure prediction**

DNA secondary structure prediction was performed on the Nupack (https://nupack.org) web server using parameters that correspond to the binding and selection conditions: 25 °C folding temperature, 0.15 M NaCl and 0.001 M $MgCl_2$.

# Supplementary figures

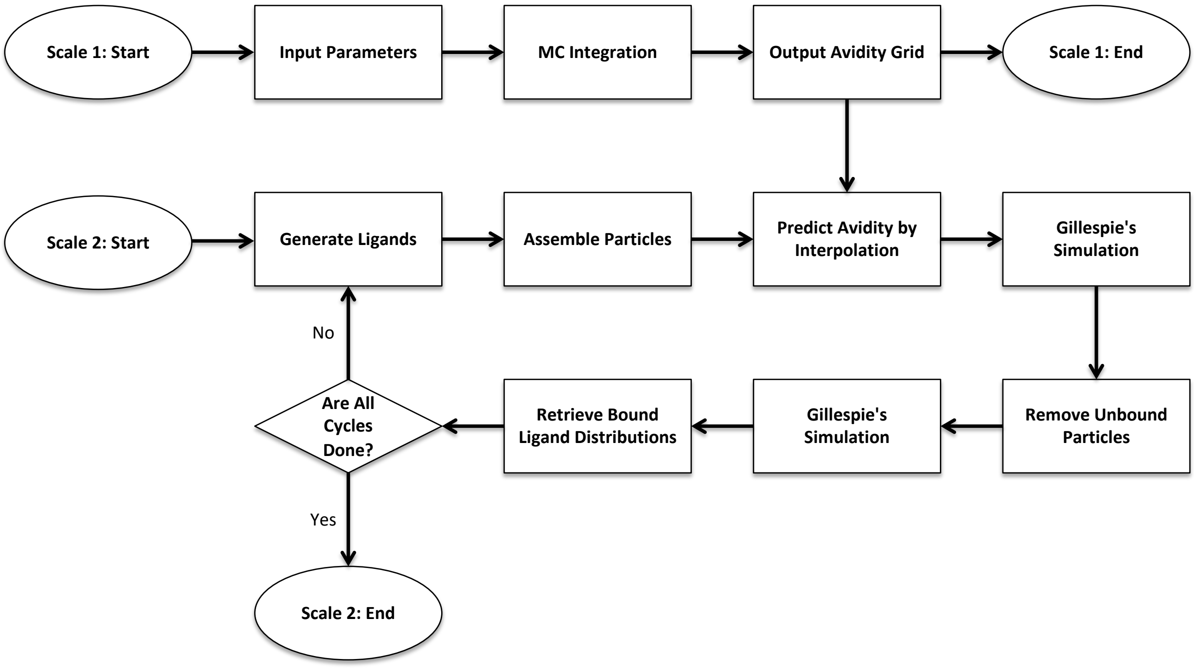


**Figure S1. Flowchart illustrating the process for multivalent selection modelling**

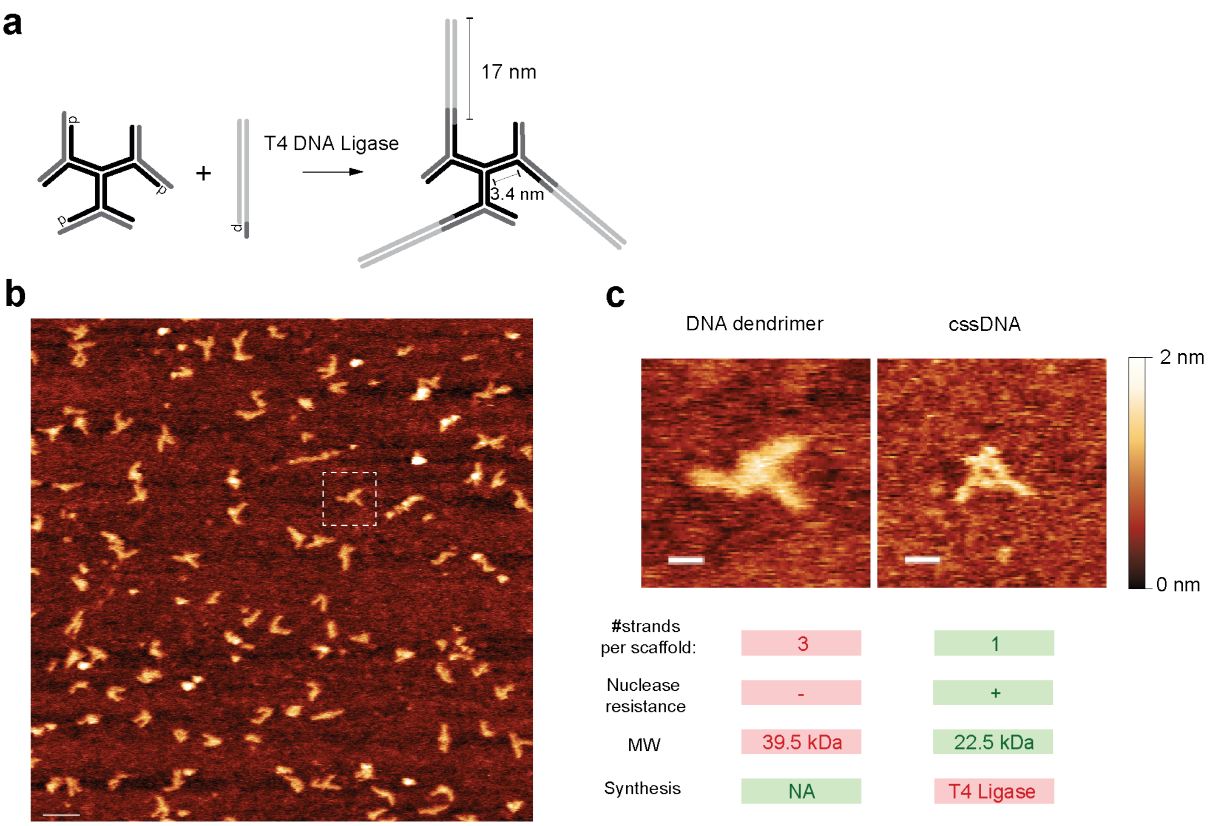


**Figure S2. Comparison of DNA dendrimer and cssDNA multimerization scaffolds.**

**a**, Addition of imaging extensions to the DNA dendrimer core using T4 DNA Ligase. **b**, AFM image of the purified ligation product, scale bar: 50 nm. **c**, Key characteristics of DNA dendrimer and cssDNA multimerization scaffolds. Representative AFM images scale bar: 10 nm.

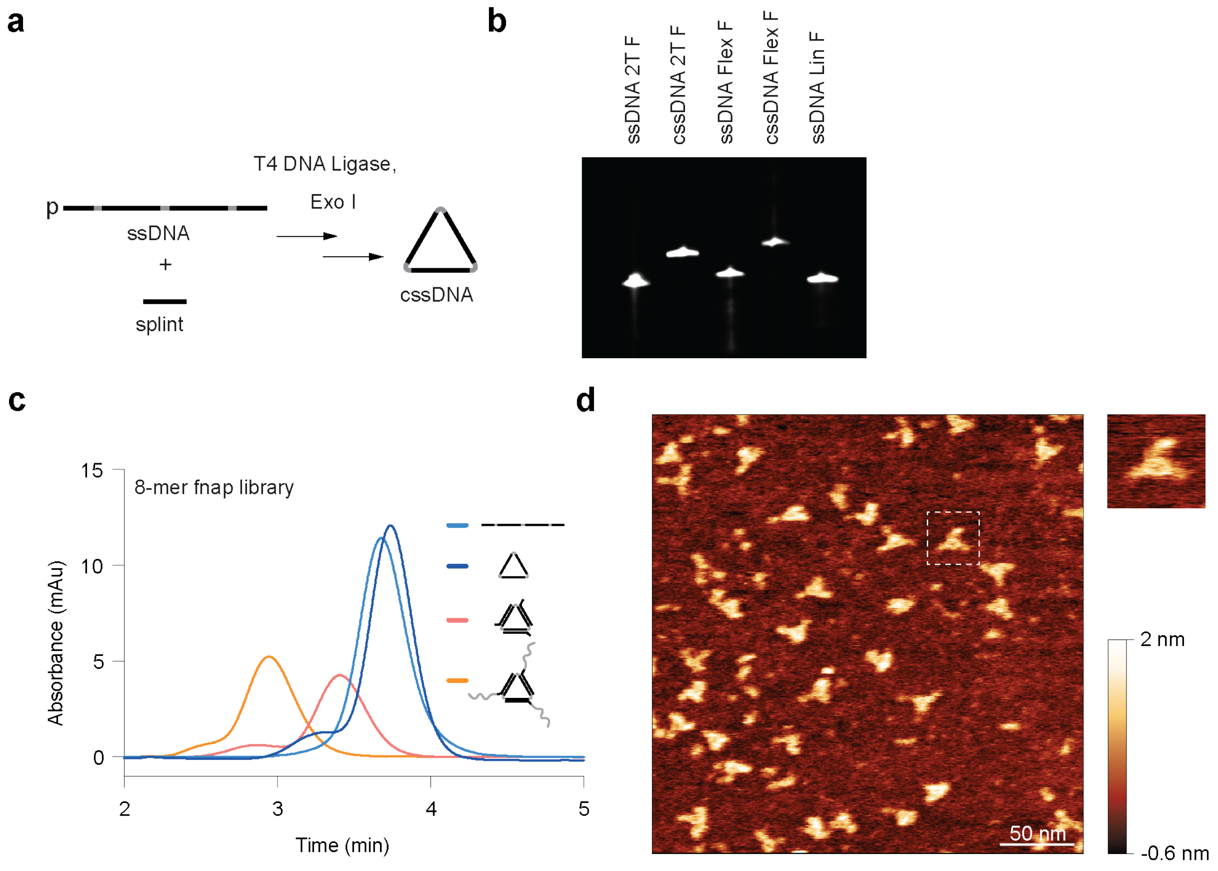


**Figure S3: cssDNA scaffold preparation and assembly of MEDUSA with 8-mer FNAP library.** **a**, Scheme of T4 DNA Ligase-mediated preparation of cssDNA scaffolds. **b**, Analysis of cssDNA scaffolds using denaturing PAGE. Scaffold sequences are provided in Table S4. **c**, Size-exclusion chromatography (SEC) analysis of MEDUSA assembly with 8-mer FNAP library. **d**, AFM images of assembled MEDUSA library of 8-mer FNAP library at selection round 1.

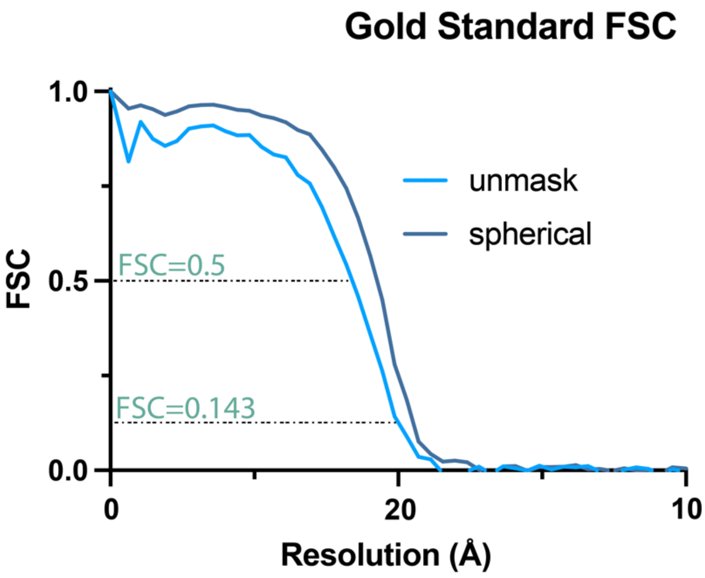


**Figure S4. Fourier Shell Correlation (FSC) analysis assessing the resolution of trimeric assembly's reconstruction.**

The FSC curve indicates a resolution of 20 Å at the 0.143 threshold.

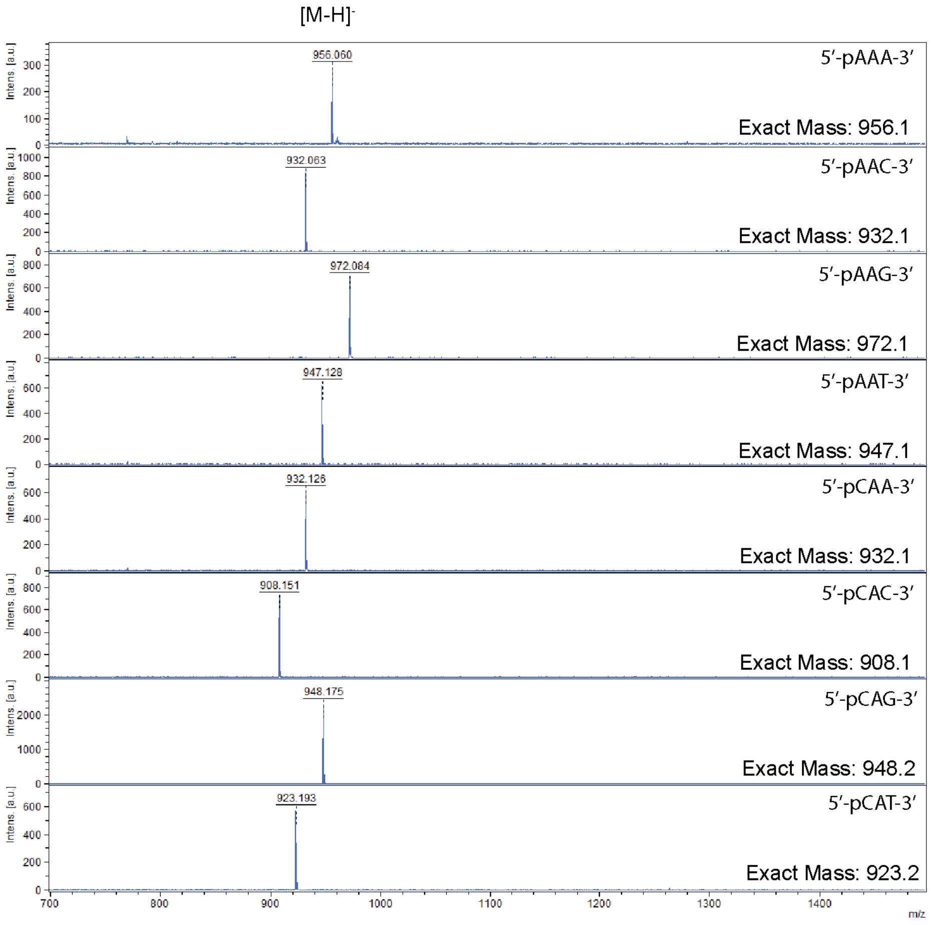


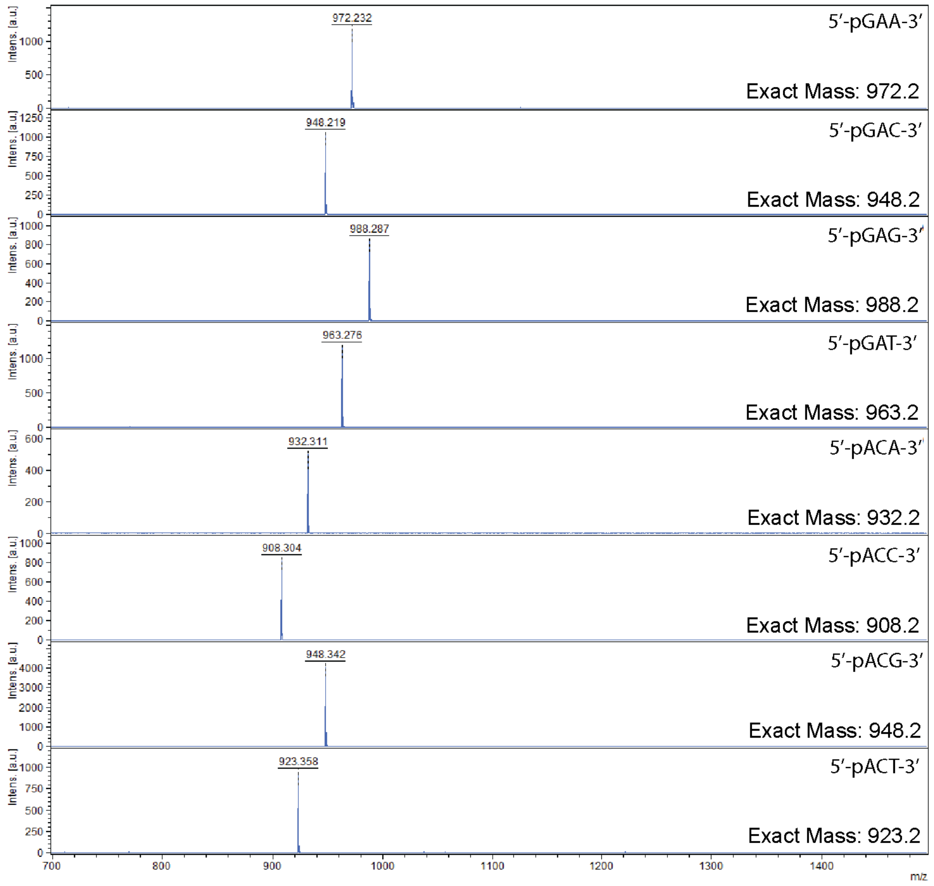


<Figure S5 continues on the next page>

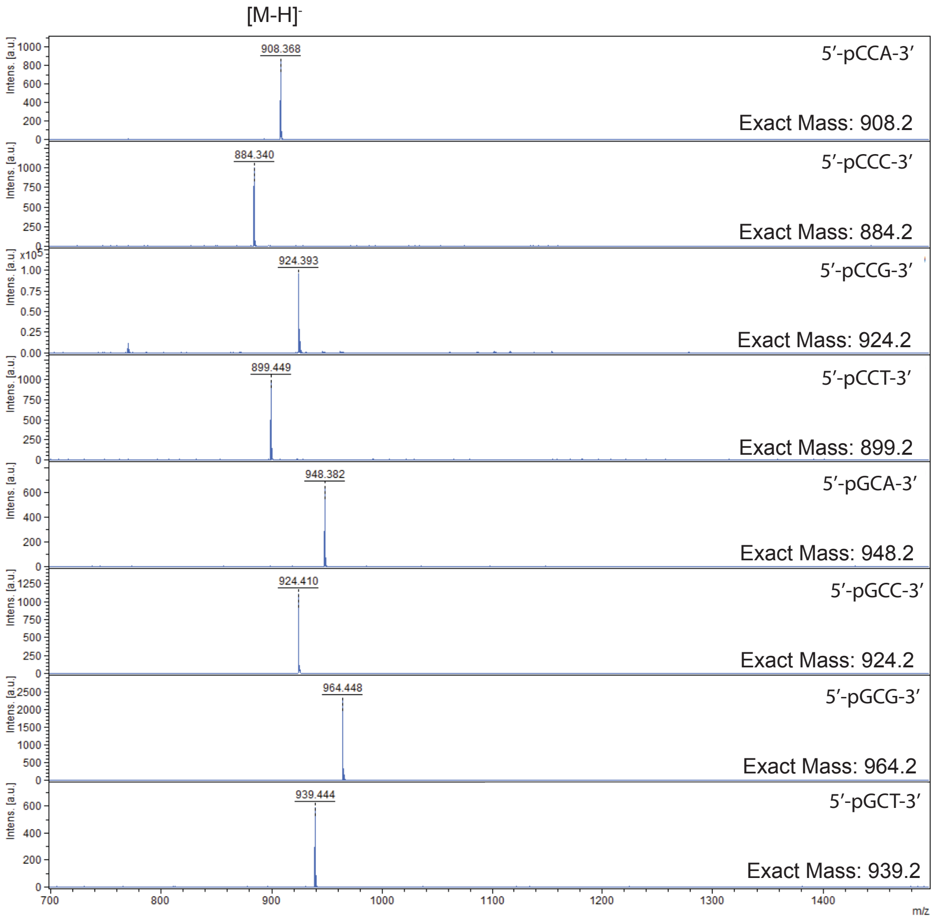


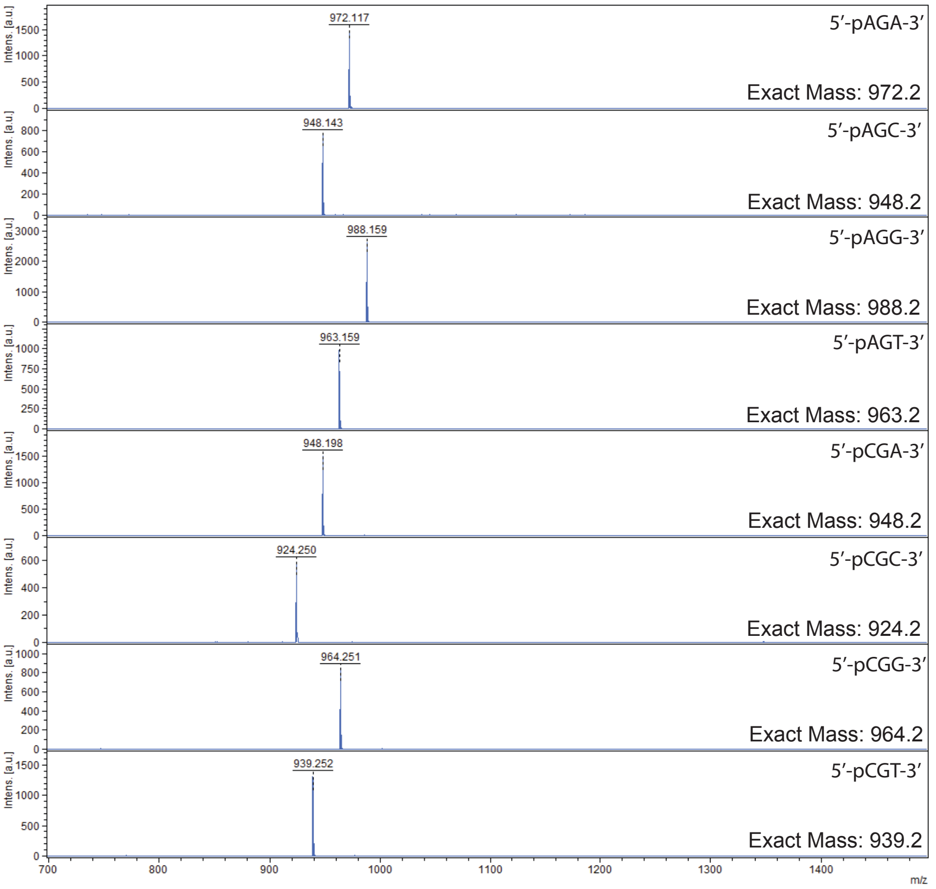


<Figure S5 continues on the next page>

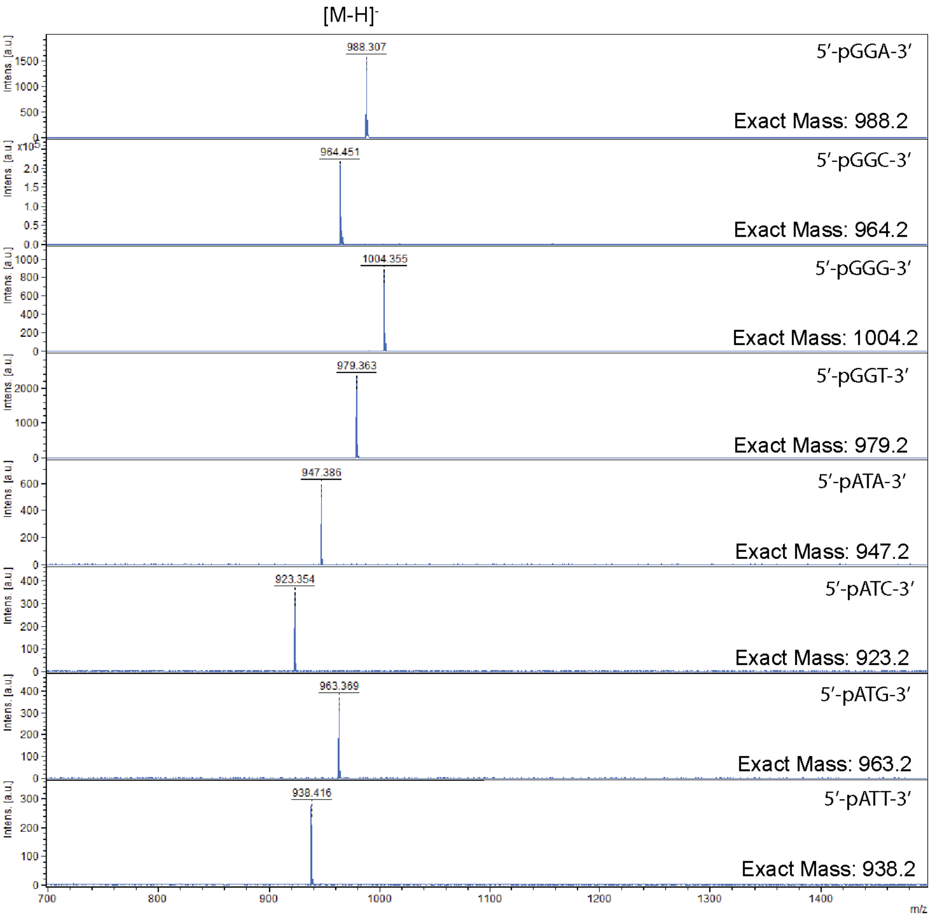


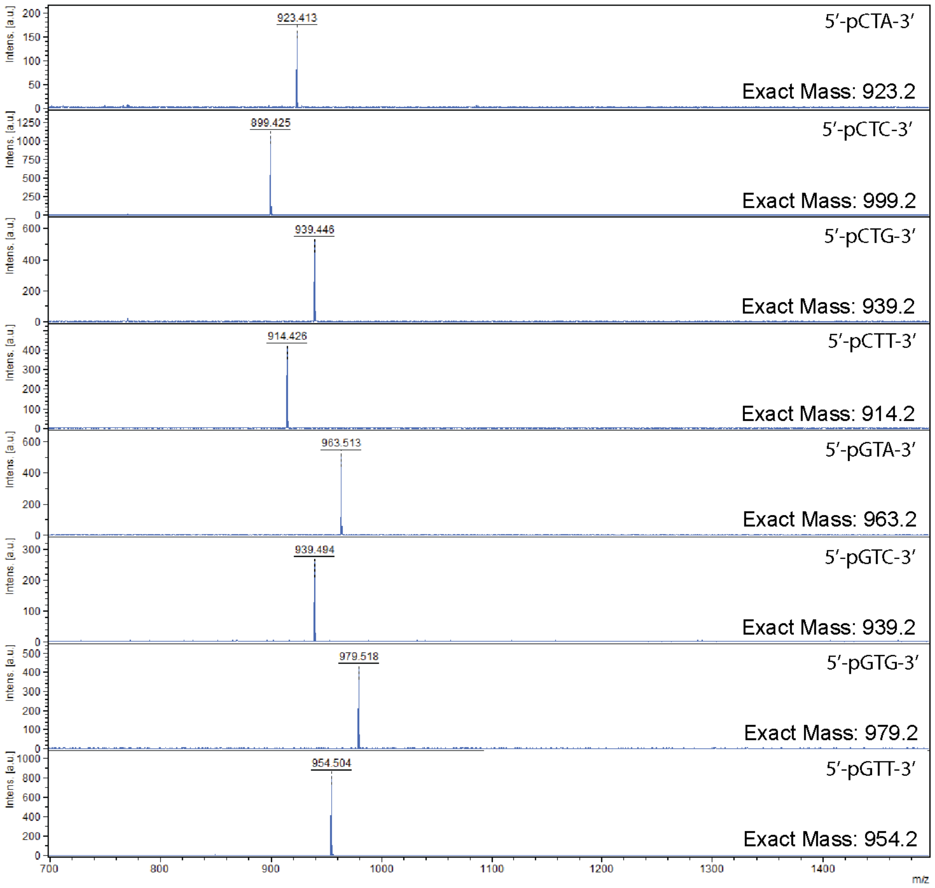


<Figure S5 continues on the next page>

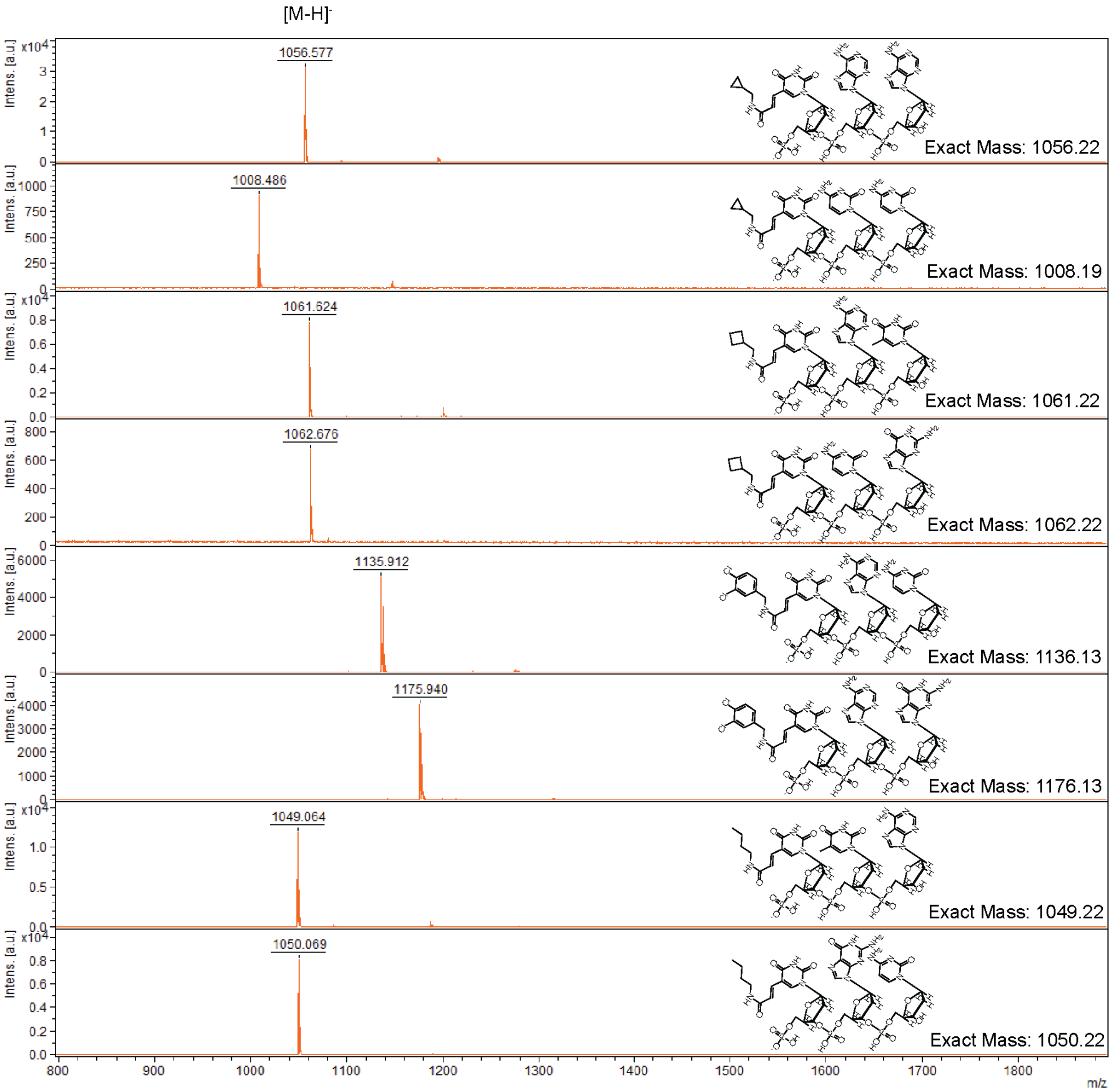


<Figure S5 continues on the next page>

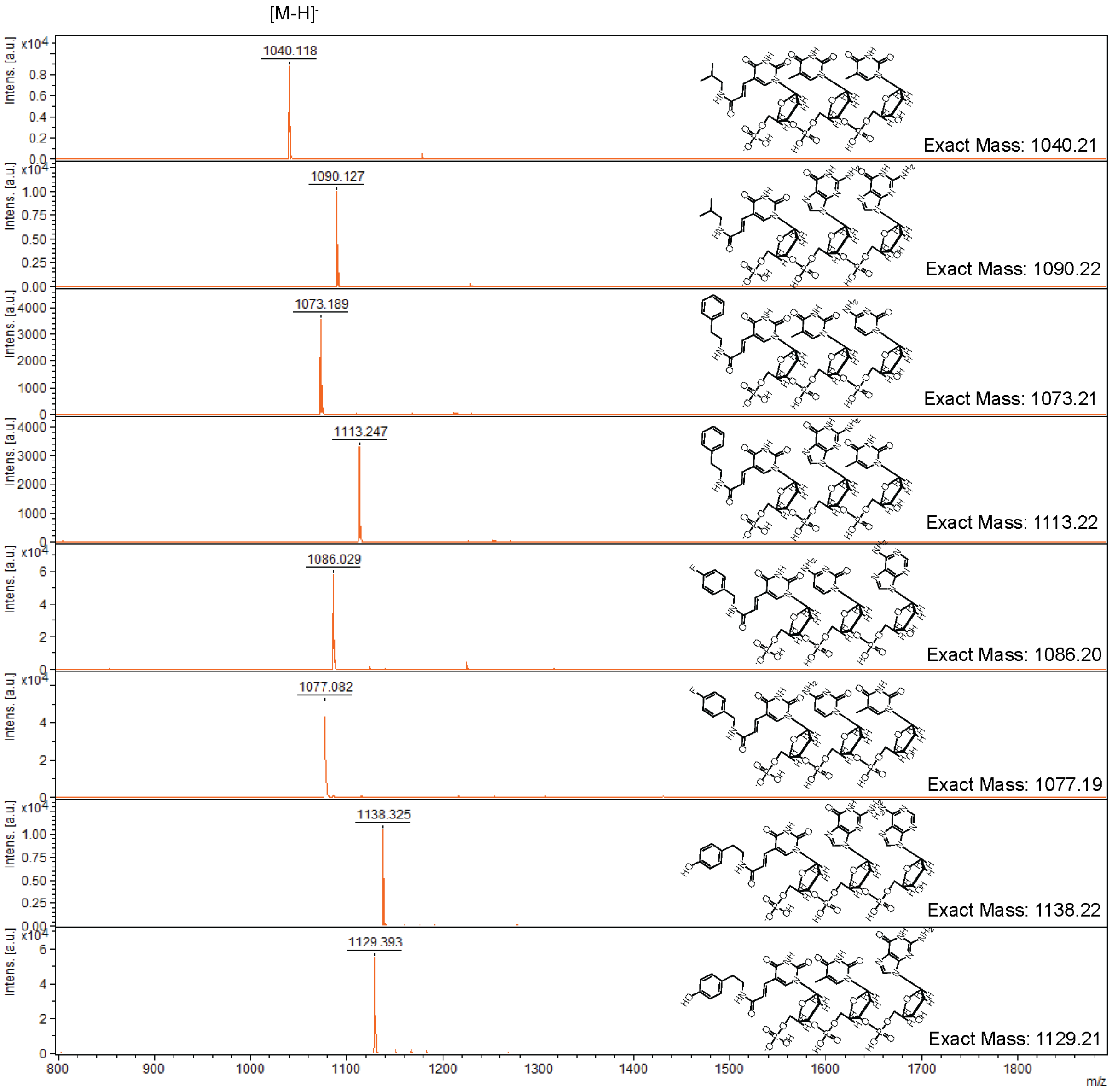


**Figure S5. MALDI-TOF mass-spectra of HPLC-purified trinucleotide building blocks.**

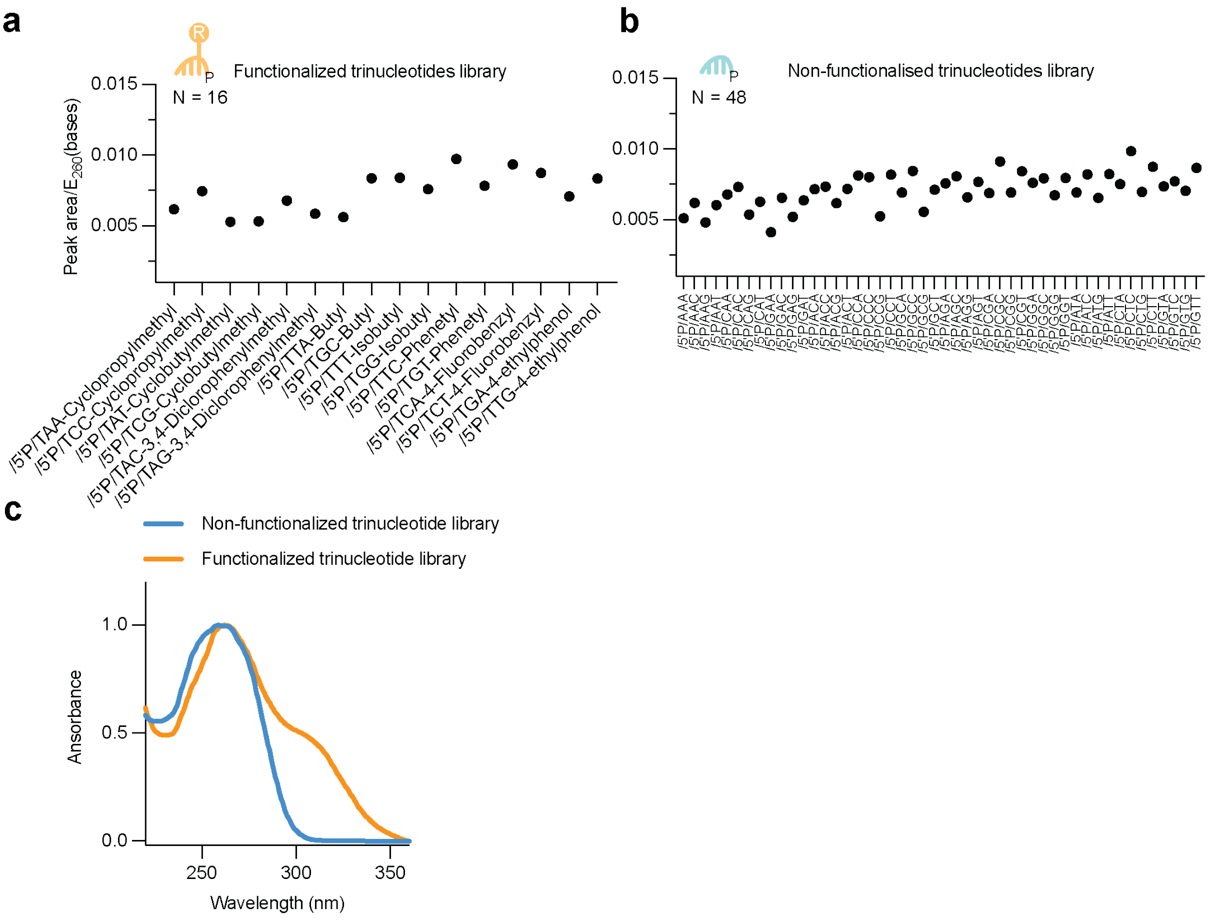


**Figure S6. Characterization of trinucleotide building block libraries.**

Functionalized (**a**) and non-functionalized (**b**) trinucleotide libraries' composition estimated my HPLC peak area of collected fractions for individual trinucleotides. **c**, UV-VIS absorbance spectra of functionalized and non-functionalized trinucleotide libraries.

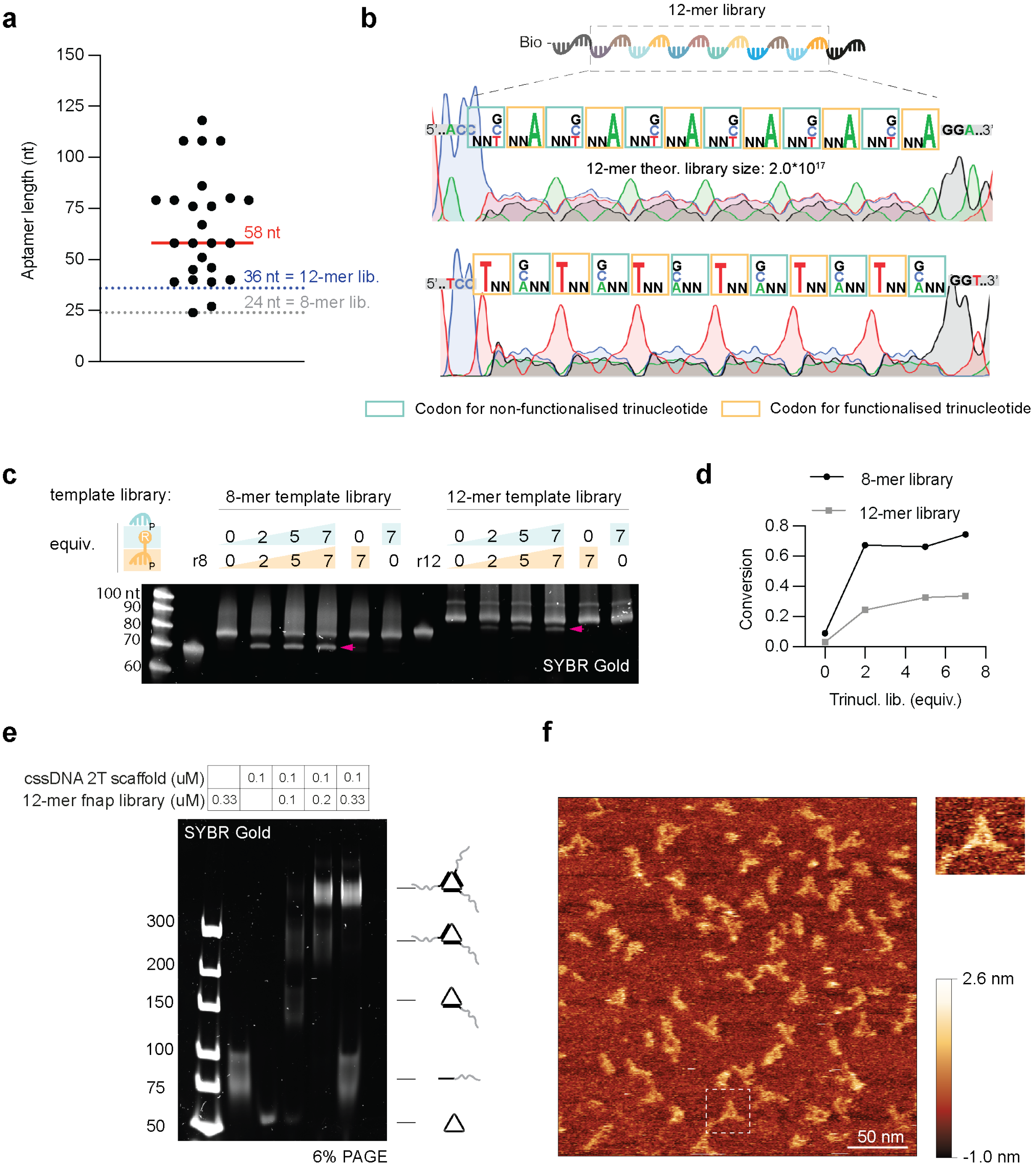


**Figure S7. FNAP library length considerations and MEDUSA assembly prepared with a longer (12-mer) FNAP library.**

**a**, Length distribution of some of the reported SPIKE-protein-binding aptamers[7].The median of the reported sequences is labelled in red. **b**, Structure of the 12-mer template library with corresponding Sanger sequencing electrophoretic traces for direct and reverse sequencing runs. **c**, Denaturing PAGE analysis of the translation reactions of the biotinylated 12-mer template library into the corresponding FNAP library, performed at different trinucleotide library concentrations. R12 (non-biotinylated 12-mer template library) was loaded as a reference. Translation data for the 8-mer library is displayed for comparison. **d**, Comparison of the conversion yields between the 8-mer and 12-mer template libraries, calculated as the ratio of FNAP product band intensity to template library band intensity. **e**, Native

PAGE analysis of the assembly of MEDUSA using the synthesized 12-mer FNAP library. **f**, AFM images of MEDUSA library assembled using the 12-mer FNAP library.

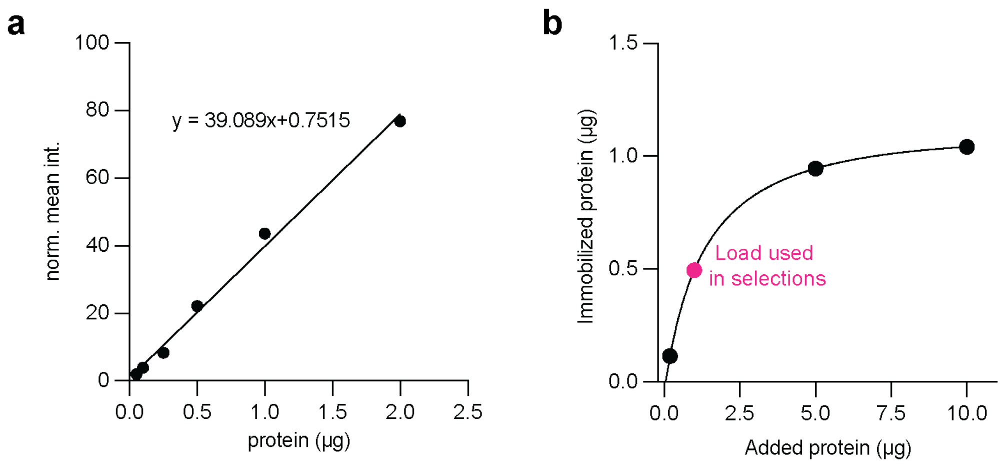


**Figure S8. Dynabeads™ MyOne™ Streptavidin C1 loading with SARS-CoV2 spike protein.**
**a**, Calibration curve for the S-protein, obtained by measuring band intensities from different protein loads on SDS-PAGE. **b**, Loading capacity of streptavidin-coated magnetic beads. Following the capture of S-protein on the beads, the beads were washed, and the quantity of immobilized S-protein was determined via SDS-PAGE. 50% of maximal loading capacity was used for selection experiments.

**a**

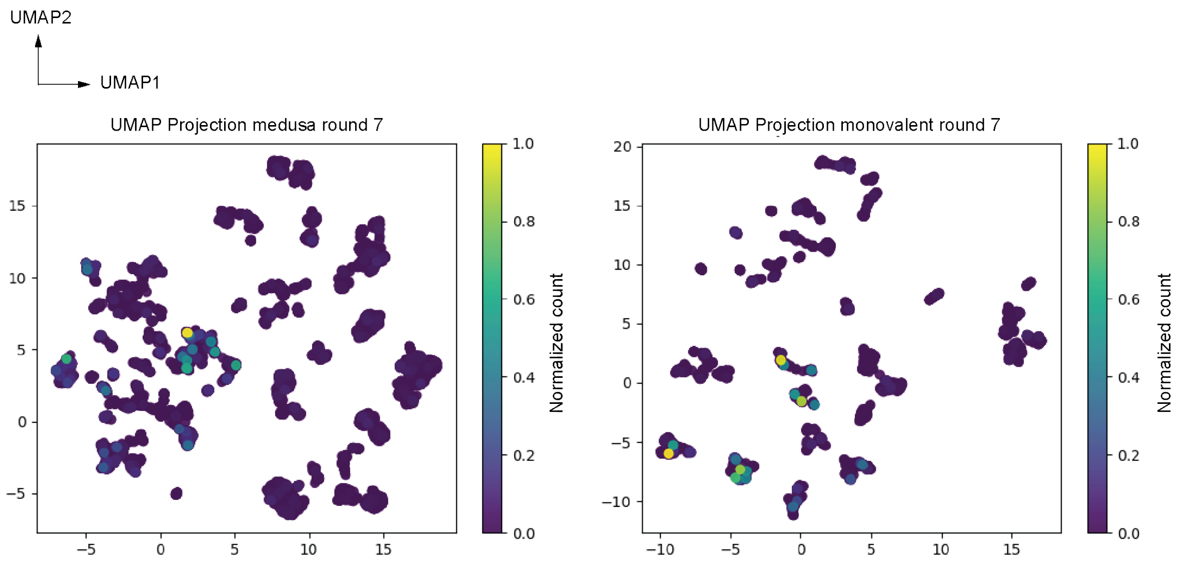


**b**

| Library ID | H | $N_{reads}$ | $N_{unique\ sequences}$ |
|---|---|---|---|
| medusa r7 | 9.41 | 5'834'528 | 1'019'716 |
| monovalent r7 | 5.03 | 5'613'165 | 220'838 |

**Figure S9. Sequence composition and diversity of final library for monovalent and medusa selection strategies.**

**a**, UMAP projections of FNAP libraries at selection round 7 for medusa (left) and monovalent (right) selection strategies. **b**, Sequence diversities of medusa and monovalent FNAP libraries after selection round 7, represented by the Shannon diversity index (H). The library obtained through the MEDUSA selection strategy demonstrated significantly higher sequence diversity than the monovalent library.

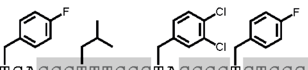

```
>m1  TCCTCAGGGTTTGGCTACGGGTCTGGCGG
>MSA5T   ACGGGTTTGGCGTCGGGCCTGGCGGGGGGATAGTGCGGT
>MSA52 TTACGTCAAGGTGTCACTCCGTAGGGTTTGGCTCCGGGCCTGGCGTCGGTCGTCTCTCGCGAAGCATCTCTTTGGCGTG
```

**Figure S10. Sequence alignment of m1 and reported SARS CoV-2 spike binding aptamers [8].**

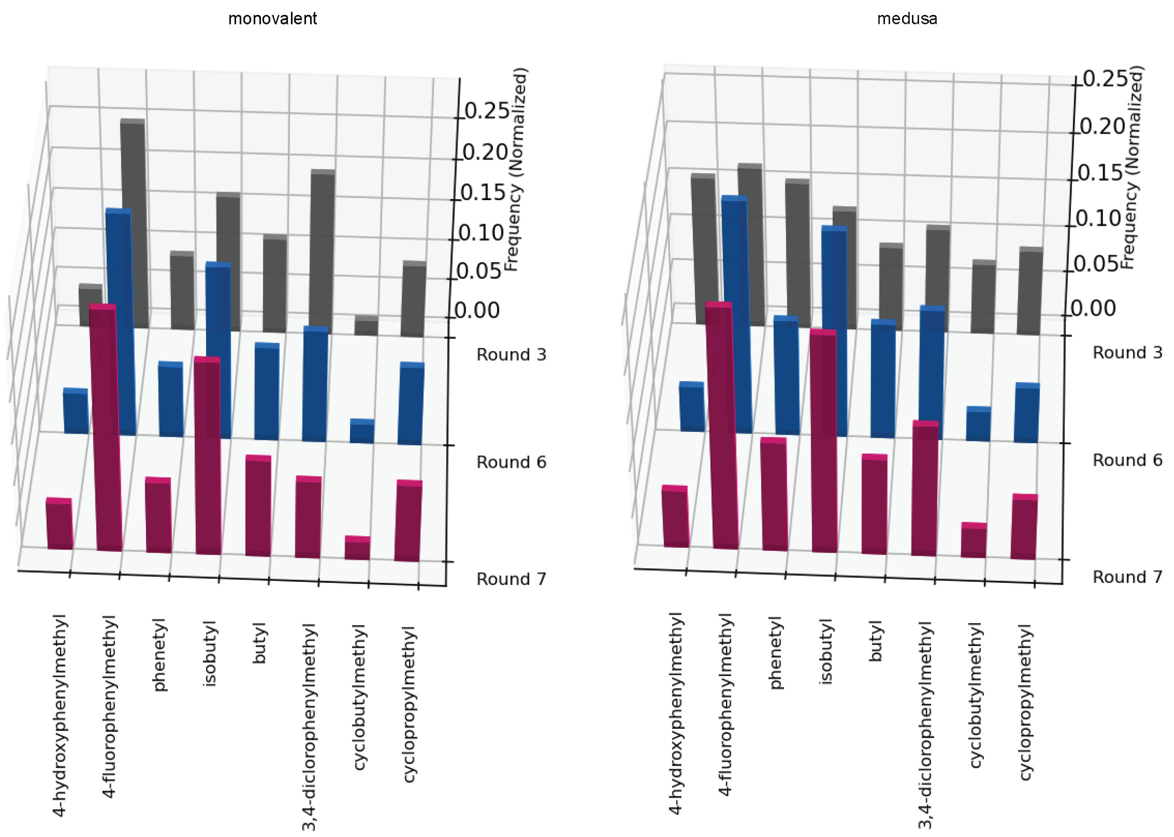


**Figure S11. Frequencies of side-chain modifications in the FNAP library at different round of selection for monovalent (left) and medusa (right) selection strategies.**

The frequencies of side-chain modifications were calculated from pre-filtered sequence counts using a custom Python script.

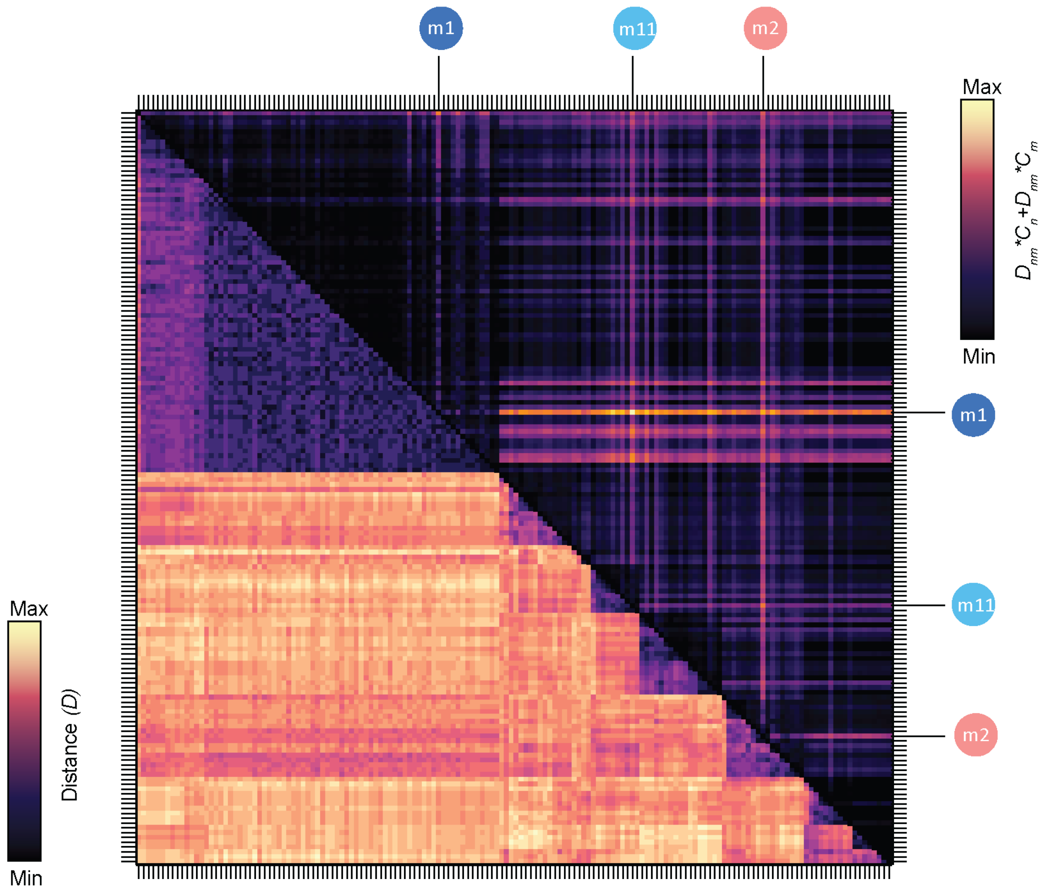


**Figure S12. Levenshtein distances between the top 150 selected sequences.**

(Top right triangle) sequence abundance-adjusted Levenshtein distances highlight the most abundant and dissimilar sequences.

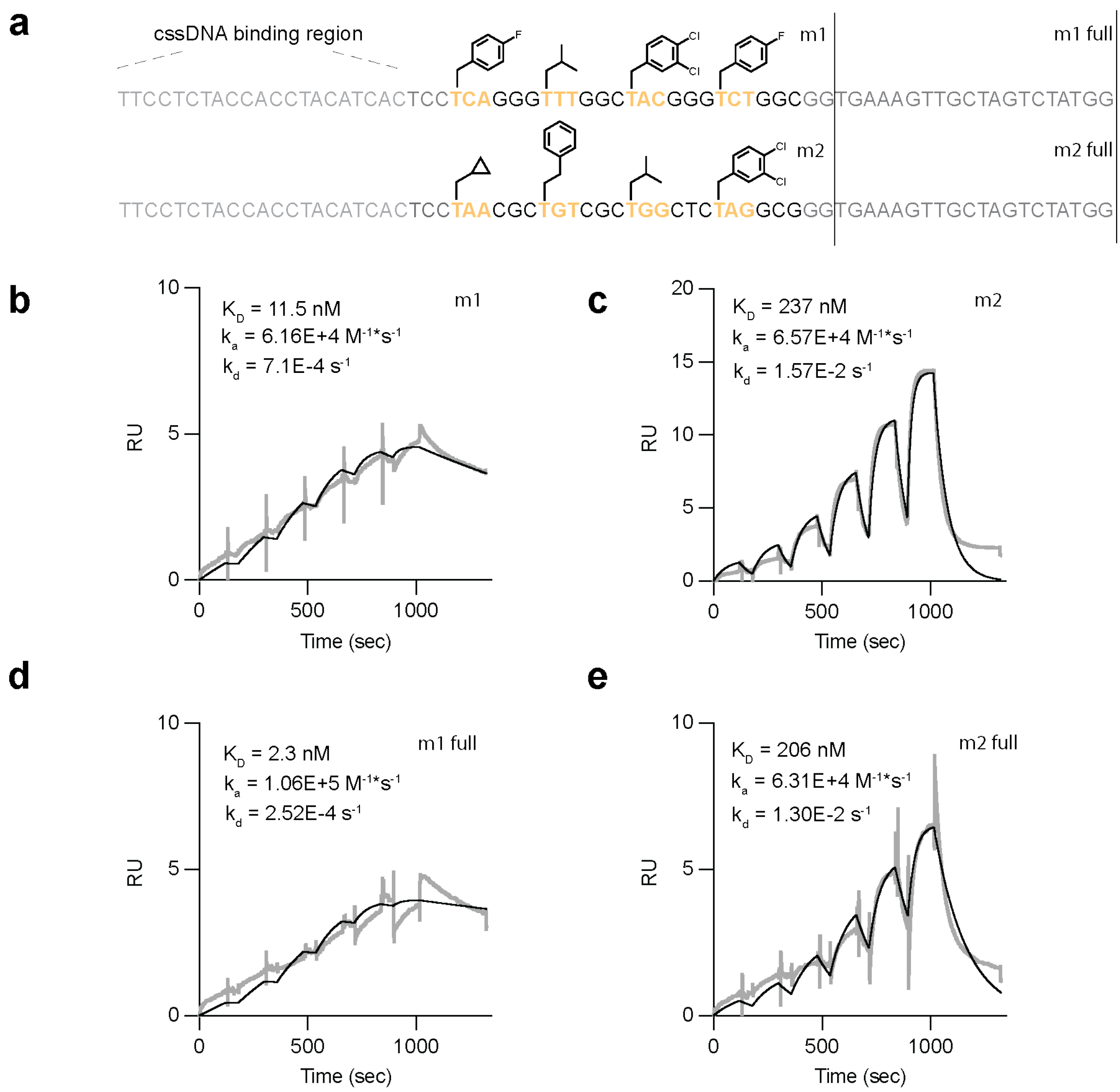


**Figure S13. Primer minimization study for m1 and m2 sequences.**

**a**, Variants of m1 and m2 sequences for primer minimization studies. SPR sensorgrams characterizing the binding kinetics between surface-immobilized trimeric SPIKE protein and monomeric forms of primer-minimized m1 (**b**), m2 (**c**) and full-length m1 full (**b**), m2 full (**e**) sequences. The concentrations of injected FNAPs were 18.75, 37.5, 75, 150, 300, and 600 nM.

| | Predicted MW | Observed MW | | |
|---|---|---|---|---|
| modified | 15907.35 | 15921 | 15923 | 15926 |
| non-modified | 15255.86 | 15269 | 15273 | 15272 |
| Average Δ | 651.49 | 652 | | |

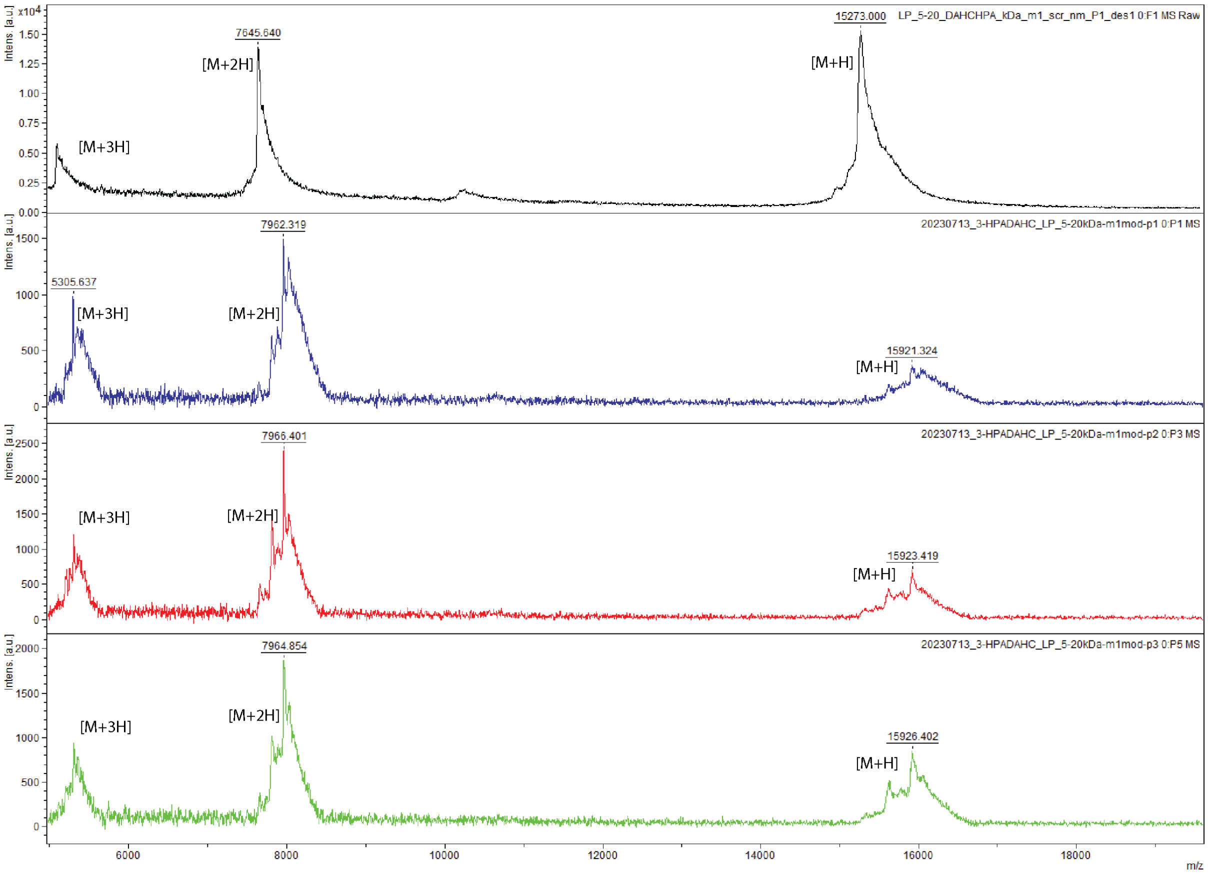


**Figure S14. Representative MALDI-TOF mass-spectra of PAGE-purified m1 binding units.** The correspondent scrambled non-modified control is plotted for reference.

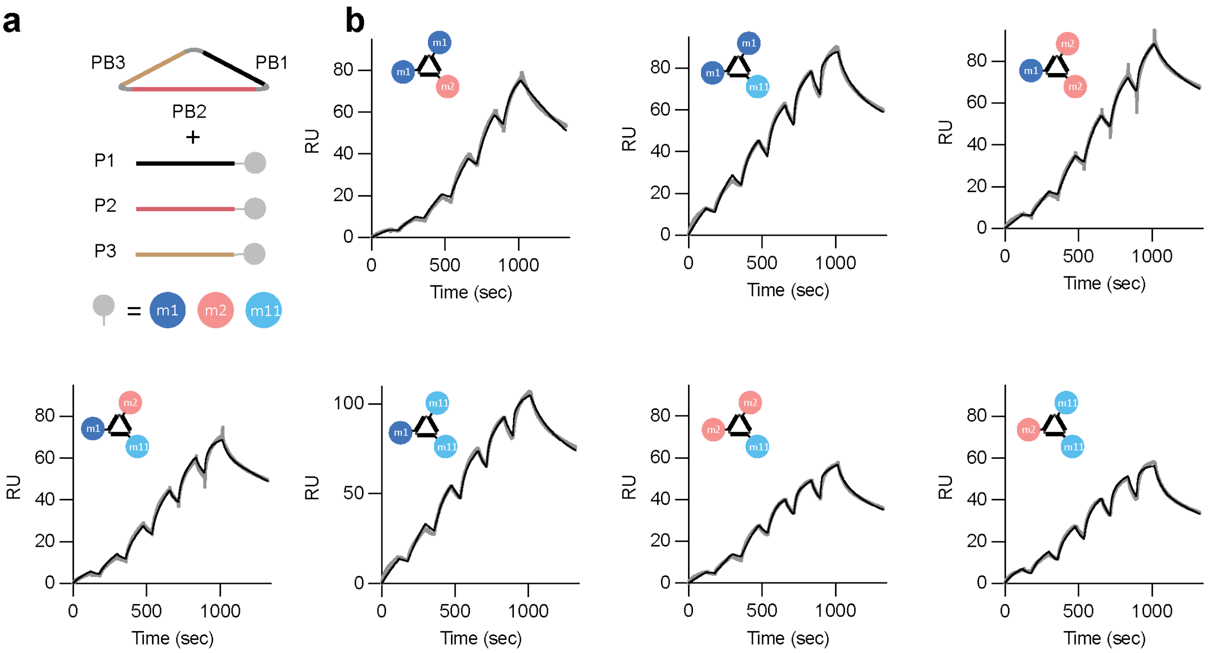


**c**

| P1 | P2 | P3 | $k_a$ ($M^{-1}$*$s^{-1}$) | $k_d$ ($s^{-1}$) | $K_D$ (nM) |
|---|---|---|---|---|---|
| m1 | m1 | m2 | 5.75E+4 | 1.35E-3 | 23.4 |
| m2 | m2 | m1 | 6.76E+4 | 8.48E-4 | 12.5 |
| m1 | m1 | m11 | 1.20E+5 | 1.34E-3 | 11.1 |
| m11 | m11 | m1 | 1.14E+5 | 1.07E-3 | 9.4 |
| m2 | m2 | m11 | 1.12E+5 | 1.62E-3 | 14.4 |
| m11 | m11 | m2 | 1.15E+5 | 1.95E-3 | 17.0 |
| m1 | m2 | m11 | 7.64E+4 | 1.17E-3 | 15.3 |

**Figure S15. Additional characterization of selected MEDUSAs.**

**a**, A schematic of preparation of hetero-trivalent MEDUSAs. **b**, SPR sensorgrams characterizing the binding kinetics between surface-immobilized trimeric SPIKE protein and hetero-trimeric MEDUSAs. The concentrations of injected assemblies were 9.375, 18.75, 37.5, 75, 150 and 300 nm. **c**, SPR binding kinetics parameters for hetero-trivalent FNAP assemblies prepared with combinations of m1, m2 and m11 FNAPs. The black curves represent the binding kinetics fit.

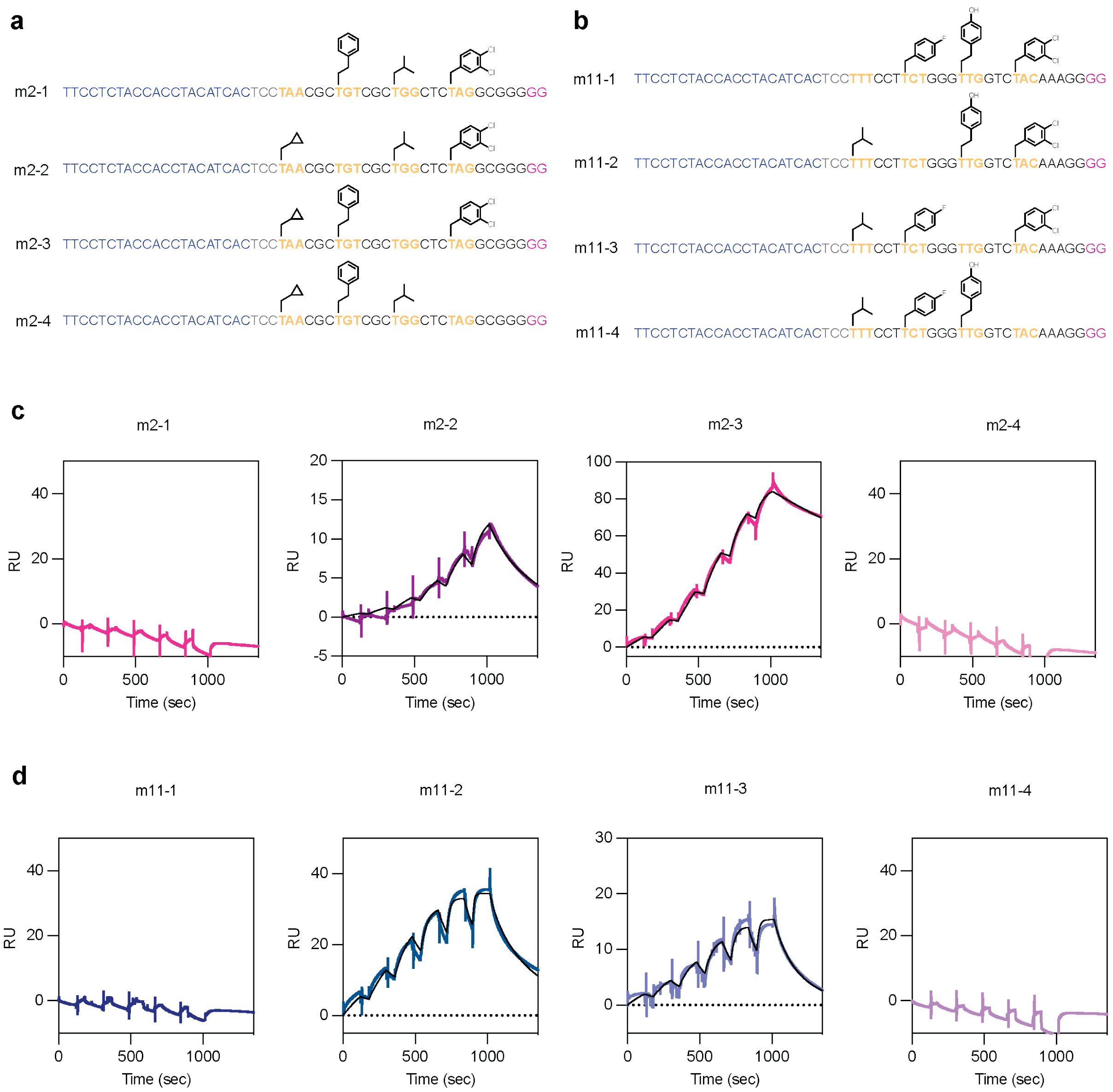


**Figure S16: Structure-activity study for m2 and m11 sequences.**

**a**, Sequences and side-chain modifications of side-chain-deficient variants of m2. **b**, Sequences and side-chain modifications of side-chain-deficient variants of m11. **c**, SPR sensorgrams of MEDUSAs prepared with side-chain-deficient variants of m2. **d**, SPR sensorgrams of MEDUSAs prepared with side-chain-deficient variants of m11. The concentrations of injected assemblies were: 9.375, 18.75, 37.5, 75, 150 and 300 nM.

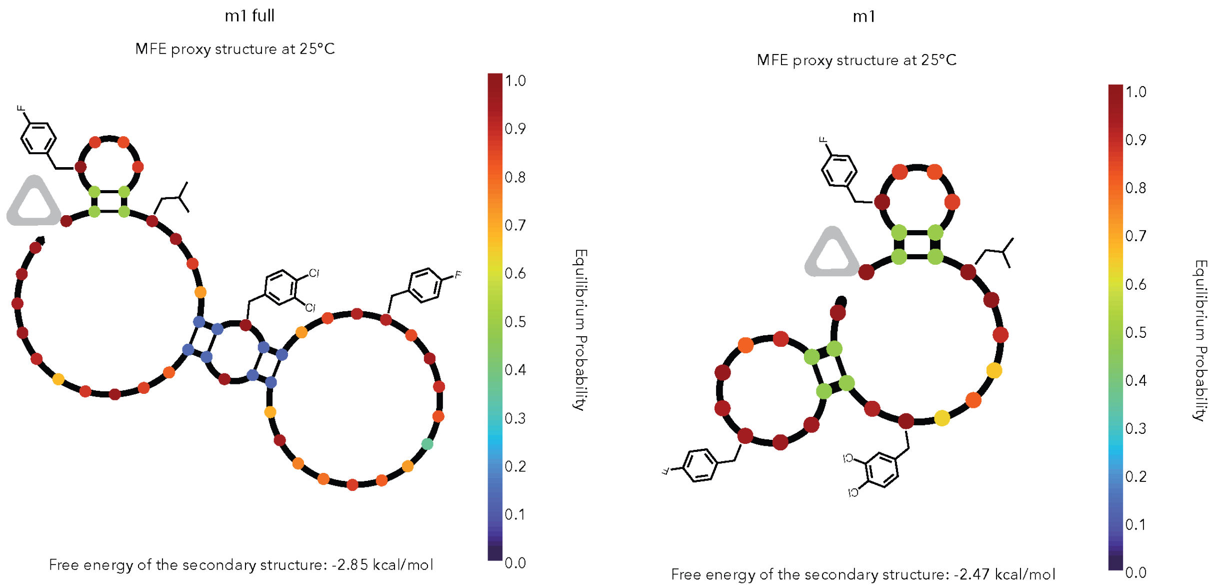


**Figure S17. Prediction of the secondary structure of the selected m1 FNAP using NUPACK.** The gray triangle indicates the FNAP's attachment point to the scaffold. Full-length (left) and primer-minimized (right) sequences are shown.

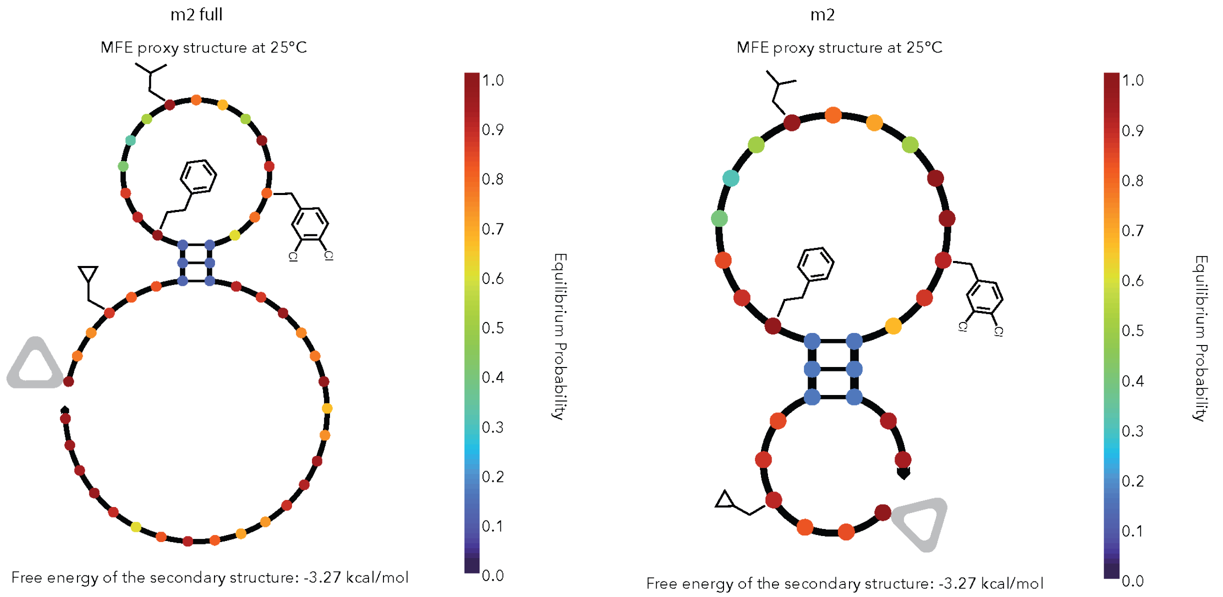


**Figure S18. Prediction of the secondary structure of the selected m2 FNAP using NUPACK.** The gray triangle indicates the FNAP's attachment point to the scaffold. Full-length (left) and primer-minimized (right) sequences are shown.

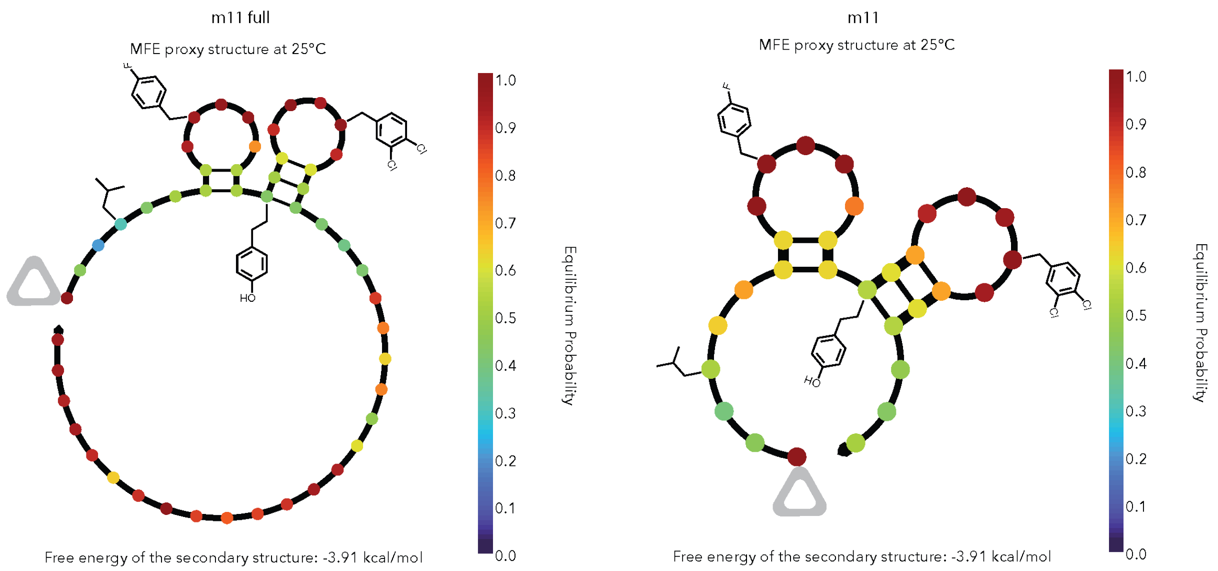


**Figure S19. Prediction of the secondary structure of the selected m11 FNAP using NUPACK.** The gray triangle indicates the FNAP's attachment point to the scaffold. Full-length (left) and primer-minimized (right) sequences are shown.

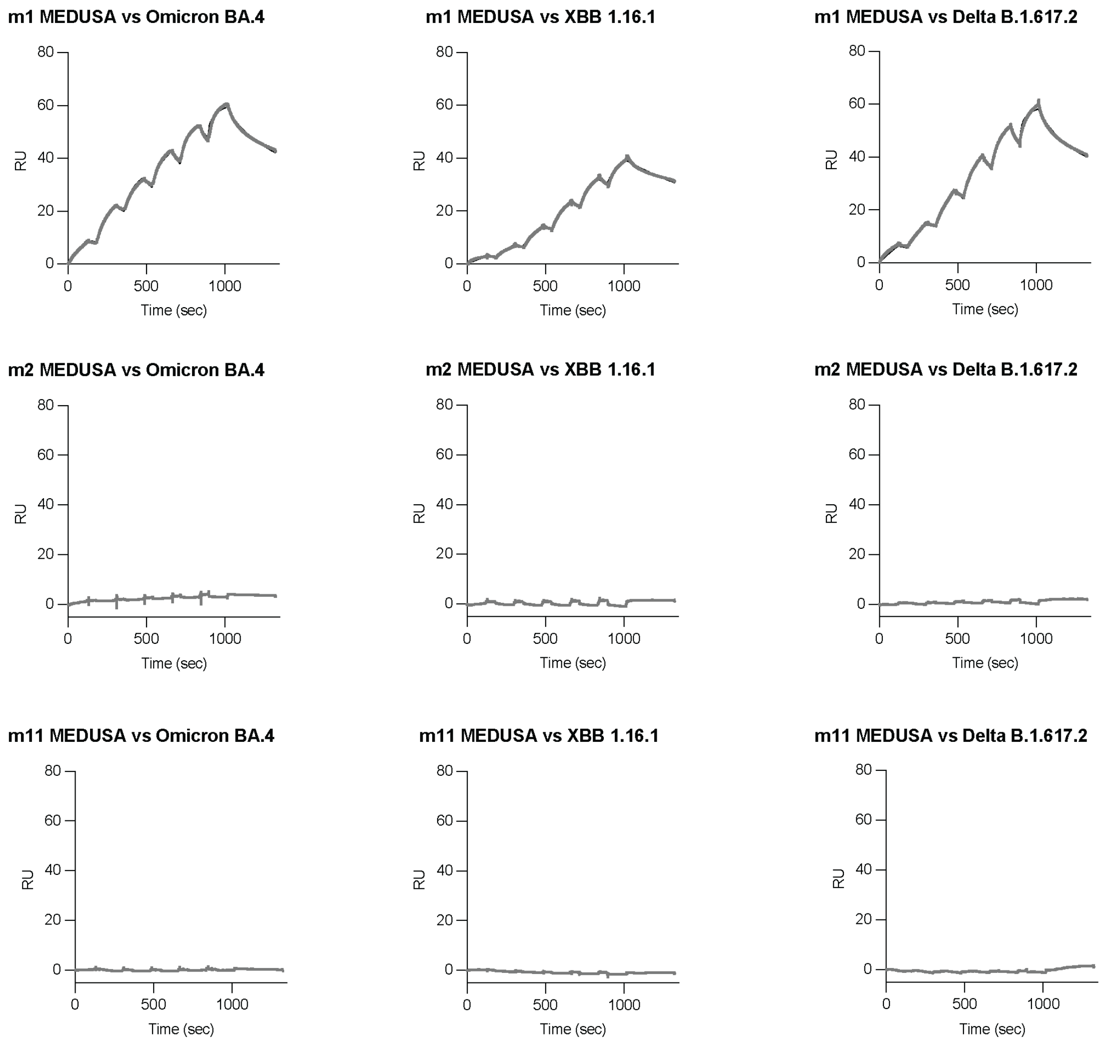


**Figure S20. Selectivity of selected MEDUSAs by SPR.**

The concentrations of injected assemblies were: 9.375, 18.75, 37.5, 75, 150 and 300 nM.

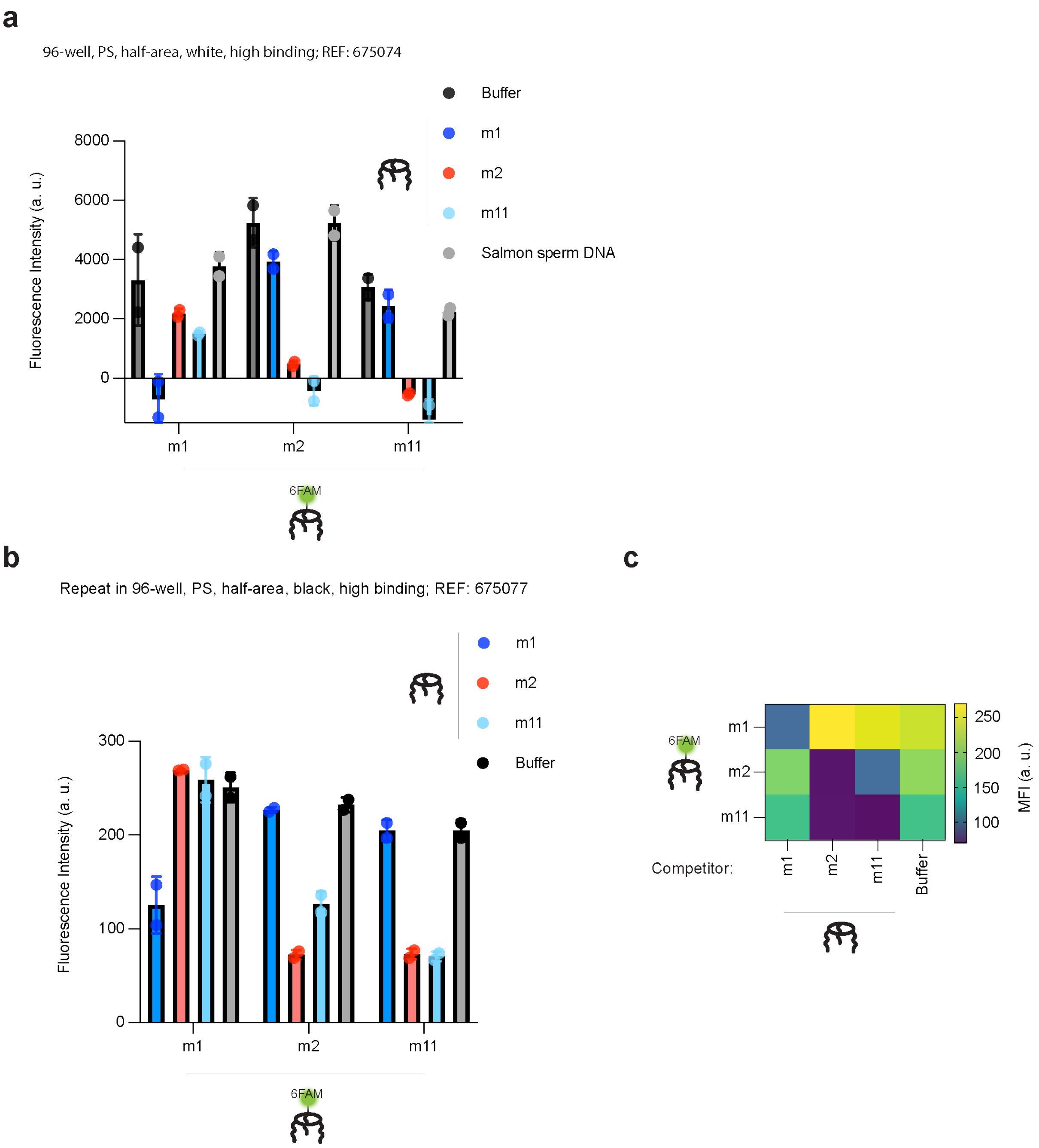


**Figure S21. Competition ELISA between trivalent assemblies using different ELISA plates.**

**a,** The data from Figure 4c are replotted as a bar plot for a better estimation of data variance. **b,** The replicate of the assay performed using a Greiner 96-well, PS, half-area, black, high-binding plate (REF: 675077). **c,** The data from panel **b** are plotted as a heatmap. All measurements were performed in duplicate (n = 2, technical replicates).

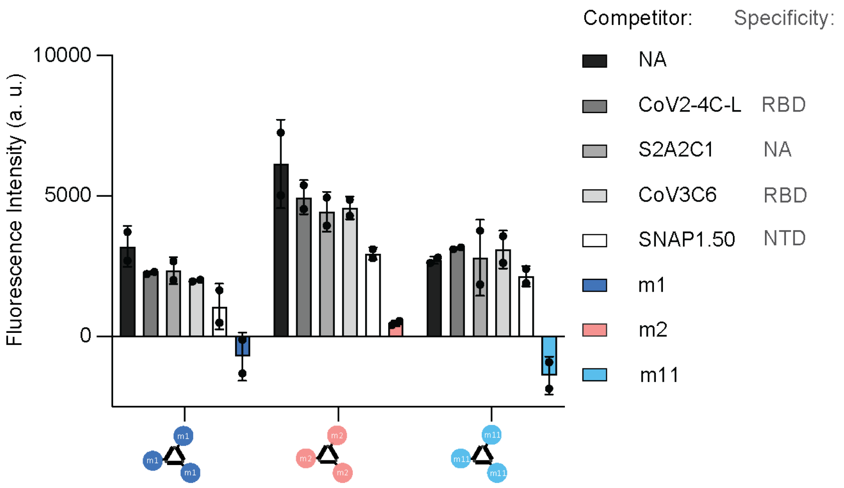


**Figure S22. Competition ELISA between MEDUSAs and a set of reported aptamers.**

The aptamer sequences are given in the Table S6. Note that for certain sequences (CoV6C3), off-target activity has been reported[9]. While this cannot be excluded for the other selected aptamers, it has not been reported to date. All measurements were performed in duplicate (n = 2, technical replicates).

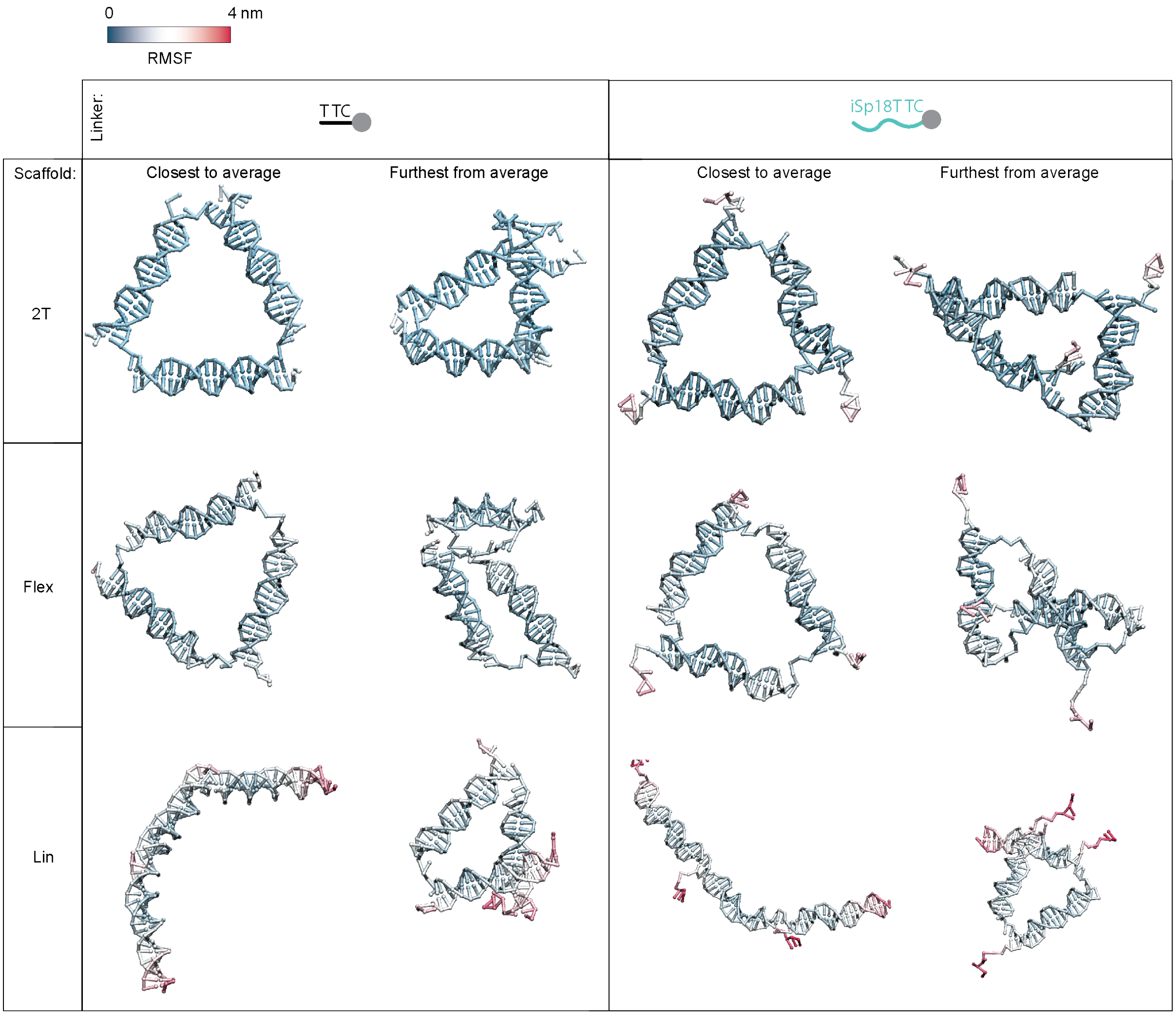


**Figure S23. OxDNA-generated models of assembly core prepared with 2T, iSp18 and Lin scaffold strands.** The iSp18 linker was simulated by 4 abasic nucleotides.

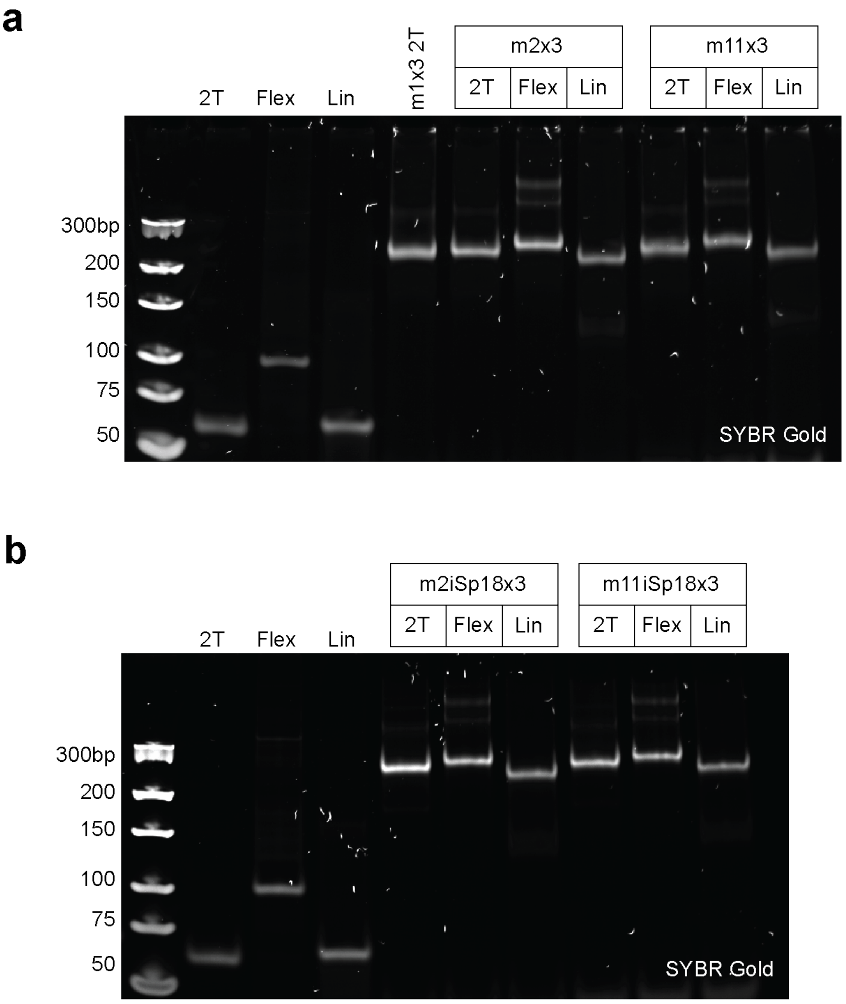


**Figure S24. PAGE analysis of MEDUSA variants featuring different binding units and scaffold strands.**

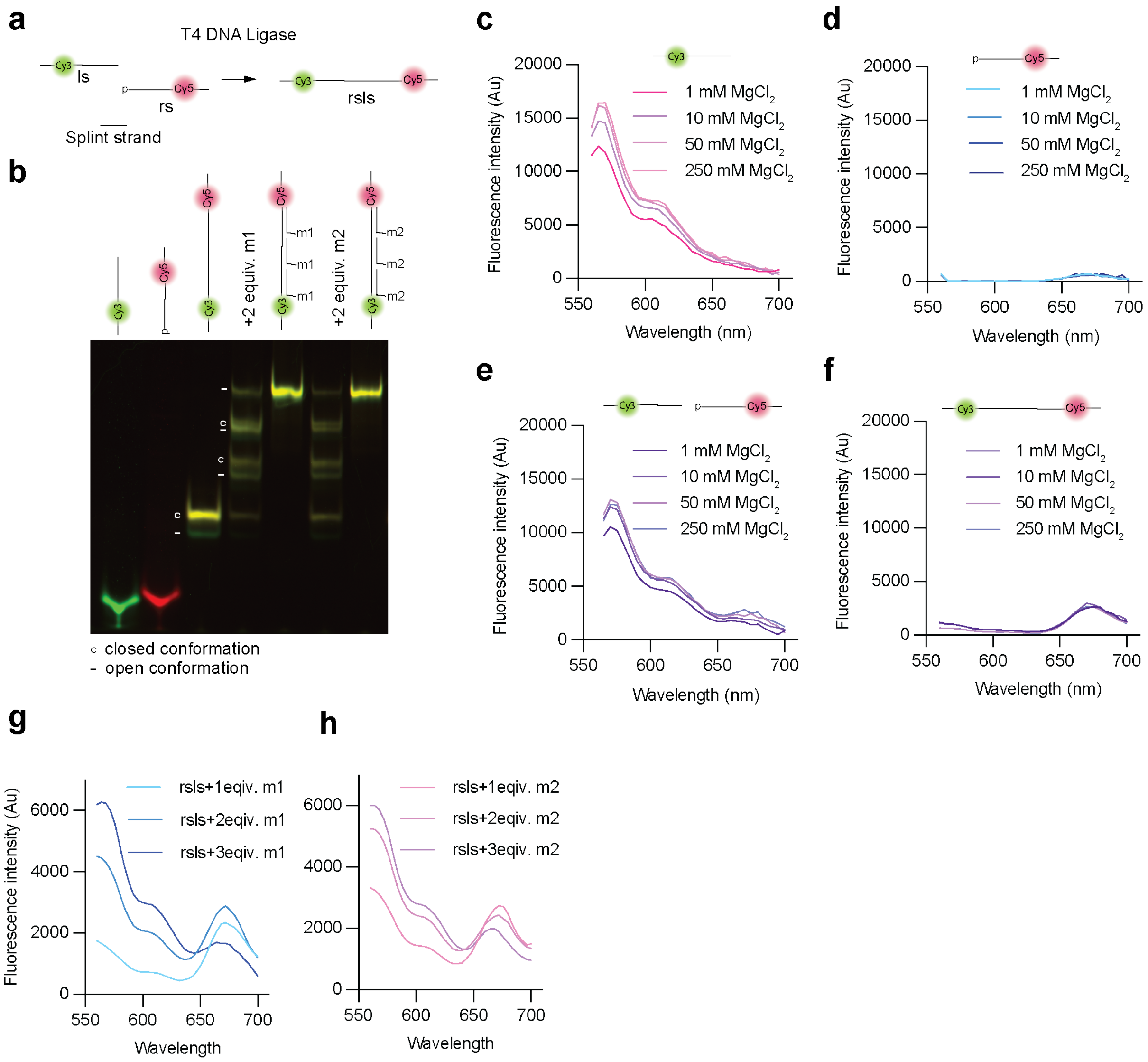


**Figure S25. sMEDUSA FRET construct production and characterization.**

**a**, T4 DNA Ligase-catalyzed preparation of the sensor's scaffold strand. **b**, Native 6% PAGE of m1 and m2 MEDUSA sensors. Fluorescence emission spectra for the left C€**(c)** and right Cy5 **(d)** parts of sensor's scaffold at different $MgCl_2$ concentrations. Fluorescence emission spectra for the mixture of left (Cy3) and right (Cy5) parts of sensor's sca€ld **(e)** and the sensor's scaffold strand **(f)** at different $MgCl_2$ concentrations. Spectral scans of sensor scaffold strand rsls hybridized with increasing amounts of m1 **(g)** and m2 (**h**) binding units. Upon hybridization, a flexible "rope-like" sensor scaffold transforms into a more rigid "nunchaku-like" structure, which results in the decrease in FRET efficiency. Excitation wavelength used for all emission spectra acquisitions: 550 nm.

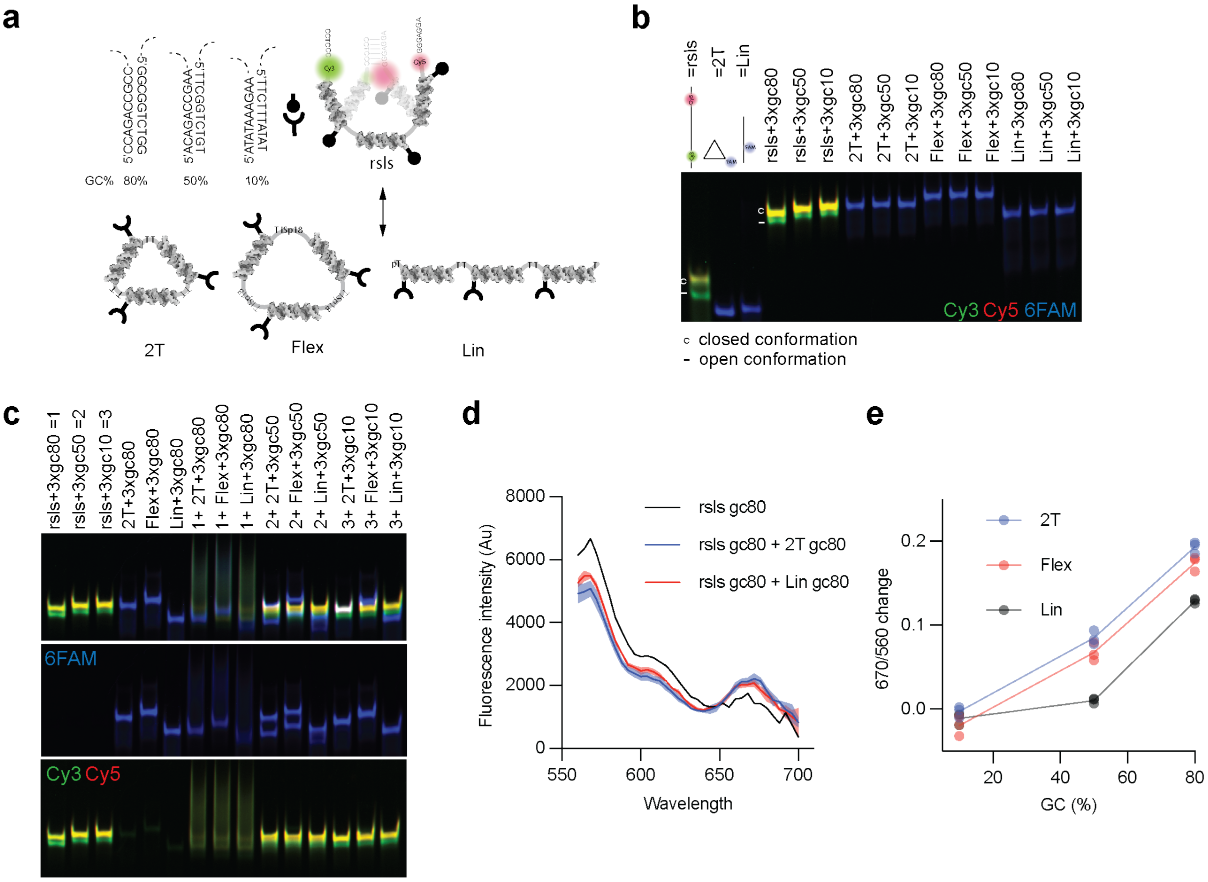

**Figure S26. FRET induction by cooperative multivalent binding in *cis* using guest sMEDUSA and host MEDUSAs of different scaffold configurations.**

**a**, A schematic of the experiment showing a set of “guest” sMEDUSAs with “binding units” featuring 80%, 50%, and 10% GC content. Correspondingly, a set of “host” MEDUSAs were prepared with three scaffold variants, each containing “binding units” complementary to those of the smEDUSAs. **b**, Native PAGE analysis of “guest” sMEDUSAs and “host” MEDUSAs with different scaffold and “binding unit” compositions. **c**, Native PAGE analysis of host-guest MEDUSA pairs mixed at a 1:1 molar ratio. **d**, Representative fluorescence intensity spectra for sMEDUSAs with 80% GC content “binding units” upon binding to corresponding 2T and Lin “host” MEDUSAs. **e**, The change in the 670/560 fluorescence intensity ratio relative to sMEDUSA in buffer for each host-guest MEDUSA pair.

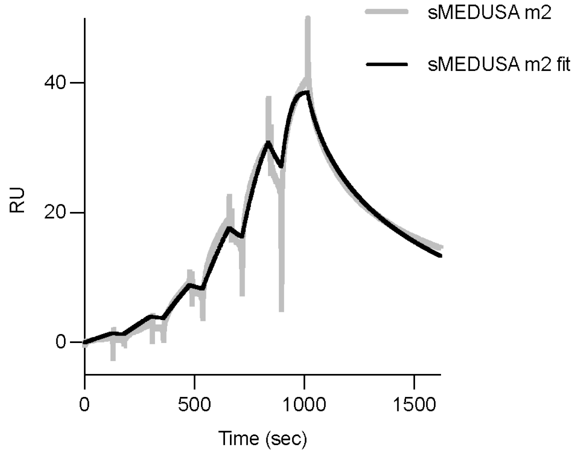


| Flow cell temp. (°C) | Kinetics model | Kinetics Chi² (RU²) | ka (1/Ms) | kd (1/s) | KD (M) | Rmax (RU) | tc | Spike immoblilized (RU) |
|---|---|---|---|---|---|---|---|---|
| 37 | 1:1 binding | 1.89 | 2.52E+05 | 7.12E-03 | 2.82E-08 | 42.3 | 6.18E+05 | 1882 |

**Figure S27. m2 sMEDUSA binding to the spike protein at 37 °C.**

The concentrations of injected assemblies were: 9.375, 18.75, 37.5, 75, 150 and 300 nM.

# Supplementary Tables

**Table S1. Modified trinucleotide building blocks.**

| Sequence | GC % | Modification | Modification's class |
|---|---|---|---|
| TAA | 0.00 | Cyclopropylmethyl | Aliphatic 3 carbon cycle |
| TCC | 66.67 | | |
| TAT | 0.00 | Cyclobutylmethyl | Alipatic 4 carbon cycle |
| TCG | 66.67 | | |
| TAC | 33.33 | 3,4-Diclorophenylmethyl | Aromatic chlorinated |
| TAG | 33.33 | | |
| TTA | 0.00 | Butyl | Aliphatic linear |
| TGC | 66.67 | | |
| TTT | 0.00 | Isobutyl | Aliphatic branched |
| TGG | 66.67 | | |
| TTC | 33.33 | Phenetyl | Aromatic |
| TGT | 33.33 | | |
| TCA | 33.33 | 4-Fluorobenzyl | Aromatic fluorinated |
| TCT | 33.33 | | |
| TGA | 33.33 | 4-Ethylphenol | Aromatic hydroxylated |
| TTG | 33.33 | | |

**Table S2. Primer sequences**

| | |
|---|---|
| * - phosphorothioation | |
| /iSp18/ - 18-atom hexa-ethyleneglycol spacer | |
| /52-Bio/ - dual biotin modification | |
| **ID** | **Sequence** |
| PCR primers | |
| A22/iSp18/scaffold primer | AAAAAAAAAAAAAAAAAAAAAA/iSp18/TTCCTCTACCACCTACATCAC |
| template primer Bio | /52-Bio//iSp18/CCATAGACTAGCAACTTTCACC |
| Translation primers | |
| initiating primer | T*T*C*C*T*CTACCACCTACATCACTCC |
| terminating primer | /5phos/GGTGAAAGTTGCTAGTCTATGG |
| Sequencing primers, Illumina adapters | |
| Illumina adapter+template primer | TCGTCGGCAGCGTCAGATGTGTATAAGAGACAGCCATAGACTAGCAACTTTCACC |
| Illumina adapter+initiation primer | GTCTCGTGGGCTCGGAGATGTGTATAAGAGACAGTTCCTCTACCACCTACATCACTCC |
| Sequencing left | TCGTCGGCAGCGTCAGATGT |
| Sequencing right | GTCTCGTGGGCTCGGAGATG |

**Table S4. Scaffold strand sequences**

| | |
|---|---|
| /iFluorT/ - Fluorescein is attached to position 5 of the thymine ring by a 6-carbon spacer arm. | |
| **ID** | **Sequence** |
| MEDUSA | |
| ssDNA 2T | /5phos/TGGTAGAGGAATTGTGATGTAGGTGGTAGAGGAATTGTGATGTAGGTGGTAGAGGAATTGTGATGTAGG |
| ssDNA 2T F | /5phos/TGGTAGAGGAATTGTGATGTAGGTGGTAGAGGAA/iFluorT/TGTGATGTAGGTGGTAGAGGAATTGTGATGTAGG |
| ssDNA Flex F | /5phos/TGGTAGAGGAAT/iSp18/GTGATGTAGGTGGTAGAGGAA/iFluorT//iSp18/GTGATGTAGGTGGTAGAGGAAT/iSp18/GTGATGTAGG |
| ssDNA 2T ort | /5phos/TGGTAGAGGAATTGAAAGTTAAGGGTAGTGTGGGTTGGAGGTTAAGGTGATGAGGATTTGTGATGTAGG |
| ssDNA 2T ort F | /5phos/TGGTAGAGGAATTGAAAGTTAAGGGTAGTGTGGG/iFluorT/TGGAGGTTAAGGTGATGAGGATTTGTGATGTAGG |
| ssDNA 2T Lin F | /5phos/TGTGATGTAGGTGGTAGAGGAA/iFluorT/TGTGATGTAGGTGGTAGAGGAATTGTGATGTAGGTGGTAGAGGAAT |
| splint | CCTCTACCACCTACATCAC |
| sMEDUSA | |
| medusa-sensor-rs | /5phos/GGTAGAGGAATTGTGATGTAGGTGGTAGAGGAAT/iCy5/GGGAGGA |

| | |
|---|---|
| medusa-sensor-ls | CCTCCC/iCy3/TGTGATGTAGGTGGTAGAGGAATTGTGATGTAGGT |
| medusa-sensor-ssDNA | CCTCCC/iCy3/TGTGATGTAGGTGGTAGAGGAATTGTGATGTAGGTGGTAGAGGAATTGT GATGTAGGTGGTAGAGGAAT/iCy5/GGGAGGA |

**Table S5. Binding unit sequences**

| ID | Sequence |
|---|---|
| Control sequences | |
| m1 snm P1 | TTCCTCTACCACCTACATCACTCCGGCTATGGCTTGCGGCGTGTGGTGAC |
| m1 snm P2 | CCCACACTACCCTTAACTTTCTCCGGCTATGGCTTGCGGCGTGTGGTGAC |
| m1 snm P3 | ATCCTCATCACCTTAACCTCCTCCGGCTATGGCTTGCGGCGTGTGGTGAC |
| m1 nm P1 | TTCCTCTACCACCTACATCACTCCTCAGGGTTTGGCTACGGGTCTGGCGG |
| m2 snm P1 | TTCCTCTACCACCTACATCACTCCACCTGGACCGTGCTCGGAGGTTGCTG |
| m2 snm P2 | CCCACACTACCCTTAACTTTCTCCACCTGGACCGTGCTCGGAGGTTGCTG |
| m2 snm P3 | ATCCTCATCACCTTAACCTCCTCCACCTGGACCGTGCTCGGAGGTTGCTG |
| m2 nm P1 | TTCCTCTACCACCTACATCACTCCTAACGCTGTCGCTGGCTCTAGGCGGG |
| m11 snm P1 | TTCCTCTACCACCTACATCACTCCATATTCGTGTTAGGTCGTGCTGCCAT |
| m11 snm P2 | CCCACACTACCCTTAACTTTCTCCATATTCGTGTTAGGTCGTGCTGCCAT |
| m11 snm P3 | ATCCTCATCACCTTAACCTCCTCCATATTCGTGTTAGGTCGTGCTGCCAT |
| m11 nm P1 | TTCCTCTACCACCTACATCACTCCTTTCCTTCTGGGTTGGTCTACAAAGG |
| Side-chain-functionalized sequences | |
| m1 full P1 | TTCCTCTACCACCTACATCACTCC(T-4-Fluorophenyl)CAGGG(T-Isobutyl)TTGGC(T-3,4-Diclorophenylmethyl)ACGGG(T-4-Fluorophenyl)CTGGCGGTGAAAGTTGCTAGTCTATGG |
| m2 full P1 | TTCCTCTACCACCTACATCACTCC(T-Cyclopropylmethyl)AACGC(T-Phenetyl)GTCGC(T-Isobutyl)GGCTC(T-3,4-Diclorophenylmethyl)AGGCGGGTGAAAGTTGCTAGTCTATGG |
| m11 full P1 | TTCCTCTACCACCTACATCACTCC(T-Isobutyl)TTCCT(T-4-Fluorophenyl)CTGGG(T-4-Ethylphenol)TGGTC(T-3,4-Diclorophenylmethyl)ACAAAGGTGAAAGTTGCTAGTCTATGG |
| m1 P1 | TTCCTCTACCACCTACATCACTCC(T-4-Fluorophenyl)CAGGG(T-Isobutyl)TTGGC(T-3,4-Diclorophenylmethyl)ACGGG(T-4-Fluorophenyl)CTGGCGG |
| m1 P2 | CCCACACTACCCTTAACTTTCTCC(T-4-Fluorophenyl)CAGGG(T-Isobutyl)TTGGC(T-3,4-Diclorophenylmethyl)ACGGG(T-4-Fluorophenyl)CTGGCGG |
| m1 P3 | ATCCTCATCACCTTAACCTCCTCC(T-4-Fluorophenyl)CAGGG(T-Isobutyl)TTGGC(T-3,4-Diclorophenylmethyl)ACGGG(T-4-Fluorophenyl)CTGGCGG |
| m2 P1 | TTCCTCTACCACCTACATCACTCC(T-Cyclopropylmethyl)AACGC(T-Phenetyl)GTCGC(T-Isobutyl)GGCTC(T-3,4-Diclorophenylmethyl)AGGCGGG |
| m2 P2 | CCCACACTACCCTTAACTTTCTCC(T-Cyclopropylmethyl)AACGC(T-Phenetyl)GTCGC(T-Isobutyl)GGCTC(T-3,4-Diclorophenylmethyl)AGGCGGG |
| m2 P3 | ATCCTCATCACCTTAACCTCCTCC(T-Cyclopropylmethyl)AACGC(T-Phenetyl)GTCGC(T-Isobutyl)GGCTC(T-3,4-Diclorophenylmethyl)AGGCGGG |
| m11 P1 | TTCCTCTACCACCTACATCACTCC(T-Isobutyl)TTCCT(T-4-Fluorophenyl)CTGGG(T-4-Ethylphenol)TGGTC(T-3,4-Diclorophenylmethyl)ACAAAGG |

| | |
|---|---|
| m11 P2 | CCCACACTACCCTTAACTTTCTCC(T-Isobutyl)TTCCT(T-4-Fluorophenyl)CTGGG(T-4-Ethylphenol)TGGTC(T-3,4-Diclorophenylmethyl)ACAAAGG |
| m11 P3 | ATCCTCATCACCTTAACCTCCTCC(T-Isobutyl)TTCCT(T-4-Fluorophenyl)CTGGG(T-4-Ethylphenol)TGGTC(T-3,4-Diclorophenylmethyl)ACAAAGG |
| m2 iSp18 P1 | TTCCTCTACCACCTACATCACT/iSp18/TCC(T-Cyclopropylmethyl)AACGC(T-Phenetyl)GTCGC(T-Isobutyl)GGCTC(T-3,4-Diclorophenylmethyl)AGGCGGG |
| m11 iSp18 P1 | TTCCTCTACCACCTACATCACT/iSp18/TCC(T-Isobutyl)TTCCT(T-4-Fluorophenyl)CTGGG(T-4-Ethylphenol)TGGTC(T-3,4-Diclorophenylmethyl)ACAAAGG |
| Side-chain-deficient sequences | |
| m2-4 P1 | TTCCTCTACCACCTACATCACTCC(T-Cyclopropylmethyl)AACGC(T-Phenetyl)GTCGC(T-Isobutyl)GGCTCTAGGCGGG |
| m2-3 P1 | TTCCTCTACCACCTACATCACTCC(T-Cyclopropylmethyl)AACGC(T-Phenetyl)GTCGCTGGCTC(T-3,4-Diclorophenylmethyl)AGGCGGG |
| m2-2 P1 | TTCCTCTACCACCTACATCACTCC(T-Cyclopropylmethyl)AACGCTGTCGC(T-Isobutyl)GGCTC(T-3,4-Diclorophenylmethyl)AGGCGGG |
| m2-1 P1 | TTCCTCTACCACCTACATCACTCCTAACGC(T-Phenetyl)GTCGC(T-Isobutyl)GGCTC(T-3,4-Diclorophenylmethyl)AGGCGGG |
| m11-4 P1 | TTCCTCTACCACCTACATCACTCC(T-Isobutyl)TTCCT(T-4-Fluorophenyl)CTGGG(T-4-Ethylphenol)TGGTCTACAAAGG |
| m11-3 P1 | TTCCTCTACCACCTACATCACTCC(T-Isobutyl)TTCCT(T-4-Fluorophenyl)CTGGGTTGGTC(T-3,4-Diclorophenylmethyl)ACAAAGG |
| m11-2 P1 | TTCCTCTACCACCTACATCACTCC(T-Isobutyl)TTCCTTCTGGG(T-4-Ethylphenol)TGGTC(T-3,4-Diclorophenylmethyl)ACAAAGG |
| m11-1 P1 | TTCCTCTACCACCTACATCACTCCTTTCCT(T-4-Fluorophenyl)CTGGG(T-4-Ethylphenol)TGGTC(T-3,4-Diclorophenylmethyl)ACAAAGG |

**Table S6. Aptamers**

| ID | Sequence | Ref. |
|---|---|---|
| CoV2-4C-L | ATCCAGAGTGACGCAGCATTTCATCGGGTCCAAAAGGGGCTGCTCGGGATTGCGGATATGGACACGTTT | [10] |
| S2A2C1 | AGGCGGGTTCCTAGACTTGTACTCAGCCT | [11] |
| CoV6C3 | CGCAGCACCCAAGAACAAGGACTGCTTAGGATTGCGATAGGTTCGG | [13] |
| SNAP1.50 | CGCGGTCATTGTGCATCCTGACTGACCCTAAGGTGCGAACATCGCCCGCG | [12] |

**Table S7. SPR kinetic parameters**

| Kinetics model | Assembly's ID | Binding unit composition (snm - scrambled non-modified) | Kinetics Chi² ($RU^2$) | ka (1/Ms) | kd (1/s) | KD (M) | Rmax (RU) | Spike immoblilized (RU) | tc |
|---|---|---|---|---|---|---|---|---|---|
| 1:1 binding | monovalent m1 assembly | m1 snm p1+m1p2+m1 snm p3 | 2.01E-01 | 7.70E+04 | 1.89E-03 | 2.45E-08 | 7.6 | 1307.2 | 5.01E+10 |
| 1:1 binding | bivalent m1 assembly | m1p1+m1p2+m1 snm p3 | 1.00E+00 | 1.27E+05 | 3.74E-04 | 2.96E-09 | 16.5 | 1231.7 | 5.28E+11 |
| 1:1 binding | trivalent m1 assembly | m1p1x3 | 1.38E+00 | 1.40E+05 | 9.20E-04 | 6.56E-09 | 19.8 | 1015.8 | 6.31E+11 |
| | | | | | | | | | |
| 1:1 binding | monovalent m2 assembly | m2 snm p1+m2p2+m2 snm p3 | 1.30E+00 | 8.65E+05 | 7.67E-02 | 8.86E-08 | 31.1 | 1357 | 1.00E+06 |
| 1:1 binding | bivalent m2 assembly | m2p1+m2p2+m2 snm p3 | 2.52E+00 | 5.83E+04 | 1.25E-03 | 2.14E-08 | 54.8 | 1425 | 1.03E+11 |
| 1:1 binding | trivalent m2 assembly | m2p1x3 | 1.20E+00 | 8.87E+04 | 1.38E-03 | 1.55E-08 | 49 | 1042.5 | 1.06E+11 |
| | | | | | | | | | |
| 1:1 binding | monovalent 11 assembly | m11 snm p1+m11p2+m11 snm p3 | NA | NA | NA | NA | 6.3 | 1331.8 | 4.02E+07 |
| 1:1 binding | bivalent m11 assembly | m11p1+m11p2+m11 snm p3 | 9.60E-01 | 1.53E+05 | 5.11E-03 | 3.34E-08 | 22.1 | 1198.9 | 1.01E+08 |
| 1:1 binding | trivalent m11 assembly | m11p1x3 | 3.94E+00 | 1.44E+05 | 3.51E-03 | 2.44E-08 | 5.25E+01 | 1229.7 | 8.89E+06 |
| | | | | | | | | | |
| 1:1 binding | | m1p1+m1p2+m2p3 | 1.59E+00 | 5.75E+04 | 1.35E-03 | 2.34E-08 | 83.9 | 1606.3 | 4.78E+06 |
| 1:1 binding | | m1p1+m2p2+m2p3 | 3.30E+00 | 6.76E+04 | 8.48E-04 | 1.25E-08 | 89.8 | 1437 | 3.37E+12 |
| 1:1 binding | | m1p1+m1p2+m11p3 | 8.50E+00 | 1.20E+05 | 1.34E-03 | 1.11E-08 | 86.6 | 1350.8 | 2.84E+12 |
| 1:1 binding | | m1p1+m11p2+m11p3 | 1.06E+01 | 1.14E+05 | 1.07E-03 | 9.42E-09 | 102.6 | 1648.9 | 2.63E+12 |
| 1:1 binding | | m2p1+m2p2+m11p3 | 2.54E+00 | 1.13E+05 | 1.62E-03 | 1.44E-08 | 56.2 | 1261.6 | 3.36E+07 |
| 1:1 binding | | m2p1+m11p2+m11p3 | 3.36E+00 | 1.15E+05 | 1.95E-03 | 1.70E-08 | 57.7 | 1324 | 1.17E+11 |
| 1:1 binding | | m1p1+m2p2+m11p3 | 2.62E+00 | 7.64E+04 | 1.17E-03 | 1.53E-08 | 71.1 | 1313 | 6.66E+10 |
| 1:1 binding | | m11p1x3 | 3.94E+00 | 1.44E+05 | 3.51E-03 | 2.44E-08 | 52.5 | 1229.7 | 8.89E+06 |

<Table 7 continues the next page>

| Kinetics model | Assembly's ID | Binding unit composition (snm - scrambled non-modified) | Kinetics Chi² ($RU^2$) | ka1 (1/Ms) | kd1 (1/s) | ka2 (1/Ms) | kd2 (1/s) | Rmax1 (RU) | Rmax2 (RU) | KD1 (M) | KD2 (M) | Spike immoblilized (RU) |
|---|---|---|---|---|---|---|---|---|---|---|---|---|
| Heterogeneous ligand | monovalent m1 assembly | m1 scr p1+m1p2+m1 snm p3 | 1.55E-01 | 1.41E+05 | 1.15E-03 | 1.35E+03 | 3.96E-03 | 4.20E+00 | 6.71E+01 | 8.14E-09 | 2.94E-06 | 1307.2 |
| Heterogeneous ligand | bivalent m1 assembly | m1p1+m1p2+m1 snm p3 | 1.49E-01 | 4.86E+05 | 6.43E-07 | 5.01E+04 | 9.76E-04 | 6.32E+00 | 1.25E+01 | 1.32E-12 | 1.95E-08 | 1231.7 |
| Heterogeneous ligand | trivalent m1 assembly | m1p1x3 | 9.96E-02 | 1.80E+06 | 3.75E-03 | 6.44E+04 | 3.21E-04 | 5.59E+00 | 1.52E+01 | 2.08E-09 | 4.98E-09 | 1015.8 |
| | | | | | | | | | | | | |
| Heterogeneous ligand | monovalent 2 assembly | m2 scr p1+m2p2+m2 snm p3 | 8.06E-02 | 2.29E+05 | 2.47E-06 | 6.30E+04 | 2.29E-02 | 4.61E+00 | 4.73E+01 | 1.08E-11 | 3.64E-07 | 1357 |
| Heterogeneous ligand | bivalent m2 assembly | m2p1+m2p2+m2 snm p3 | 2.16E-01 | 6.42E+05 | 1.29E-02 | 2.88E+04 | 4.54E-07 | 1.95E+01 | 4.07E+01 | 2.01E-08 | 1.58E-11 | 1425 |
| Heterogeneous ligand | trivalent m2 assembly | m2p1x3 | 3.20E-01 | 3.48E+05 | 1.04E-02 | 6.31E+04 | 2.81E-04 | 1.32E+01 | 3.64E+01 | 2.99E-08 | 4.45E-09 | 1042.5 |
| | | | | | | | | | | | | |
| Heterogeneous ligand | monovalent m11 assembly | m11 snm p1+m11p2+m11 snm p3 | 5.22E-02 | 1.73E+05 | 3.93E-06 | 3.96E+04 | 1.99E-01 | 3.82E+00 | 6.19E+01 | 2.27E-11 | 5.04E-06 | 1331.8 |
| Heterogeneous ligand | bivalent m11 assembly | m11p1+m11p2+m11 snm p3 | 2.49E-01 | 5.90E+05 | 1.47E-02 | 4.19E+04 | 1.88E-03 | 1.16E+01 | 1.29E+01 | 2.48E-08 | 4.49E-08 | 1198.9 |
| Heterogeneous ligand | trivalent m11 assembly | m11p1x3 | 5.87E-01 | 6.03E+05 | 1.47E-02 | 3.51E+04 | 3.24E-07 | 3.42E+01 | 2.21E+01 | 2.44E-08 | 9.23E-12 | 1229.7 |
| | | | | | | | | | | | | |
| Heterogeneous ligand | | m1p1+m1p2+m2p3 | 1.59E+00 | 4.23E+05 | 1.20E-03 | 5.75E+04 | 1.35E-03 | 2.70E-03 | 8.39E+01 | 2.82E-09 | 2.34E-08 | 1606.3 |
| Heterogeneous ligand | | m1p1+m2p2+m2p3 | 1.11E+00 | 4.75E+05 | 3.93E-03 | 3.14E+04 | 3.68E-07 | 4.50E+01 | 5.25E+01 | 8.27E-09 | 1.17E-11 | 1437 |
| Heterogeneous ligand | | m1p1+m1p2+m11p3 | 1.52E+00 | 9.83E+05 | 7.71E-03 | 5.06E+04 | 5.92E-07 | 4.16E+01 | 4.92E+01 | 7.84E-09 | 1.17E-11 | 1350.8 |
| Heterogeneous ligand | | m1p1+m11p2+m11p3 | 2.27E+00 | 8.10E+05 | 5.22E-03 | 4.65E+04 | 3.70E-07 | 5.22E+01 | 5.65E+01 | 6.44E-09 | 7.95E-12 | 1648.9 |
| Heterogeneous ligand | | m2p1+m2p2+m11p3 | 5.31E-01 | 1.61E+06 | 1.46E-02 | 3.52E+04 | 2.33E-06 | 3.81E+01 | 2.25E+01 | 9.06E-09 | 6.61E-11 | 1261.6 |
| Heterogeneous ligand | | m2p1+m11p2+m11p3 | 8.32E-01 | 6.53E+05 | 2.03E-02 | 8.56E+04 | 9.25E-04 | 1.31E+01 | 4.62E+01 | 3.11E-08 | 1.08E-08 | 1324 |
| Heterogeneous ligand | | m1p1+m2p2+m11p3 | 1.17E+00 | 5.27E+05 | 2.74E-02 | 6.72E+04 | 7.22E-04 | 8.64E+00 | 6.44E+01 | 5.20E-08 | 1.07E-08 | 1313 |
| Heterogeneous ligand | | m11p1x3 | 5.87E-01 | 6.03E+05 | 1.47E-02 | 3.51E+04 | 3.24E-07 | 3.42E+01 | 2.21E+01 | 2.44E-08 | 9.23E-12 | 1229.7 |

# References


1. Liese, S. & Netz, R. R. Quantitative Prediction of Multivalent Ligand–Receptor Binding Affinities for Influenza, Cholera, and Anthrax Inhibition. *ACS Nano* **12**, 4140–4147 (2018).
2. Gillespie, D. T. Stochastic Simulation of Chemical Kinetics. *Annu. Rev. Phys. Chem.* **58**, 35–55 (2007).
3. Snodin, B. E. K. *et al.* Introducing improved structural properties and salt dependence into a coarse-grained model of DNA. *The Journal of Chemical Physics* **142**, 234901 (2015).
4. Poppleton, E. *et al.* Design, optimization and analysis of large DNA and RNA nanostructures through interactive visualization, editing and molecular simulation. *Nucleic Acids Research* **48**, e72–e72 (2020).
5. Wong, S. H. *et al.* Modulating the DNA/Lipid Interface through Multivalent Hydrophobicity. *Nano Lett.* **24**, 11210–11216 (2024).
6. Morzy, D. *et al.* Interplay of the mechanical and structural properties of DNA nanostructures determines their electrostatic interactions with lipid membranes. *Nanoscale* **15**, 2849–2859 (2023).
7. Zhang, Y., Juhas, M. & Kwok, C. K. Aptamers targeting SARS-COV-2: a promising tool to fight against COVID-19. *Trends in Biotechnology* **41**, 528–544 (2023).
8. Zhang, Z. *et al.* High-Affinity Dimeric Aptamers Enable the Rapid Electrochemical Detection of Wild-Type and B.1.1.7 SARS-CoV-2 in Unprocessed Saliva. *Angew Chem Int Ed* **60**, 24266–24274 (2021).
9. Civit, L. *et al.* A Multi-Faceted Binding Assessment of Aptamers Targeting the SARS-CoV-2 Spike Protein. *IJMS* **25**, 4642 (2024).
10. Zhang, J. *et al.* Elucidating the Effect of Nanoscale Receptor-Binding Domain Organization on SARS-CoV-2 Infection and Immunity Activation with DNA Origami. *J. Am. Chem. Soc.* jacs.2c09229 (2022) doi:10.1021/jacs.2c09229.
11. Silwal, A. P. *et al.* DNA aptamers inhibit SARS-CoV-2 spike-protein binding to hACE2 by an RBD- independent or dependent approach. *Theranostics* **12**, 5522–5536 (2022).
12. Kacherovsky, N. *et al.* Discovery and Characterization of Spike N-Terminal Domain-Binding Aptamers for Rapid SARS-CoV-2 Detection. *Angew Chem Int Ed* **60**, 21211–21215 (2021).
