# Supplementary figures and images for: Evolution of multivalent supramolecular assemblies of aptamers with target-defined spatial organization
Source: Nat Nanotechnol. 2025 Jun 6;20(8):1087–97. doi: 10.1038/s41565-025-01939-8 (PMC12373506; doi:10.1038/s41565-025-01939-8)

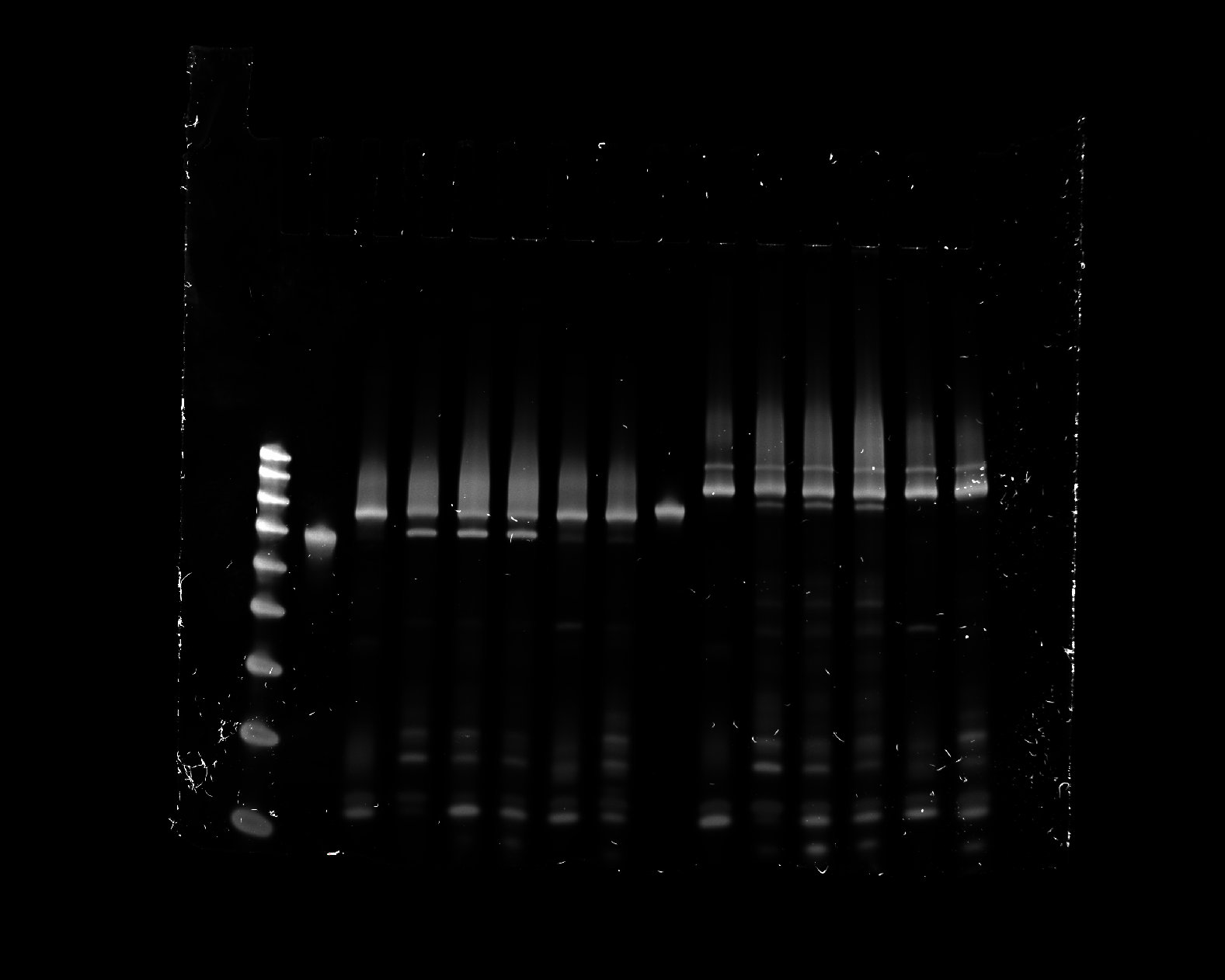

Supplement: Supplementary file 2 — Source Data Fig. 1. Numerical source data for panels b–d. Source Data Fig. 2. Full-length, unprocessed image of the gel in panels c and d. Source Data Fig. 3. Numerical source data for panels a, c–e. Source Data Fig. 4. Numerical source data for panels a–f. Source Data Fig. 5. Numerical source data for panels c–e, g–j. [file 41565_2025_1939_MOESM2_ESM.zip › Source data/Source data Figure 2/Fig_2c_raw.tif]

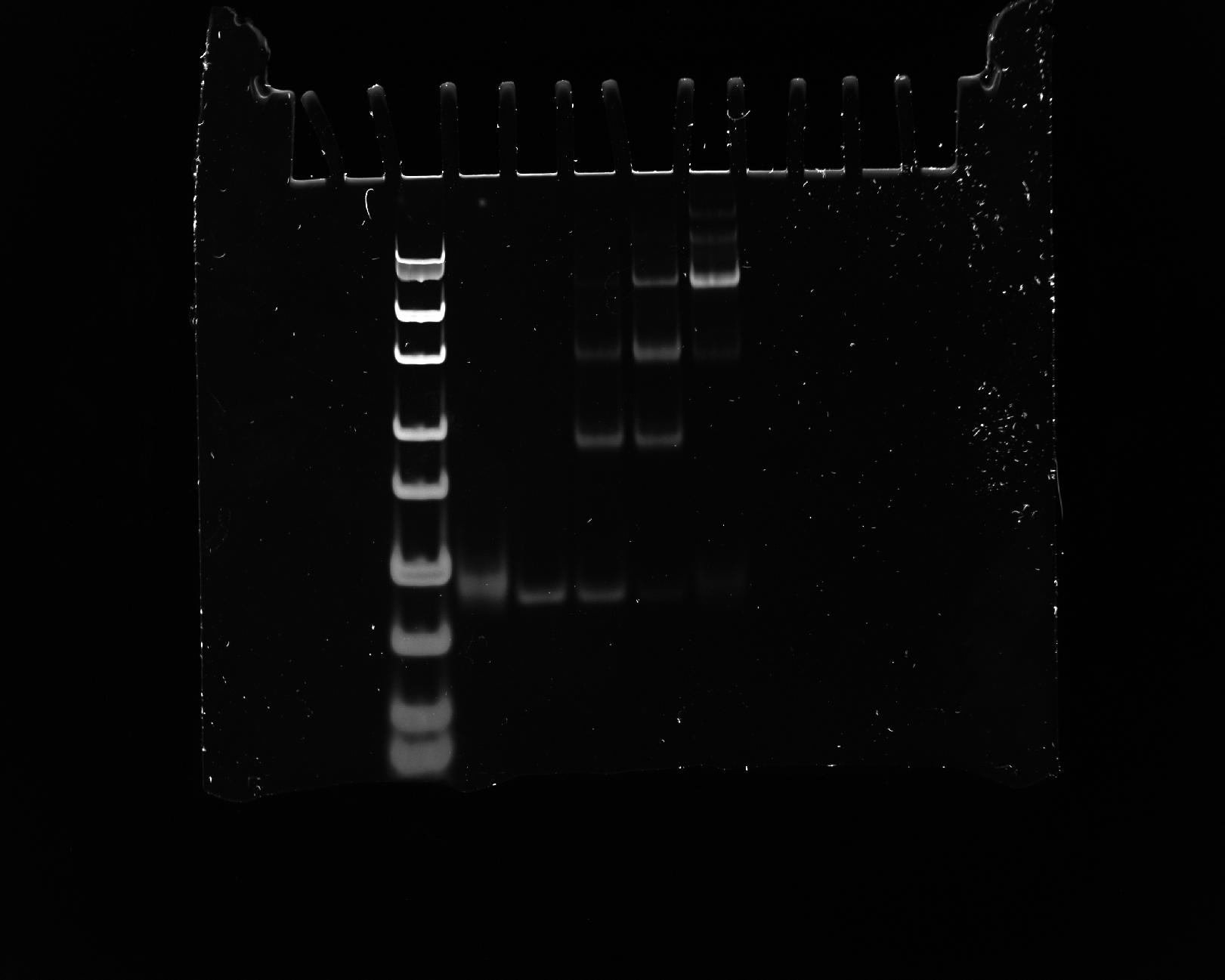

Supplement: Supplementary file 2 — Source Data Fig. 1. Numerical source data for panels b–d. Source Data Fig. 2. Full-length, unprocessed image of the gel in panels c and d. Source Data Fig. 3. Numerical source data for panels a, c–e. Source Data Fig. 4. Numerical source data for panels a–f. Source Data Fig. 5. Numerical source data for panels c–e, g–j. [file 41565_2025_1939_MOESM2_ESM.zip › Source data/Source data Figure 2/Fig_2d_raw.tif]
